# Unlocking the bacterial and fungal communities assemblages of sugarcane microbiome

**DOI:** 10.1038/srep28774

**Published:** 2016-06-30

**Authors:** Rafael Soares Correa de Souza, Vagner Katsumi Okura, Jaderson Silveira Leite Armanhi, Beatriz Jorrín, Núria Lozano, Márcio José da Silva, Manuel González-Guerrero, Laura Migliorini de Araújo, Natália Cristina Verza, Homayoun Chaichian Bagheri, Juan Imperial, Paulo Arruda

**Affiliations:** 1 Centro de Biologia Molecular e Engenharia Genética, Universidade Estadual de Campinas (UNICAMP), 13083-875, Campinas, SP, Brazil; 2 Centro de Biotecnología y Genómica de Plantas, Universidad Politécnica de Madrid (UPM) - Instituto Nacional de Investigación y Tecnología Agraria y Alimentaria (INIA Campus Montegancedo UPM, 28223 -Pozuelo de Alarcón (Madrid), Spain; 3 New Energies Division, Repsol Technology Center, 28935 Mostoles-Madrid, Spain; 4 Consejo Superior de Investigaciones Científicas, Madrid, Spain; 5 Departamento de Genética e Evolução, Instituto de Biologia, Universidade Estadual de Campinas (UNICAMP), 13083-970, Campinas, SP, Brazil

## Abstract

Plant microbiome and its manipulation herald a new era for plant biotechnology with the potential to benefit sustainable crop production. However, studies evaluating the diversity, structure and impact of the microbiota in economic important crops are still rare. Here we describe a comprehensive inventory of the structure and assemblage of the bacterial and fungal communities associated with sugarcane. Our analysis identified 23,811 bacterial OTUs and an unexpected 11,727 fungal OTUs inhabiting the endophytic and exophytic compartments of roots, shoots, and leaves. These communities originate primarily from native soil around plants and colonize plant organs in distinct patterns. The sample type is the primary driver of fungal community assemblage, and the organ compartment plays a major role in bacterial community assemblage. We identified core bacterial and fungal communities composed of less than 20% of the total microbial richness but accounting for over 90% of the total microbial relative abundance. The roots showed 89 core bacterial families, 19 of which accounted for 44% of the total relative abundance. Stalks are dominated by groups of yeasts that represent over 12% of total relative abundance. The core microbiome described here comprise groups whose biological role underlies important traits in plant growth and fermentative processes.

For many decades, isolated microbes naturally associated with plants have been shown to play fundamental roles in plants’ growth, development and health^1^,^2^,^3^. Recent advances in microbiome studies have revealed far more diverse and complex modes of microbe-plant association than previously acknowledged^4^,^5^,^6^,^7^ and have opened the field for microbiome-derived technologies applied for agriculture and industrial biotechnology^8^,^9^,^10^. Beneficial microbes have long been isolated from economically important crops. Sugarcane associated microbes, for example, have been investigated for decades for their nitrogen fixing and plant growth-promoting potential^11^. However, most of these studies have solely relied on isolation of target groups using defined culture media, which is known to sample a minute portion of total microbial diversity. As a result, we lack fundamental information such the real diversity of microbial community associated to sugarcane organs, factors driving community assemblage, origin of microbial community and correlation of known and unknown groups regarding abundance. Answering these fundamental questions is an imperative step towards understating the impact of microbial community in traits underlying sugarcane growth and its usage for biofuel production.

Although sugarcane has been traditionally cultivated for sugar production, it has emerged in the past few decades as one of the best crops for biofuel production^12^. As the economic importance of sugarcane increases, so do the requirements for increased productivity in an environmentally sustainable manner. However, the massive implantation of the crop imposes threats on sustainability as a result of increased needs for water and fertilizers^13^. The need to equilibrate productivity with sustainability has encouraged the development of alternatives for enhancing crop yield. In this context, the plant-associated microbiome has emerged as a potential alternative, allowing the exploration of the untapped diversity for modulating growth, development, pathogen defense, nutrient acquisition and stress resistance^1^,^9^,^10^,^14^.

Bacteria isolated from sugarcane rhizosphere have evidenced a self-supporting microbial ecosystem capable of sustain nitrogen fixation under filed conditions^15^,^16^,^17^,^18^. Studies have targeted primarily diazotrophic bacterial groups, such as *Beijerinckia*, *Gluconacetobacter*, *Azospirillum*, and *Herbaspirillum*^19^,^20^,^21^,^22^ by means of isolation and cultivation approaches. Bacterial communities have been accessed using Sanger sequencing in root system^23^,^24^,^25^ and leaves^24^ of sugarcane plants inoculated with selected diazotrophs and under different nitrogen fertilization. The analyses confirm the presence of inoculated and new diazotrophs. Field grown sugarcane plants under high and reduced nitrogen regime had the bacterial community accessed in root system^26^. A fertilization-independent core community was identified among diverse groups suggesting the enrichment of bacteria with potential plant benefit. Among the enriched root bacterial community, a new species of Burkholderia was identified and shown to stimulate sugarcane growth^27^.

Bioethanol production by fermentative processing sugarcane molasses is the second largest source of biofuel production in the world. Fermentative processes for bioethanol production based on sugarcane feedstock take place in open, non-sterile fermenters, which renders the system highly susceptible to exogenous contamination by the feedstock harboring bacteria and fungi^28^,^29^. Although detrimental effects upon fermentation yield, feedstock-harbored microorganisms may also have a beneficial effect, either by interacting with the inoculated industrial yeasts or overcoming them due to superior fermentation performance^28^,^30^,^31^. Even though the interference of feedstock-harbored microorganisms is known for a long time, bacterial and yeast diversity of sugarcane stalks remain largely obscure. Knowledge of the composition of natural communities colonizing sugarcane organs is crucial to understanding its effect on biofuel production.

Here we conduct a comprehensive study of the microbiome structure, composition of mode of assemblage of the sugarcane organs during plant development. By sampling the roots, stalks and leaves of plants grown in native soil in a greenhouse and separately accessing their exophytic and endophytic compartments, we were able to describe the assemblage composition, distribution, and dynamics of naturally occurring microbial communities in a commercial sugarcane variety. Our study describes for the first time an inventory of bacterial and fungi communities associated with the most representative sugarcane plant organs. In addition to providing a model for the study of the sugarcane microbiota, we demonstrate the existence of highly abundant core microbiomes for both bacteria and fungi composed by a fraction of the entire diversity. These core microbiomes have organ patterns that do not significantly change in their composition throughout plant development. These findings indicate that the core microbiomes may have important roles in the plant biology.

## Results

### Assessment of sugarcane microbiome at the plant organ level

Naturally occurring bacterial and fungal communities were accessed in a commercial sugarcane variety grown on native soil in a greenhouse. Plants were cultivated for 3 annual harvest cycles without the addition of fertilizers. During the 4^th^ annual cycle, roots, stalks (bottom, medium and upper parts), top leaves and young shoots were sampled from plants 4, 6, 8 and 10 months after budding (Supplementary Fig. 1). Virgin soil (bulk soil) was also sampled from seven different locations within non-cultivated plots inside the greenhouse. For each plant organ, we accessed the bacterial and fungal communities associated to external (exophytic) and internal (endophytic) compartments. Microbial community composition was assessed through amplicon sequencing of 16S and ITS ribosomal RNA genes in the HiSeq 2500 platform (Supplementary Fig. 2). A total of 341 million reads, averaging 1.3 and 1.2 million reads per sample type, were generated for each 16S and ITS dataset, respectively (Supplementary Tables 1 and 2). Reads processing, OTU clustering and downstream analyses were conducted using an automated pipeline (Supplementary Fig. 3). The use of PNA clamps^32^ for sugarcane mitochondria and chloroplast ribosomal RNA genes increased the yield of bacterial and fungal reads; most of the organelle sequence amplification was suppressed during library preparation. This was particularly useful for endophytic samples that contained large amounts of mitochondria and chloroplast DNA (Supplementary Fig. 4). After the removal of low-quality reads and sugarcane ribosomal genes (Supplementary Figs 4 and 5), a total of 90.9 million reads from 16S and 86.8 million reads from ITS were clustered at ≥97% sequence identity using UPARSE^33^. After processing, a total of 23,811 bacterial and 11,727 fungal operational taxonomy units (OTUs) were recovered. The taxonomical assignment of OTUs was performed using the QIIME package^34^ after the removal of OTUs classified as chloroplasts, mitochondria or *Viridiplantae*. Technical reproducibility, assessed by calculating the Spearman correlation coefficient of OTUs with a relative abundance above a 0.0005% threshold, was over 0.90 (Supplementary Fig. 6). To access the microbial richness, we evaluated the number of observed OTUs, the Chao1 estimator and the Shannon index of all sample types. The bulk soil, rhizosphere, and endophytic root displayed the highest microbial richness, followed by young shoots with an intermediate number of OTUs. Meanwhile, for the stalks and leaf samples, the exophytic showed higher microbial richness than the endophytic compartment (Supplementary Fig. 7).

### Bacterial and fungal communities show distinct assemblage patterns among plant organs

**Figure 1:**
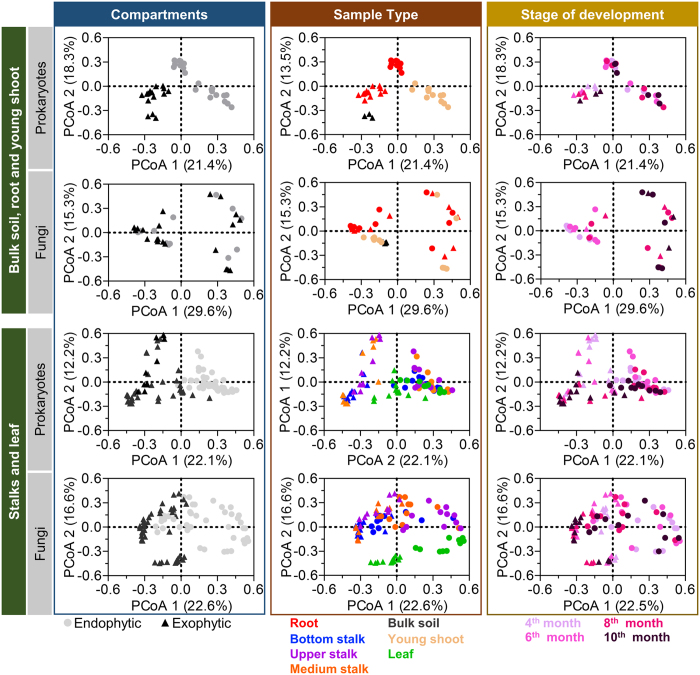
Main factors driving the microbiota composition of bacterial and fungal communities. The principal coordinate analyses (PCoA) of pairwise Bray-Curtis distance matrixes of filtered OTU tables rarefied to 18,000 reads. PCoA analyses were performed independently in two groups of samples, “root, bulk soil and young shoot” (belowground) and “stalks and leaves” (aboveground) for the bacterial and fungi datasets. For each group, the same graph was differentially colored to emphasize the influence of compartment, sample type and stage of development in the community assemblage. Compartment is the major driving factor for bacterial community assemblage for all samples. Fungal community assemblage is primarily driven by the sample type and compartment in aboveground samples and by the stage of development in belowground samples.

The bacterial and fungal community structure assemblages among sugarcane organs were compared using principal component analysis (PCoA) of Bray-Curtis dissimilarity matrixes on rarefied OTUs to identify the main drivers of microbial composition. For both bacterial and fungal communities, a clear distinction was observed between belowground organs (rhizosphere, endophytic root and bulk soil) and aboveground organs (stalks and leaves) samples (Supplementary Fig. 8a). Notably, young shoot samples grouped with bulk soil, rhizosphere and endophytic root to form a distinct cluster (ANOSIM values of R = 0.6599, P < 0.001 and R = 0.6304, P < 0.001, for bacteria and fungi, respectively). The belowground/aboveground patterns were particularly significant for bacterial microbiota, where the first principal component explained 21.4% of all differences (Supplementary Fig. 8a). Despite this common pattern, the bacterial and fungal community structures exhibited a distinct profile of sample similarity in aboveground plant organs, as shown by UPGMA hierarchical sample clustering (Supplementary Fig. 8b). We further dissected the variables contributing to the microbial distribution among plant tissues by looking at the effect of the tissue compartment, sample type and stage of development in the below and aboveground groups, separately. Because young shoots appeared to be closely related to belowground samples, we included them in the belowground group. The data indicate that the compartments (endophytic or exophytic) play the most significant role in defining bacterial diversity. Regardless of organ origin, exophytic and endophytic samples formed two distinct clusters in both groups of samples (Fig. 1, ANOSIM; R = 0.5446, P < 0.001 and R = 0.4017, P < 0.001 for bulk soil, root and young shoots versus stalk and leaf samples). However, a different pattern was found for the fungal community. Compartment had no significant role in fungal community assemblage for root or young shoots and had little influence on stalks and leaves (Fig. 1, ANOSIM; R = 0.2496, P < 0.001). In the group formed by belowground samples, sample type had the most significant influence on bacterial community assemblage (Fig. 1, ANOSIM; R = 0.5446, P < 0.001 and R = 0.3317, P < 0.001), but this effect was less pronounced for the fungal community assemblage. In contrast, aboveground fungal communities were strongly determined by sample type (Fig. 1, ANOSIM; R = 0.5592, P < 0.001). Plant developmental stages had only a minor effect on community assemblage for both bacterial and fungal communities, with the exception of belowground fungal samples, where the 4^th^- and 6^th^-month developmental stage samples were clustered apart from the 8^th^- and 10^th^-month samples. Taken together, the data indicate that compartment is the major driving variable for bacterial community assemblage, whereas fungal community assemblage is primarily influenced by sample type.

### Soil serves as reservoir for the assemblage of plant organ microbiota

**Figure 2:**
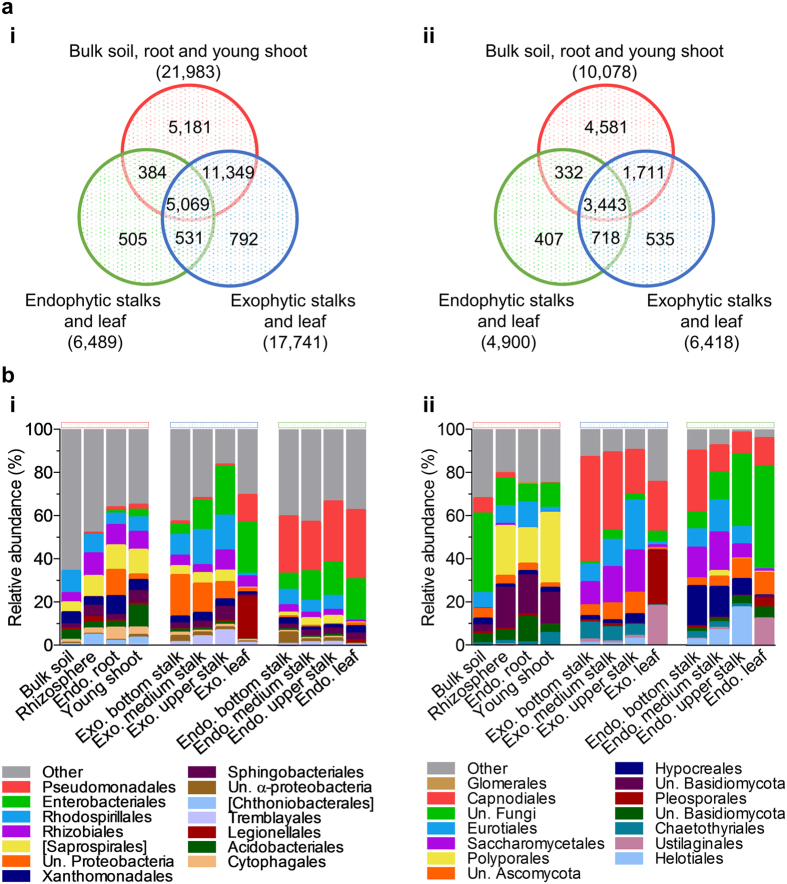
Soil serves as the main reservoir for bacterial and fungal groups that colonize sugarcane plant organs. (**a**) Number of shared OTUs among sample groups for bacteria (i) and fungi (ii). Samples were grouped according to their Bray-Curtis distance matrix similarity. Numbers in parenthesis indicate the total sum of OTUs in a given grouped sample. Numbers in overlapping regions indicate OTUs shared among grouped samples. (**b**) Taxon enrichment across sample types. Statistical significance was tested using Kruskal-Wallis, FDR-corrected P < 0.001 at the order level for bacterial (i) and fungal (ii) communities. Orders with an average relative abundance below 1% were grouped as “others”. Un.: Unkown; Exo.: Exo.; Endo.: Endo.

We further investigated whether the observed dissimilarities in bacterial and fungal community assemblages resulted from differences in microbial composition, abundance, or both. Given the large dataset, a MySQL database was set up for fast retrieval of OTU abundance and taxonomical information. We first asked to which extent different compartments and organs shared microbiota (Fig. 2). Remarkably, most bacterial and fungal OTUs associated with aboveground plant organs were also found in belowground and young shoot samples (Fig. 2a). In fact, only ~10% (1,828 out of 18,630; Fig. 2a) and 23% (1,660 out of 7,146; Fig. 2b) of the aboveground bacterial and fungal OTUs, respectively, were found exclusively in stalks or leaves. Although exophytic bacterial and fungal samples from stalks and leaves showed the highest richness in OTU count compared to their corresponding endophytic compartment, most of these OTUs were also present in belowground samples (Fig. 2a). The slightly less diverse endophytic samples from stalks and leaves also shared a large proportion of OTUs with either belowground or exophytic stalks and leaf samples (92% for both bacterial and fungal microbiota).

Because significant proportions of the microbial diversity were shared among samples, we focused on the differences in the relative abundance of taxa among samples. At the order level, 227 orders in bacteria and 74 in fungi showed significant differences between at least two samples (FDR-corrected Kruskal-Wallis test; P < 0.001; Supplementary Tables 3 and 4). Despite the large number of orders identified, it is noteworthy that only a minute fraction of them contributed to the total relative microbial abundance (Fig. 2b). The analysis also revealed that samples clustering together in PCoA analysis (Fig. 1) contained specific orders with similar relative abundance profiles (Fig. 2b). Notably, these orders were also found in bulk soil samples and were either enriched or depleted in samples across plant organs. For bacteria, a high relative abundance of *Rhizobiales*, *Saprospirales* and *Rhodospirillales* characterized the rhizosphere, endophytic root and young shoot samples, respectively (Fig. 2b(i)). *Enterobacteriales* were *ca*. 20-fold enriched in both exophytic and endophytic stalks and leaves compared to root, soil and young shoot samples. Meanwhile, *Pseudomonadales* and *Enterobacteriales* dominated endophytic stalks and leaves, respectively*. Rhodospirillales* was the third most abundant order in stalks, but it had minor abundance in leaves. In fungal communities, a large fraction of the diversity was classified as “unknown” at the order level (Fig. 2b(ii)). Among the identifiable orders, *Polyporales* differentiated roots, bulk soil and young shoots from the other sample types. *Capnodiales* were enriched in stalks and leaves, whereas *Saccharomycetales* were among the most abundant order for both exophytic and endophytic stalks.

**Figure 3:**
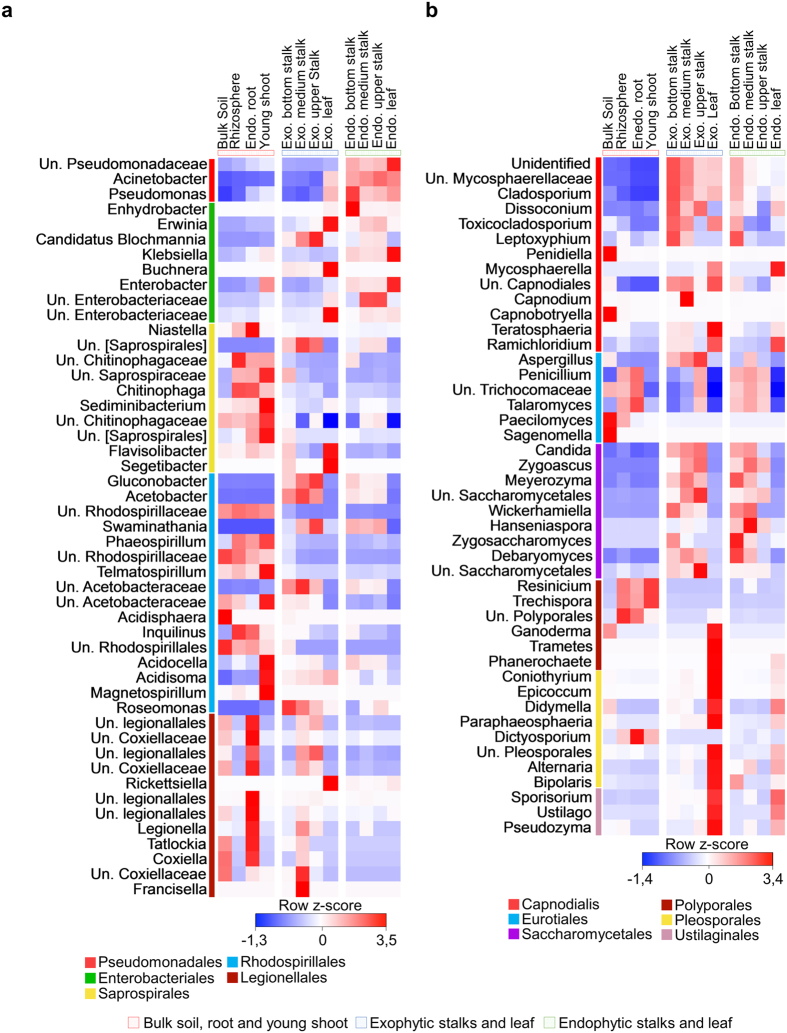
Heatmaps showing bacterial and fungal genera that significantly contribute to sample differentiation. (**a**) A total of 49 bacterial genera were significantly responsible (P < 0.001) for the distinct enrichment and depletion patterns found at the order level (Fig. 2b(i)). Among them, 20 genera were assigned as unidentified (Un.). (**b**) In the fungal community, a set of 45 genera significantly contributed to the enrichment and depletion pattern (P < 0.001), 8 of which were assigned as unidentified (Un.). Heatmaps were colored on the basis of row z-scores calculated on relative abundance. Exo.: Exophytic; Endo.: Endophytic.

Because just a few orders contributed strikingly to the differences in the relative abundance among different organs, we further investigated whether specific lower-level groups were responsible for the distinct enrichment and depletion patterns (Fig. 3). For bacteria, only 49 taxons at the genus level were responsible for the differences found within the *Pseudomonale*s, *Enterobacteriales, Saprospirales, Rhodospirillales* and *Legionellales* combined (Fig. 3). Among them, 20 could not be assigned to a specific genus and were classified as unknown. For fungi, 45 taxons distributed among *Capnodialis*, *Eurotiales*, *Saccharomycetales*, *Polyporales*, *Pleosporales* and *Ustilaginales* most contributed to the enrichment and depletion pattern (Fig. 3), among which most were unknown.

### Plant organs have core microbial communities represented by a small subset of highly abundant OTUs

**Figure 4:**
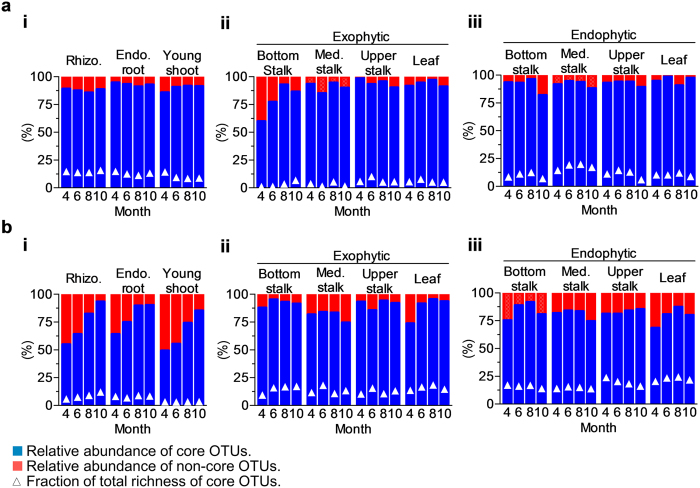
Relative abundance and richness of OTUs belonging to the core microbiome. Blue and red bars indicate the total relative abundance of core and non-core OTUs, respectively. Core OTUs richness is expressed as a fraction (%) of total OTU counts. (**a**) Bacterial core community (**b**) Fungi core community. Although there is a huge OTU richness for each sample, only a small fraction of OTUs belongs to the core microbiome. Inversely, this small fraction comprises most of the total relative abundance. Exo.: Exophytic; Endo.: Endophytic.; Med.: Medium.

The previous analyses suggest that although plant organs share some microbiota enrichment patterns, they keep a significant level of singularity by displaying distinct, highly abundant dominant genera. This finding, along with the fact that developmental stage plays a minor role in bacterial community assemblage, suggests the existence of stable cores represented by specific communities. We tested this hypothesis by searching for bacterial and fungal OTUs that consistently appeared across different plant samples and evaluating their relative abundance. A MySQL query was designed to look for OTUs that appeared in at least 90% of the sample types. We found that only a minor fraction of the total OTU richness fit this criterion for both bacteria (Fig. 4a) and fungi (Fig. 4b). Belowground samples and endophytic compartments for bacteria (Fig. 4a(i,iii)) and endophytic samples for fungi (Fig. 4b(iii)) had the highest fraction of OTU fitting the criterion representing, altogether, less than 25% of total OTU richness. For the exophytic bacterial communities (Fig. 4a(ii)), these OTUs accounted for less than 10% of total richness. In contrast, these small OTU fractions accounted for over 90% of the total relative abundance in all samples, for both bacterial and fungal communities. These OTUs consistently maintained their high relative abundance throughout plant development and were defined as belonging to the organ/compartment core microbiomes.

### Sample types have distinct profiles of preferential core OTU colonizers

**Figure 5:**
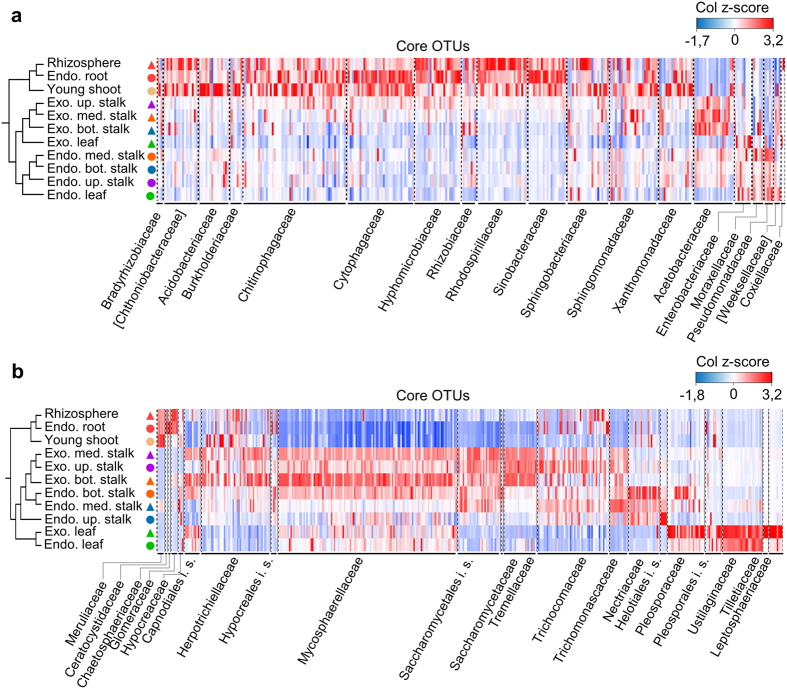
Sample types show a distinct pattern of core colonizers. The heatmap shows a distribution pattern of core OTUs across sample types based on relative abundance (column z-score). Samples were hierarchically grouped (group-average linkage) based on the pairwise Bray-Curtis similarity of the core OTU table. OTUs were organized by family-level classification. (**a**) Bacterial core OTUs. Belowground samples have similar colonizer profiles. Aboveground samples from the same compartment have similar colonizer profiles independent of the plant organ. (**b**) Fungal core OTUs. Samples from the same plant organ have similar profiles. Bot.: Bottom; Med.: Medium; Up.: Upper; Exo.: Exophytic; Endo.: Endophytic.

In view of their prevalence and high relative abundance, we examined in detail which bacterial and fungal groups were represented in the core microbiomes. We found that 322 core bacterial and 434 core fungal OTUs occupied the upper quartile of relative abundance distribution, representing over two thirds of the total relative abundance in all sample types. These OTUs were classified into 19 bacterial and 21 fungal families that preferentially colonized the different plant organs (Fig. 5a,b). Although sample types may share the same bacterial family as the preferred colonizer, they showed a distinct pattern of abundance within the OTUs classified in that given family (Fig. 5a). For example, although *Chitinophagaceae* and *Rhodospirillaceae* are dominant groups in rhizosphere, endophytic root and young shoot, a different profile of bacterial OTUs within these families prevails as the preferential colonizer in other sample types. Analogously, and similarly to what was found in bacterial samples, different sample types showed a unique profile of core fungal colonizers (Fig. 5b). As expected, rhizosphere, endophytic root and young shoot fungal communities showed similar profiles with respect to preferential family colonizers. However, fungal communities from aboveground samples showed a different pattern. The bottom, medium and upper stalks shared profiles more similar to each other, regardless of whether the compartment was exophytic or endophytic, compared to the leaves (Fig. 5b). Within the stalk, exophytic samples shared a similar profile that was distinct from that of the endophytic samples.

We next compared the taxonomical distribution and relative abundance of families containing core OTUs in bacteria (Supplementary Fig. 9) and fungi (Supplementary Fig. 10). Members of *Enterobacteriaceae*, *Moraxellaceae* and *Pseudomonadaceae* were found to be preferential colonizers of endophytic stalks and leaves (Supplementary Fig. 9, xix, xi, xii). Notably, *Moraxellaceae* represents 10–20% of the average relative abundance in these organ’s core OTUs (Supplementary Fig. 9, xi). *Acetobacteraceae* were highly abundant in endophytic stalks (6.6%, 5.1% and 6.1% relative abundance in the bottom, medium and upper stalks, respectively; Supplementary Fig. 9, i) but less abundant in leaves (0.3%). OTUs from this family were even more relevant in exophytic stalks, where they accounted for 8.4%, 15.1% and 16% of the total relative abundance in the bottom, medium and upper stalks, respectively. Rhizosphere, endophytic roots and young shoots harbored the highest OTU richness (Fig. 2a), but abundance was more spread over families. Nevertheless, OTUs classified as members of [*Chthoniobacteraceae*] (Supplementary Fig. 9, viii), *Hyphomicrobiaceae* (Supplementary Fig. 9, x), or *Rhodospirillaceae* (Supplementary Fig. 9, xiv) were more abundant in rhizosphere. *Hyphomicrobiaceae* (Supplementary Fig. 9, x), *Cytophagaceae* (Supplementary Fig. 9, vi) and *Sinobacteraceae* (Supplementary Fig. 9, xv) were more abundant in endophytic root, and *Acidobacteriaceae* (Supplementary Fig. 9, ii) were more abundant in young shoot. *Chitinophagaceae* presented the highest relative abundance and an even distribution within rhizosphere, endophytic root and young shoots (7.8%, 9.4% and 8.8%, respectively; Supplementary Fig. 9, v). Notably, a small set of bacterial families was not particularly favored in any specific set of samples but was abundant in all samples. Included in this set were *Burkholderiaceae* (Supplementary Fig. 9, iv), *Rhizobiaceae* (Supplementary Fig. 9, xiii), *Sphingobacteriaceae* (Supplementary Fig. 9, xvi), *Sphingomonadaceae* (Supplementary Fig. 9, xvii) and [*Weeksellaceae*] (Supplementary Fig. 9, xx) families.

Regarding fungi, core OTUs belonging to *Sistotremataceae*, *Meruliaceae*, *Ceratocystidaceae*, *Chaetosphaeriaceae* and *Glomeraceae* (Supplementary Fig. 10, xvii, ix, ii–iv) were more abundant in rhizosphere, endophytic root and young shoot samples. Meanwhile *Herpotrichiellaceae*, *Mycosphaerellaceae*, *Saccharomycetaceae*, *Tremellaceae*, *Trichocomaceae*, *Trichomonascaceae*, *Nectriaceae*, *Helotiales* i. s and *Saccharomycetales* i. s were broadly distributed and more abundant in both endophytic and exophytic stalk samples (Supplementary Fig. 10, vi, x, xiv, xix–xxi, xi, v, xv). Core OTUs classified as *Pleosporaceae*, *Pleosporales incertae sedis*, *Ustilaginaceae*, *Tilletiaceae*, *Leptosphaeriaceae* and Capnodiales *incertae sedis* were the most abundant groups in leaves (Supplementary Fig. 10, xii, xiii, xxii, xviii, viii, i).

### Most of the bacterial and fungal communities comprise previously unexplored diversity

Amongst the genera identified as belonging to the core microbiome communities, we searched for those that have members previously described as plant growth-promoting (PGP) or that have already been isolated from plants. Although some genera are fairly diverse with respect to richness and function, we asked to what extent the microbial communities associated with sugarcane and other plants had been already explored. Our survey showed that within the core microbiome, only 129 bacterial genera, out of 656 assigned to a known taxa, presented at least one isolated member described as a PGP. Among those, 51 have at least one member already isolated from sugarcane (Supplementary Table 5). As expected, members of the genera that have been extensively studied for their contribution for biological nitrogen fixation, such as *Azospirillum*, *Beijerinckia, Bradyrhizobium, Burkholderia, Herbaspirillum* and *Gluconacetobacter* were found in the rhizosphere and endophytic root (Supplementary Fig. 11, iv, vi, viii, ix, xix and xviii). However, rhizosphere and endophytic root samples were dominated by other highly abundant groups that have not been studied or have been poorly studied, and there is little or no information regarding their biological role in plant-microbe association (Supplementary Table 5). As an example of an unexplored highly abundant microbe, the genera assigned to the family *Chitinophagaceae* constituted 7.8% of total relative abundance in rhizosphere and 9.4% in endophytic root (Supplementary Fig. 9, v). A similar situation was observed for other families, such as *Cytophagaceae* and [*Chthoniobacteraceae*], which accounted for 1.7% and 3.3% of the total relative abundance in the rhizosphere and 4.4% and 1.2% in the endophytic root, respectively (Supplementary Fig. 9, vi and viii). We also found highly abundant groups such as *Hyphomicrobiaceae*, *Rhizobiaceae*, *Sphingomonadaceae*, *Rhodospirillaceae* (except *Azospirillum* and *Herbaspirillum*)*, Sphingobacteriaceae* and *Sinobacteraceae*, which are known to contain PGP members that have a high relative abundance in rhizosphere and endophytic root samples but have never been explored (Supplementary Fig. 9, x, xiii, xvii and xv). Groups such *Enterobacteriaceae*, *Pseudomonadaceae*, and *Moraxellaceae*, which together accounted for over 50% of total relative abundance in stalks, have been poorly studied regarding their biological role in these organs (Supplementary Fig. 9, xix, xii and xi).

Regarding fungal communities, most of the highly abundant groups identified in sugarcane organs have very few members whose presence and function have already been explored in plants such *Fusarium* and *Penicillium* (Supplementary Table 6 and Supplementary Fig. 12). Among the nonpathogenic fungi, few examples from the mycorrhiza group, such *Glomus*, are available (Supplementary Table 6 and Supplementary Fig. 12).

We found a remarkable richness of the yeasts *Candida*, *Debaryomyces*, *Hanseniaspora*, *Meyerozyma*, *Wickerhamiella*, and *Zygosaccharomyces* within the stalk microbiome. However, only *Candida* and *Wickerhamiella* belong to the core microbiome of this organ. Members of the *Candida* genus (assigned to *Saccharomycetales incertae sedis*) accounted for up to 9.4% of the relative abundance in stalks (Supplementary Fig. 12).

**Figure 6:**
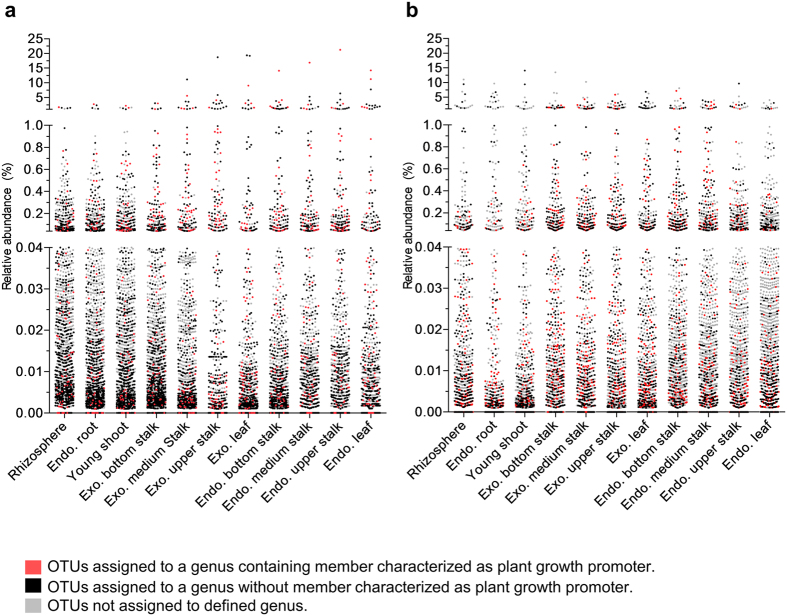
Untapped diversity in fungal and bacterial communities. Dots represent the relative abundance of core OTUs in a given sample type. Each assigned genus was searched in the literature for isolated members with growth-promoting traits (Supplementary Tables 5 and 6). The vast majority of core OTUs have no evidence of functional whole in association with plants. (**a**) Core bacterial OTUs. (**b**) Core fungal OTUs. Exo.: Exophytic; Endo.: Endophytic.

Finally, we investigated the relative abundance of OTUs classified within a genus that had at least one representative member already isolated from plants compared to the overall diversity. Our data show that over 75% the bacterial diversity identified in this study have never been explored (Fig. 6). This discrepancy is even higher in the fungal community, where almost none of the most abundant genera have ever been described in any plant species.

## Discussion

Microbes associated with sugarcane have been studied in the past by direct bacterial isolation from roots using defined culture media. Although this strategy has led to the isolation of potentially relevant bacteria, particularly for the understanding of plant-associated nitrogen fixation^11^, it has limited the studies to a small number of isolates. In this work, we used community analysis based on 16S and ITS profiling to explore the composition and dynamics of naturally occurring bacterial and fungal communities associated to the roots, stalks and leaves of sugarcane plants. The study took into account the diversity, organ distribution, abundance and potential importance to sugarcane biology, aiming to provide a comprehensive description of the microbiota associated to sugarcane plants grown in a native soil.

Microbiome origin, dynamics and assemblage patterns are all important for the elucidation of its possible role in plant growth, development, and response to biotic and abiotic stress. Despite the increasing interest in plant microbiomes, most studies have been limited to soil and roots samples. In addition, they have focused solely on bacteria, and little is known about the diversity and abundance of fungi communities associated to plants. Here we expanded the concept of plant microbiome by accessing bacterial and fungal communities in the exophytic and endophytic compartments of the roots, young shoots, stalks and leaves of sugarcane plants. The data support the notion that organ compartment, either endophytic or exophytic, plays a key role in determining the structure and composition of naturally occurring bacterial communities, irrespective the organ type. In contrast, the compartment plays a minor role in the assemblage of the fungal communities, whereas sample type strongly affects community structure. The plant’s developmental stage has been shown to affect the rhizosphere and the endophytic root bacterial community in potato^35^, although it has no significant effect on the assemblage of *Arabidopsis* root bacterial communities^7^. Our data show that in sugarcane the plant developmental stages have little effect on the bacterial community assemblage but have a significant effect on the fungal community assemblage in roots.

Soil is considered the main source of bacterial root colonizers^7^,^36^. However, bacterial communities associated with grapevine flowers and leaves have also been suggested to originate from soil^37^. Questions underlying the origin of microbial communities are of biological importance to sugarcane because the crop is produced by successive stalk harvesting from initial stalk-cutting plantations^38^. Annually, stalks are harvested for the production of sugar and ethanol, whereas ratoons are left in soil and will produce next plant generation. In this context, the stalk could be a major carrier for microbial communities. Although we did not trace microbial succession across years, we were able to demonstrate that soil communities prevail as the main source of plant bacterial and fungal colonizers. Over 90% of all OTUs present in roots, stalks and leaves were also present in the bulk soil samples. Thus, the data suggest that the microbial diversity present in the bulk soil colonizes the plant organs at early stages of plant development, and the organ-specific richness and abundance may be dictated by functional or adaptive plant-microbe interaction.

Although a significant fraction of the members of microbial communities are shared among plant organs, OTU richness decreased as we moved from belowground samples to the exophytic and to endophytic compartments of aboveground plant organs. Consequently, differences among organs reflect the difference in the relative abundance of shared OTUs. Different proportions of bacterial and fungal groups are enriched or depleted in aboveground plant organs compared to the bulk soil. Notably, the young shoots budding formed from the underground ratoon had bacterial and fungal communities very similar to those of roots. This finding suggests that the diverse bacterial and fungal communities from the soil all invade the tissues of young plantlets and that members of microbial communities are soon enriched or depleted in an organ-and compartment-specific manner. This process may take place at the very early stages of plant development; the different organs of the 4-month-old plant already presented enriched communities that remained relatively constant towards plant maturity.

Our data show that there are distinct core communities of bacteria and fungi associated with each plant organ. These core microbiomes comprise a small subset of the total OTU richness that accounts for over 90% of the total relative abundance in any given organ. The concept of core community was introduced in human microbiome studies to define a relatively stable community contributing to important biological functions^39^,^40^. Among sugarcane organs core microbiomes there are bacterial and fungal families that are preferential colonizers and the distribution of OTUs within these families differs among organ types and compartments. The most parsimonious assumption is that the core microbiome comprises microorganism with relevance to plant growth and health. Otherwise, these groups would not be maintained at high abundance along plant development. Surprisingly, our data reveals that most microbial groups studied so far in association with sugarcane represents a minor portion of the total core microbiome. These results open the field for exploring an untapped diversity with potential to benefit plant growth and development.

Within core microbiomes, we identified bacterial genera containing plant growth-promoting members. For some of them, at least one representative has been isolated and studied regarding its potential as a plant-growth promoter. Among them, we found free-living diazotrophs, such as *Azospirillum*, *Bacillus*, *Beijerinckia*, *Bradyrhizobium*, *Erwinia*, *Enterobacter*, *Herbaspirillum* and *Gluconoacetobacter*, which may potentially contribute to nutrient acquisition. Surprisingly, however, the groups to which these bacteria belong represent minor components of the core microbiome, whereas many of the most abundant bacterial groups in core microbiomes have never been isolated or studied. These data suggest that the most abundant groups in core microbiomes from the different sugarcane organs may contain microbes with relevant biological functions for plant growth performance other and more diverse than that reported for nitrogen-fixing bacteria associated with sugarcane^19^,^20^,^21^,^22^. In addition, our data give perspective to the difficulty in demonstrating the beneficial effect of plant inoculation with nitrogen-fixing bacteria isolated from sugarcane if they are indeed not abundant enough in plant tissues^41^.

A rich and diverse fungal component of the sugarcane microbiome was also uncovered in this work. These data are particularly significant because there is very little information on the potential benefits of plant-fungi associations. Previous studies have primarily focused on the beneficial association of mycorrhizal with roots to improve nutrient acquisition^42^. Compared to the bacterial component of the microbiome, the fraction of the fungal diversity that has received attention so far is negligible, and our data suggest that there is a large open field to study the possible role of fungal colonizers in plant growth, development and response to biotic and abiotic stress.

Microbial communities associated to sugarcane stalks are relevant for industrial fermentation process for ethanol production. Based on the results shown in this work it can be assumed that the sugarcane juice used as feedstock for yeast ethanol fermentation harbors an enormous bacterial and fungal diversity. Reports evaluating the dynamics of yeast population through chromosome fingerprinting of yeast samples taken from industrial fermentation vessels have repeatedly shown that commercial strains are replaced by either *Saccharomyces* or non-*Saccharomyces* indigenous yeast during fermentation^28^,^30^. In light of our results, this phenomenon is not surprising; yeasts genera represented by *Candida*, *Debaryomyces*, *Hanseniaspora*, *Meyerozyma*, *Wickerhamiella* and *Zygosaccharomyces* accounted for up to 11.9% of total fungal diversity in the stalks. Candida accounted for the largest fraction of the total relative abundance in the stalks fungal community. Few studies have evaluated the microbial community during fermentative process^43^ and none have taken into consideration the origin of these communities and its impact on health and yield of fermentative process. As our results show that the diversity of yeast and bacteria in stalks is far unknown than considered before, we believe it should be taken in consideration in prospects of microbial impact on biofuel production from sugarcane feedstock.

Microbiome studies have shown that unknown or untargeted diversity is a valuable biotechnological resource. Although studies have focused on specific microbial groups, these studies have not been able to explain or quantify their contribution to biological processes such as biological nitrogen fixation. Our data suggest that huge, untapped diversity may be responsible for these limitations, imposing levels of complexity that cannot be explained by targeting single microbial groups. Finally, we believe that high sugar accumulation in the sugarcane stalks may be a unique biological model for prospecting microbial diversity for biotechnological application.

## Material and Methods

### Plant material and experimental design

Sugarcane plants of the SP80-3280 commercial variety were provided by the bioenergy company Cosan (cosan.com.br). Stalks of field-grown mature plants were harvested at Usina Santa Helena, Fazenda Santo Antônio (GPS coordinates −22.735657, −47.305069) in November of 2009 and planted in a greenhouse at Center for Molecular Biology and Genetic Engineering (CBMEG - http://www.cbmeg.unicamp.br), State University of Campinas (Unicamp). The plants were grown for three harvesting cycles without the addition of fertilizers and under a controlled watering regime. The harvesting cycles were similar to the commercial plantations practice: the stalks were cut annually, and the ratoon kept for producing a new crop generation. The samples were collected from plants at the fourth cycle.

Samples were taken from the roots, stalks, leaves and young shoots of 3 independent clumps containing 3–4 well-developed stalks. The stalks were sampled at the bottom, medium and upper regions. To address whether there were variances in microbial community along development, tissues were collected from plants 4, 6, 8 and 10 months after shoot sprouting (Supplementary Fig. 1). To gain better insight into the microbial origin and dynamics, virgin soil samples were collected from seven plots where no sugarcane has been grown.

We accessed two compartments for each sample: (1) the exophytic, defined here as the microbial community attached to the external regions of the plant organs, and (2) the endophytic, the microbes associated to the inner parts of the plant organs. For the young shoot, only the endophytic community was analyzed.

### Sampling of rhizosphere, endophytic root and bulk soil microbiomes

Approximately 1 m^2^ and 0.8 m deep were excavated around the plant clumps to removed the entire plant from the soil. Once removed, the aerial parts of the plant (stalks and leaves) were separated from the root system. Large soil aggregates were removed by manually shaking the roots. Pruning shears were used to cut the roots from the remaining portion of the stalks. Root pieces were transferred to a sterile plastic tray containing 1.5 L of sterile ice-cold PBS-tween20 solution (7 mM Na_2_HPO_4_, 3 mM NaH_2_PO_4_, pH 7.0 and 0.05% tween20) and extensively washed by hand. The washing solution was filtered through 4 layers of sterile bandage and centrifuged at 200× g for 5 min at 4 °C to remove large debris. The supernatant was centrifuged at 3,000× g for 15 min at 4 °C, and the resulting pellet, defined as the sample containing the rhizosphere-enriched microbial communities, was frozen in liquid nitrogen and stored at −80 °C.

The PBS-tween20-washed roots were subjected to a second washing step in tap water. Washed roots were transferred to plastic bags containing in ice-cold, sterile MilliQ water and subjected to two cycles of 10 minutes of sonication. After sonication, the excess water were removed and 100 g of roots were chopped and blended in ice-cold PBS (7 mM Na_2_HPO_4_, 3 mM NaH_2_PO_4_, pH 7.0). The blended buffer was filtered through 4 layers of sterile bandage and centrifuged at 200× g for 5 min at 4 °C to remove particulates and cell debris. The supernatant was then centrifuged at 3,000× g for 15 min at 4 °C, and the pellet, defined as the sample containing endophytic root enriched microbial communities, was frozen in liquid nitrogen and stored at −80 °C.

### Sampling of exophytic and endophytic microbiome of bottom, medium and upper stalks

Exophytic and endophytic stalk samples were taken from pools of three stalks from the same clump. Depending on the developmental stage, a clump could have stalks with different numbers of internodes. Stalks selected for sampling had the same number of internodes and were among the most developed in the clump. Upon plant removal from soil, the top 3–4 leaves were detached from the stalks, placed in sterile plastic bags and stored on ice for further processing. The stalks were divided into bottom, medium and upper parts, and each of them was processed independently. The stalk cuts were washed in ice-cold PBS-tween20 solution using a sterilized brush. The washing solution was filtered through 4 layers of sterile bandage and centrifuged at 200× g for 5 min at 4 °C. The supernatant was centrifuged at 3,000× g for 15 min at 4 °C, and the pellet, defined as the sample containing exophytic stalk microbial communities, was frozen in liquid nitrogen and stored at −80 °C.

The washed stalks were chopped into small pieces and total of 100 g of the chopped stalks were blended in ice-cold PBS-tween20. The blended solution was filtered through 4 layers of sterile bandage and centrifuged the same way as the exophytic samples. The pellets, defined as the sample containing endophytic stalks, was frozen in liquid nitrogen and stored at −80 °C.

### Sampling the exophytic and endophytic leaf microbiome

Sugarcane plants have one attached leaf per stalks internode. We used the leaves +1 to +4 from the three sampled stalks to prepare the leaf exophytic and endophytic microbiota. The microbiota were sampled by washing the complete extension of leaves in sterile ice-cold PBS-tween20. The washing solution was filtered through 4 layers of sterile bandage and centrifuged at 200× g for 5 min at 4 °C to remove debris. The supernatant was centrifuged at 3,000× g for 15 min at 4 °C, and the pellet, defined as the sample containing exophytic leaves-enriched microbial communities, was frozen in liquid nitrogen and stored at −80 °C. The washed leaves were washed again in tap water and chopped into small pieces. A total of 100 g of mixed chopped leaves were blended in ice-cold PBS solution. The blending solution was filtered through 4 layers of sterile bandage and centrifuged at 200× g for 5 min at 4 °C to remove debris. The supernatant was centrifuged at 3,000× g for 15 min at 4 °C, and the pellet, defined as the sample containing endophytic leaves-enriched microbial communities, was frozen in liquid nitrogen and stored at −80 °C.

### Sampling the young shoot microbiota

Two young shoots for each plant clump were detached from the stalk-root zone, and their first external leaf layers were removed. The remaining tissues were chopped into small pieces, and 100 g of tissue was blended in ice-cold PBS and processed in the same way as endophytic leaves.

### DNA extraction and quantification

DNA extraction was performed using the MoBio PowerSoil DNA Isolation kit (MoBio, 12888), which has been adopted for microbial surveys^44^. The frozen microbiota pellets were defrosted and suspended in the beading buffer from the MoBio PowerSoil kit. The DNA was isolated according to the manufacturer’s protocol with minor modifications. A heating step at 65 °C for 10 minutes was included after adding the C1 solution, and two-step ethanol clean-up were used. The total DNA yield varied from one sample to another, but for all samples, the heating step improved the DNA yield. DNA was quantified by Qubiit fluorometric quantification assay and stored at −80 °C.

### Primer design

To access the bacterial and archaea communities, the V4 region of the 16S ribosomal gene was targeted using the 515f and 806r primers^45^ with modified overhangs (Supplementary data). The fungal community diversity and abundance was accessed using modified ITS9 and ITS4 primers to target the ITS2 region^32^ (Supplementary data). Sequencing libraries were prepared using two PCR steps. The first step (PCR 1) was designed to amplify the targeted ribosomal gene regions and to add the Illumina Nextera transposase sequence to the amplicon. The second step (PCR 2) used primers from the Illumina Index Kit PCR primers to add a dual index system of rows and columns that enabled multiplexing up to 96 samples. Both forward and reverse primers for PCR 1 were amended with frame shift (FS) sequences in its 5′ overhang to improve sequence diversity and overall read quality. For each targeted ribosomal gene region, three types of forward primers for PCR 1 were designed, containing 6 (FS1), 5 (FS2) and 4 (FS3) random nucleotide bases in their FS sequence. Equimolar ratios of forward primers were pooled in a mix for use at PCR 1. The reverse primers contained 6 random nucleotides in the FS sequence (Supplementary Fig. 1). All oligonucleotide primers were purchased from Sigma using HPLC purification and adjusted to 10 μM for use.

### Library preparation and sequencing

PCR 1 was performed in 20 μL reactions using the Advantage 2 PCR kit (Clontech, 639206). PNA clamp sequences^32^ were included to minimize the amplification of mitochondria and chloroplast 16S sequences. For 16S libraries, the reaction contained 30 ng DNA, primers at final concentrations of 0.75 μM each and PNA for chloroplast and mitochondrial clamps at 0.75 μM each. The same condition was used for the ITS libraries, except that PNA clamps were not included. The DNA for each sample was independently amplified in triplicate reactions. For 16S PCR 1 amplification, the thermocycler program was set at initial denaturing at 95 °C for 5 min, followed by 25 cycles of denaturing at 95 °C for 30 s, PNA annealing at 78 °C for 10 s, primmer annealing at 50 °C for 60 s and extension at 68 °C for 60 s. The same thermocycler program was used for ITS PCR 1 amplification, but the step for PNA annealing was omitted. Amplicon replicates were pooled, purified using Agencourt AMPure XP beads (Beckman Coulter) at a bead-to-DNA ratio of 0.7:1, resuspended in 30 μL of MilliQ water and evaluated in agarose gels.

The PCR 2 reaction followed the same protocol for both 16S and ITS PCR 1 products. Each cleaned PCR 1 product within the same sample received a unique combination of forward and reverse primers (respectively, N7 and S5 Illumina dual index oligos). The choices of primers were made considering Illumina recommendations for a dual multiplexing system. The reactions included 13 μL of KAPA HiFi HotStart ReadyMix, 3 μL of Illumina N7xx oligo, 3 μL of Illumina S5xx oligo and water for a final volume of 25 μL. The PCR 2 program was set at initial denaturation at 95 °C for 5 min, followed by 12 cycles of denaturation at 98 °C for 20 s, annealing at 55 °C for 30 s and extension for 30 s. PCR 2 products were purified using Agencourt beads (0.7:1) and resuspended in 30 μL of MilliQ water.

Sequencing libraries were quantified using the qPCR-based quantification kit from Kapa (KK4835, Kapa Biosystems), diluted to 2 nM. The sequencing libraries were denatured by mixing 5 μL of the 2 nM pooled libraries with 5 μL of 0.2 N fresh NaOH and incubated for 5 min at room temperature. Following denaturation, 980 μl of chilled Illumina HT1 buffer was added to adjust the library at 20 pM. A final dilution step was taken to adjust libraries 16 pM by mixing 800 μL of 20 pM library with 200 μL of chilled HT1 buffer. The library concentration of 16 pM with a 5% spike of PhiX Illumina Control Library achieved an average of 850–950 K/mm^2^ clusters in the HiSeq 2500 Illumina sequencer (data not shown). Libraries were clustered in a cBot according to Illumina protocol and sequenced in HiSeq 2500 in rapid mode. The sequencer was custom set to produce 250pb per paired-end reads.

### Sequence demultiplexing

The demultiplexing of sequencing reads was performed with CASAVA (Consensus Assessment of Sequence and Variation) software (Illumina, v.1.8.2), allowing no mismatches for barcodes. Because more than one run was performed, some samples had the same barcode sequence. Because downstream analysis requires unique barcodes, .fastq files were altered using a custom Perl script to add unique bases to the barcode sequences and make them unique for each sample.

### Automated pipeline for read processing

A Perl script was developed to automate the sequence analysis steps after demultiplexing (Supplementary Fig. 3). The upstream sequence processing, including the merging of pair-end sequences, quality filtering, the removal of sugarcane sequences and OTU clustering, was automated in a pipeline based on UPARSE^33^. The parameters were tested and adjusted independently for the 16S and ITS datasets. All samples from a given targeted ribosomal gene region were pooled in a single file, processed and clustered together to enable comparison between different tissues and developmental stages.

The downstream processing, including taxonomical classification and alpha/beta diversity assignment, was done using QIIME^34^. OTU sequences, OTU tables correlating the number of observations for a given OTU in a given sample and taxonomical information for each OTU using different classification software and databases was organized in a MySQL relational database.

### Data processing and OTU clustering of 16S amplicons

Paired-end reads were merged using “fastq_mergepairs” from the USEARCH (http://www.drive5.com/) collection of commands with parameters “-fastq_maxdiffs 10 -fastq_truncqual 4”. Reads that did not merge were discarded. Several quality filtering methods were tested, and the one yielding optimal read retention with an acceptable quality was the maximum expected error-filtering method implemented in USEARCH through the command “fastq_filter” (http://www.drive5.com/). Highly stringent quality parameters were used, and reads with more than 0.5 expected errors were discarded. In addition, reads were trimmed to retain only the primer sequences in its ends. Sugarcane mitochondrial and chloroplast 16S sequences were filtered using DUK software with parameters “-k 20 -c 2”. Reference plastidial and mitochondrial 16S sequences were obtained from a rough assemblage of sugarcane genomes of an in-house shotgun dataset. Maize 16S mitochondrial and chloroplast sequences served as drivers in a blastn search for sugarcane orthologous. An automated pipeline following the description of the original UPARSE pipeline performed the subsequent steps of dereplicating, discarding singletons and clustering.

### Data processing and OTU clustering for ITS amplicons

The ITS targeted ribosomal gene region is longer^46^ than the 16S V4 sequences. An initial analysis indicated that many paired-end reads from ITS libraries did not overlap. This result is a indication that read length might not be enough to cover the entire ITS region of some microbial groups. In order to avoid bias, we decided to use only the first read of the paired-end sequences to perform the ITS amplicon analysis. Single reads were tail trimmed to 175 bp and filtered for a 0.5 expected error threshold using the “fastq_filter” from the USEARCH command. Sugarcane ITS sequences were trimmed using DUK software with parameters “-k 20 -c 2” and a reference sequence obtained from an in-house sugarcane reference assemblage. Reads with no recognizable primer sequences were discarded (primer filter) and the remaining reads were tail primed to the primer sequence (tail trimming). The proceeding steps of dereplicating, discarding singletons and clustering was done using the UPARSE pipeline.

### Technical reproducibility

The sequencing reproducibility was accessed using 12 rhizosphere samples. The DNA from each sample was used to prepare and sequence two independent libraries. The technical replicates went through the sequence analysis pipeline together and were rarefied to a common depth. The relative abundance of each OTU was log10 transformed and plotted against the log10-transformed relative abundance of that same OTU in the other technical replicate. The initial measure of Pearson correlation for OTUs’ relative abundance in each technical replicate averaged 0.6. The low correlation was primarily due to a minor portion of OTU with a low relative abundance. We defined the technical reproducibility threshold as OTU with a relative abundance above 0.0005%. Filtering OTU with this threshold resulted in a correlation above 0.9 in all cases.

### OTU taxonomical assignment and OTU table construction

OTU sequences were assigned a taxonomy using the RDP classifier in QIIME 1.6 with a confidence threshold of 0.5 against the Greengenes^47^ database (May 2013 release) and the UNITE database^48^ for 16S and ITS sequences, respectively. Taxon names were kept the same described in the databases. The OTU table was generated from the USEARCH file output through the script “uc2otutab.py” (http://drive5.com/python/uc2otutab_py.html) and converted into biom format with taxonomical metadata for use in QIIME. All OTUs annotated as chloroplasts, mitochondria and Viridiplantae were removed from the OTU table. OTUs with a relative abundance below the technical reproducibility threshold in a given sample were discarded. The final table was defined as the “filtered OTU table” and used for diversity metric calculations. The “filtered OTU Table” was rarefied to 18,000 reads using QIIME and was defined as the “Rarefied OTU Table”. Both tables and taxonomical assignments were inputted in a MySQL database.

### Diversity metrics

To determine whether there were differences in the community richness among plant organs, we evaluated sample types using the chaos1 estimator and the Shannon diversity index. These metrics were generated using a modified pipeline to retain singletons. Calculations were performed using the QIIME package in samples rarefied to 2 MM reads. Samples with reads less than 2 MM were rarefied to the maximum number of reads.

### Main drivers of bacterial and fungal communities

OTU tables for bacterial and fungal communities were retrieved from the rarefied OTU table in MySQL and converted to biom format. The Bray-Curtis dissimilarity matrix was calculated for both datasets and used for PCoA and ANOSIM analysis using QIIME. The graphs were visualized in GraphPad PRISM v 6.0 (GraphPad Software, Inc.; www.graphpad.com).

### Differentially enriched or depleted OTUs

The differential relative abundance of each OTU among samples was detected with a Kruskal-Wallis test with multiple correlation testing (FDR) independently for the bacterial and fungal datasets. The test was applied for the order and genus levels using QIIME. In both cases, a threshold oh P < 0.001 was used to determine whether an OTU was differentially enriched in a given organ.

### Defining OTU core communities

Core microbial community was defined as formed by the OTU that consistently appeared in at least 90% of plant samples following the criterion established eslewere^49^,^50^,^51^. The search was performed per sample (combination of sample type and compartment, Supplementary Fig. 1) using a MySQL query over the filtered OTU table to look for OTU fitting the criterion. MySQL query were also used to evaluate relative abundance of each OTU in different sample types. The taxonomical histograms retrieved from MySQL were plotted using GraphPad PRISM v 6.0 (GraphPad Software, Inc.; www.graphpad.com).

## Additional Information

**Accession code**: The raw sequence data are available in Sequence Read Archive (SRA) under the accession SRP076731.

**How to cite this article**: Souza, R. S. C. *et al*. Unlocking the bacterial and fungal communities assemblages of sugarcane microbiome. *Sci. Rep*. **6**, 28774; doi: 10.1038/srep28774 (2016).

## Supplementary Material

Supplementary Information
