# Supplementary material for: Unlocking the bacterial and fungal communities assemblages of sugarcane microbiome
Source: Sci Rep. 2016 Jun 30;6:28774. doi: 10.1038/srep28774 (PMC4928081; doi:10.1038/srep28774)
Supplement: Supplementary Information [file srep28774-s1.pdf]

Unlocking the bacterial and fungal communities assemblages of sugarcane microbiome

Rafael Soares Correa de Souza[1], Vagner Katsumi Okura[1], Jaderson Silveira Leite Armanhi[1], Beatriz Jorrín[2], Núria Lozano[2], Márcio José da Silva[1], Manuel González-Guerrero[2], Laura Migliorini de Araújo[1], Natália Ferreira Verza[1], Homayoun C. Bagheri[3], Juan Imperial[2,4] and Paulo Arruda[1,5,*]

[1]Centro de Biologia Molecular e Engenharia Genética, Universidade Estadual de Campinas (UNICAMP), 13083-875, Campinas, SP, Brazil

[2]Centro de Biotecnología y Genómica de Plantas (CBGP). Universidad Politécnica de Madrid (UPM), 28223 Pozuelo de Alarcón, Madrid, Spain

[3]New Energies Division, Repsol Technology Center, 28935 Mostoles-Madrid, Spain

[4] Consejo Superior de Investigaciones Científicas, Madrid, Spain

[5]Departamento de Genética e Evolução, Instituto de Biologia, Universidade Estadual de Campinas (UNICAMP), 13083-875, Campinas, SP, Brazil

Supplementary Figures

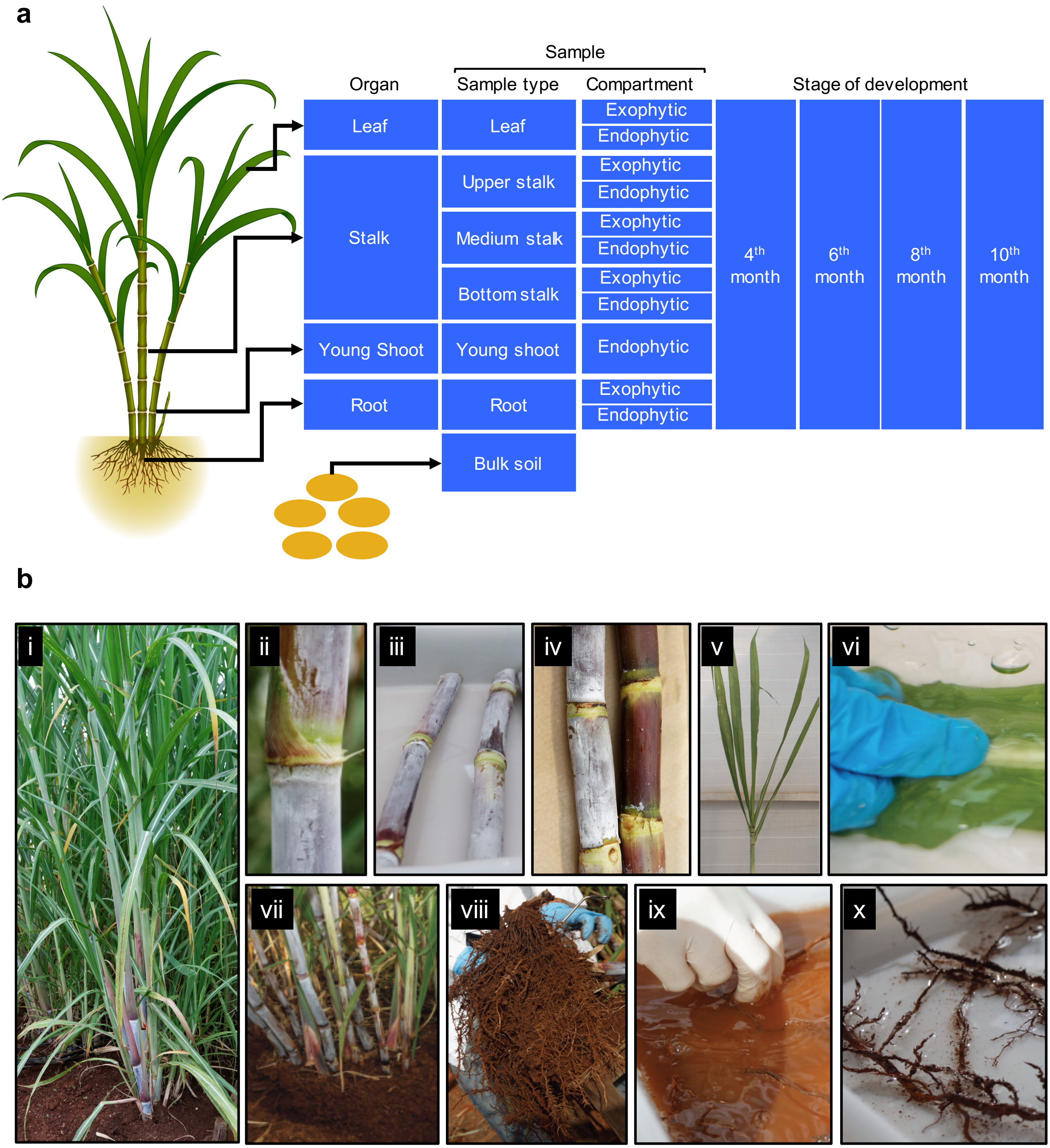

Supplementary Figure 1 - Experimental design and sampling plan overview. (a) Roots, young shoot, stalks and leaf of commercial sugarcane variety SP80-3280 had their microbial community sampled. Stalk has been portioned in bottom, medium and upper stalk. Bulk soil has also been collected from a area where no sugarcane plant has been planted summarizing 7 different sample types. Sugarcane tissues were accessed in two different compartments, endophytic and exophytic, with the except of young shoot from which only endophytic sample was collected. Each combination of sample-type and compartment, here defined as sample, where collected from sugarcane plants in its third annual cycle after 4, 6, 8 and 10 month of shoot germination. **(b)** Harvesting photos. (i) Sugarcane ratoon at 4th month after budding; (ii and iii) Stalks detail; (iv) Stalk pieces before (left) and after (right) microbiome sampling procedure; (v) Sampled leafs. (vi) Detail of leaf during sampling procedure. (vii) Sugarcane ratoon being excavated. (viii) Detachment of root from soil. (ix and x) Roots during sampling procedure.

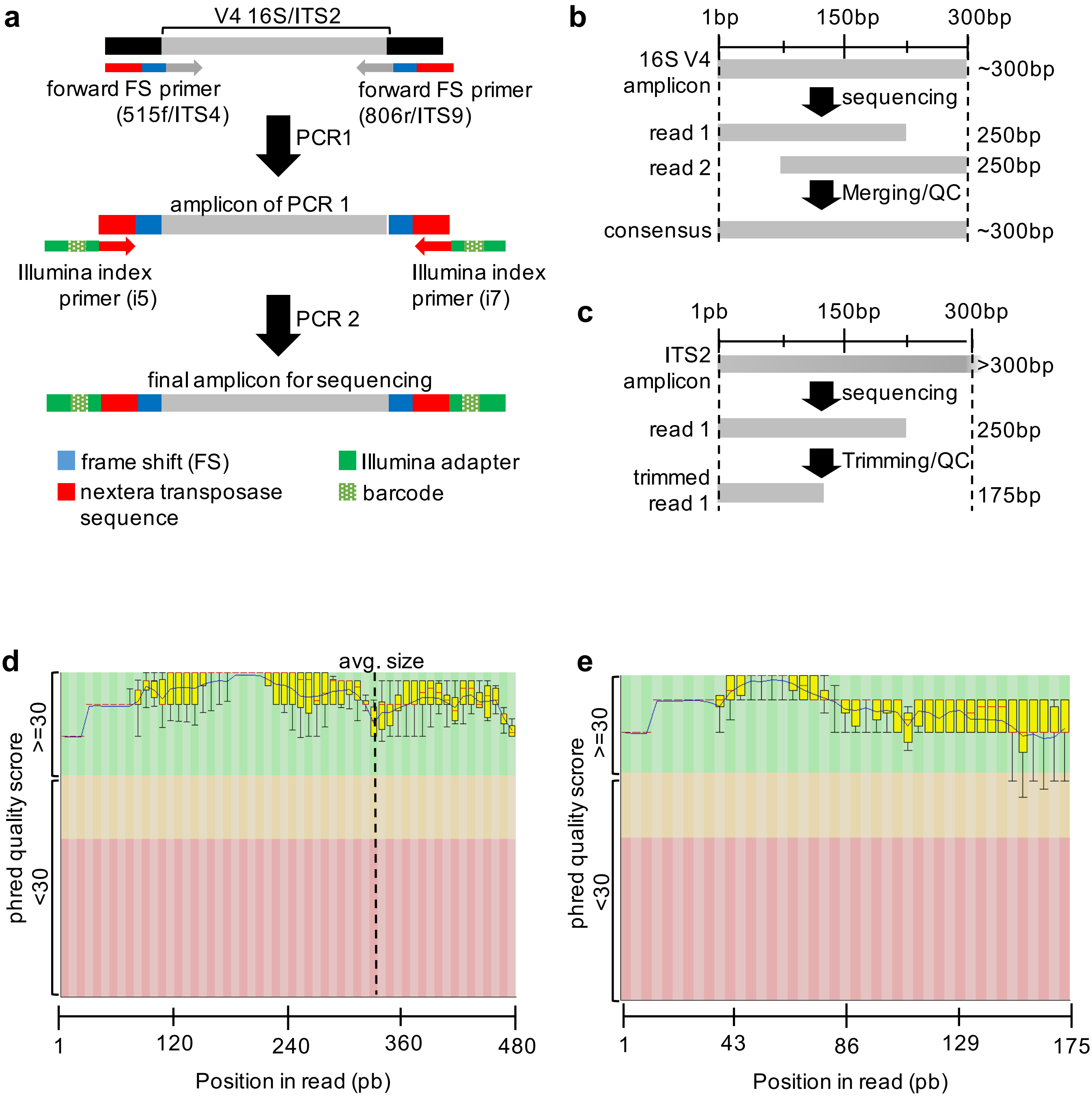

Supplementary Figure 2 - Sequencing library preparation schema, amplicon processing and read quality. (a) The V4 region of 16S and ITS2 were amplified and tagged with barcodes and Illumina adapters through a two-step PCR. The first step of PCR (PCR 1) amplifies target gene regions and adds frame shift sequence (FS) amended with the Nextera transposase sequence. The second step of PCR (PCR 2) uses the Illumina index primer to amplify the PCR 1 product and to add Illumina adapters with a dual index barcode system. **(b)** Amplicon sequencing and read processing for V4 16S amplicons. Amplicons were sequenced with HiSeq 2500 with paired-end reads of 250 bp. The reads were merged to a consensus. The paired-end reads of 16S libraries were merged to consensus. **(c)** Amplicon sequencing and read processing for ITS 2 amplicons. Read 1 was trimmed to a fixed length of 175bp. **(d)** The quality score per base along the 16S consensus after reads merging to a consensus and quality filtering. **(e)** Quality score per base along trimmed read of ITS amplicons and quality filtering. pb.: base pair; avg.: average.

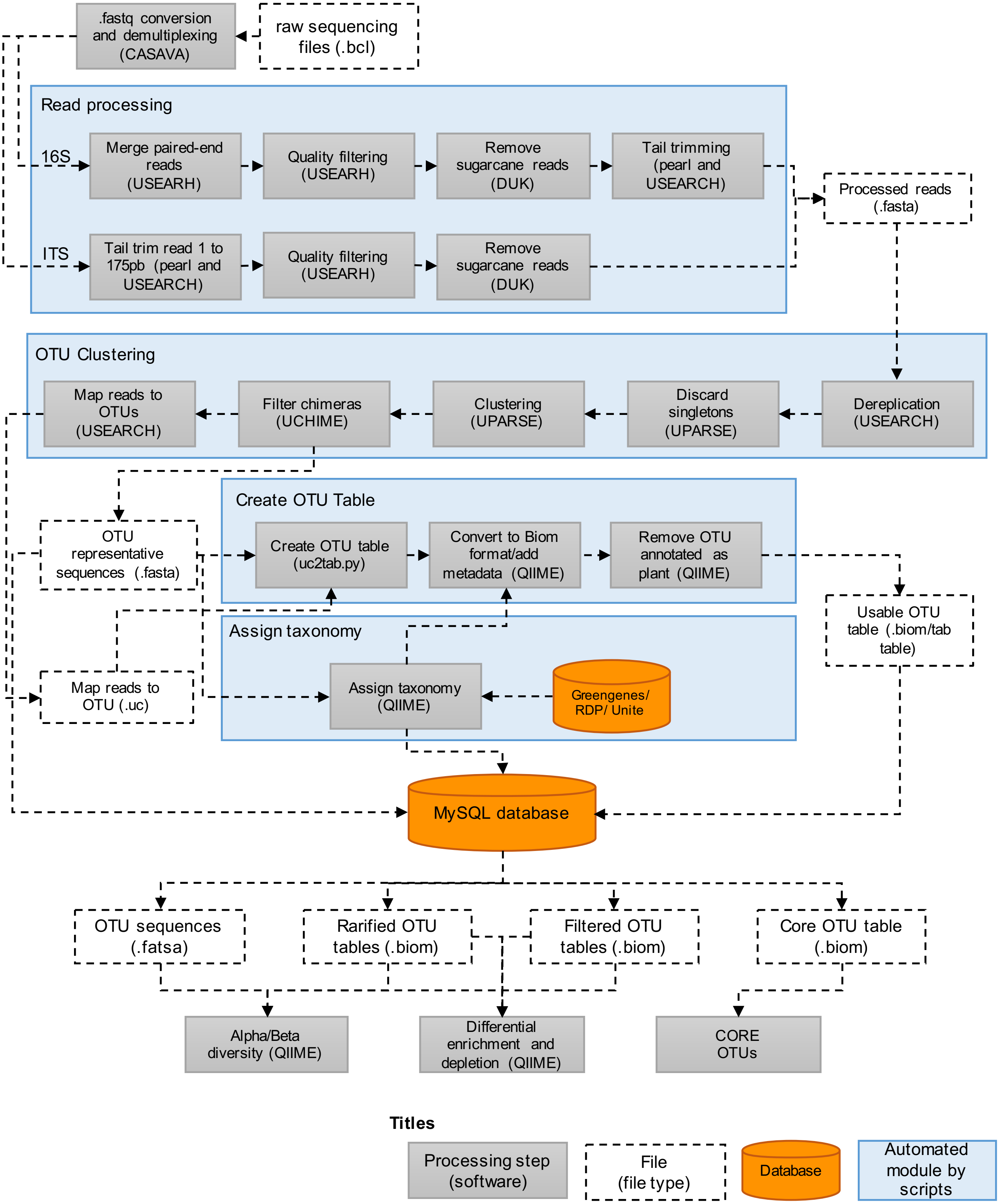

Supplemental Figure 3 - Schematic representation of the bioinformatic pipeline for data processing and analysis of 16S and ITS amplicons. Raw data from the HiSeq equipment was demultiplexed and inputted into an automated pipeline for read processing, OTU clustering, OTU table creation and taxonomy assignment. Information was stored in a MySQL database and retrieved for downstream analysis, such as alpha and beta diversity, community enrichment evaluation and core OTUs discovery.

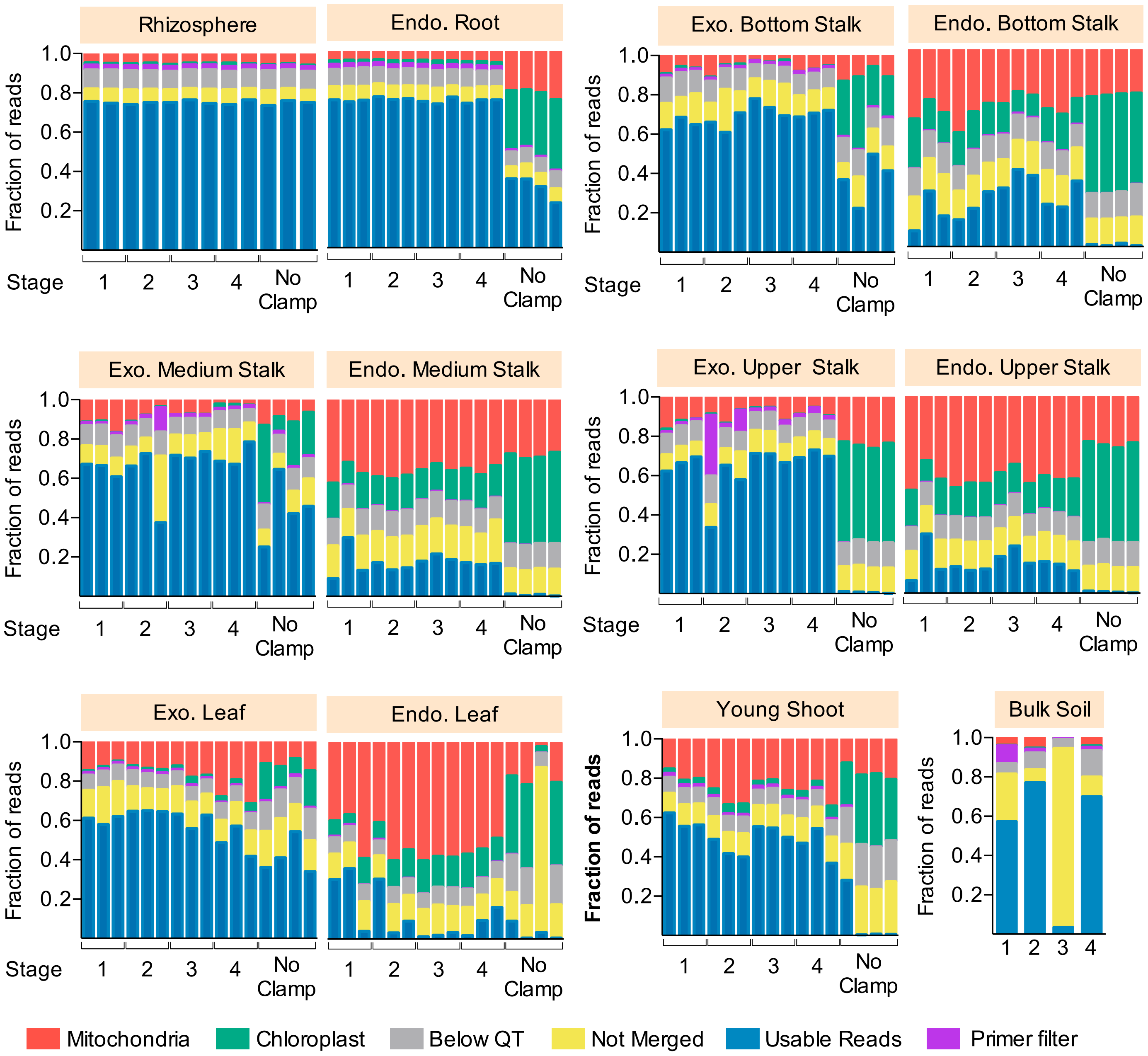

Supplementary Figure 4 - Sequence statistics of 16S libraries after read processing. Each sample was quality filtered, paired-end merged, filtered by chloroplast and mitochondrial sequences, and tail trimmed by the start of the primer sequences. For each sample type, we included 4 control libraries in which no clamp for mitochondria and chloroplast sequences were used to evaluate its efficiency.

.

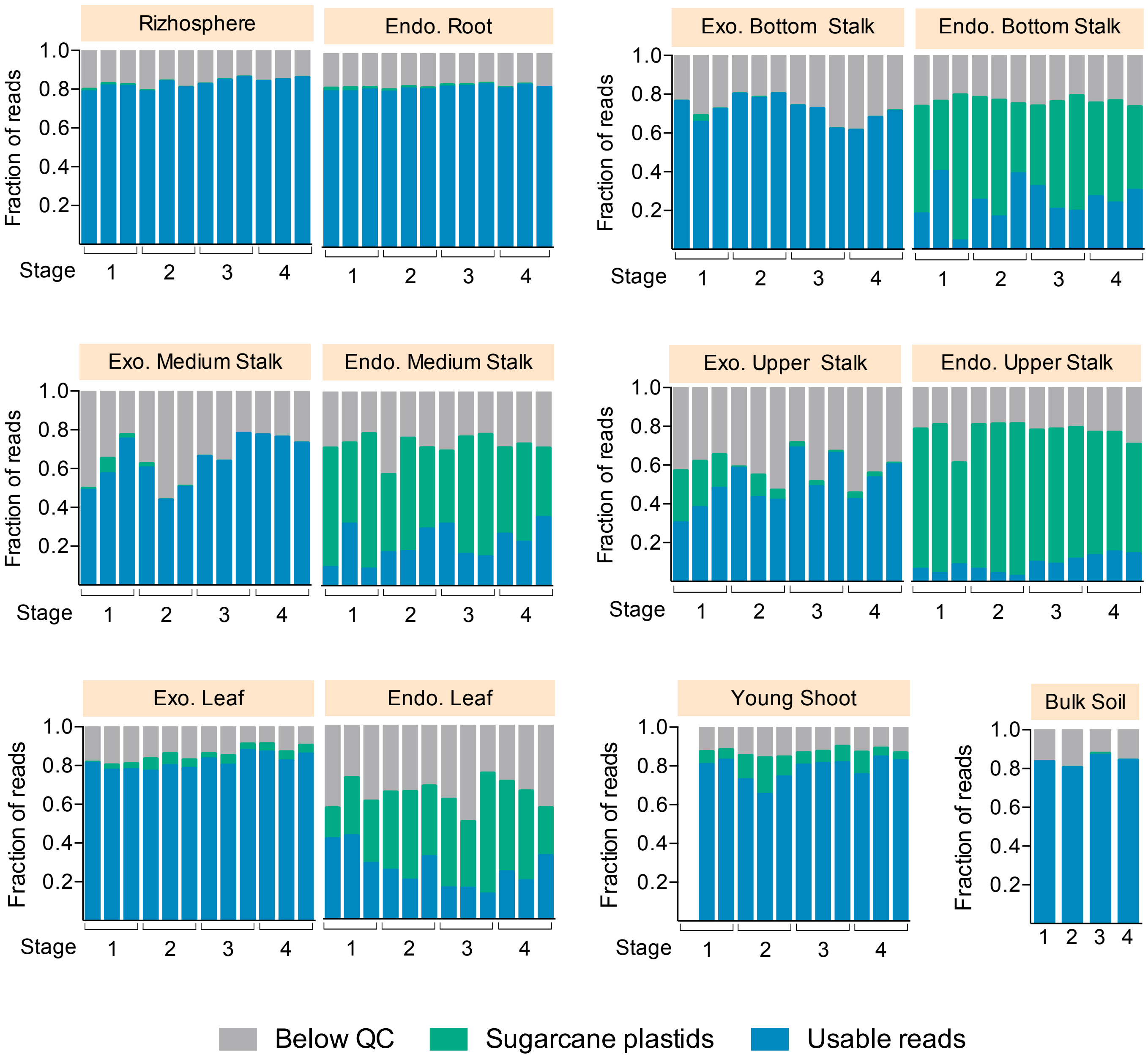

Supplementary Figure 5 - Sequencing statistics of ITS libraries after read processing. The reads of each sample were quality filtered and tail trimmed to a fixed position of 175 pb. Sugarcane ITS sequences were removed, resulting in usable reads that were inputted into a clustering pipeline.

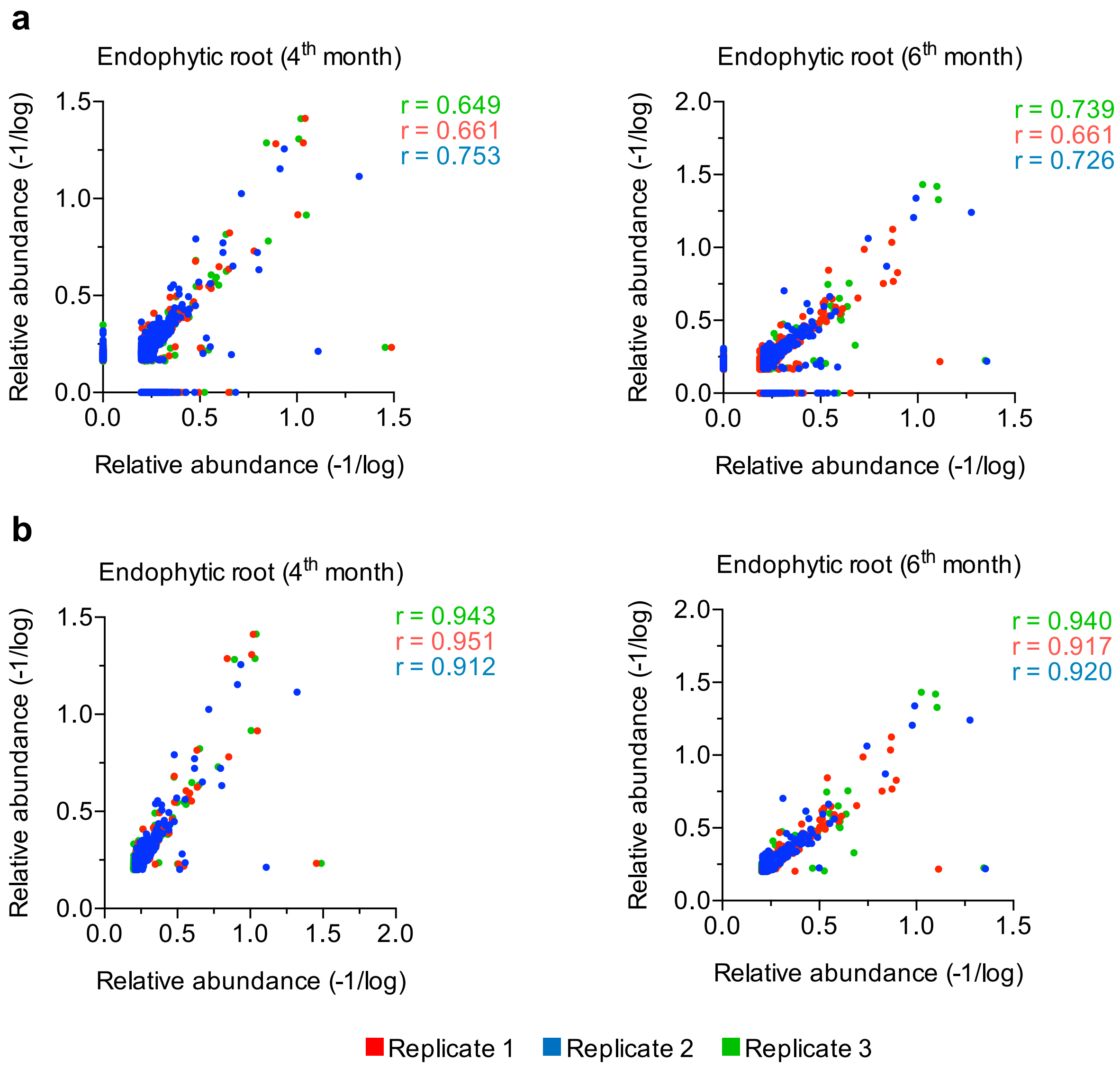

Supplementary Figure 6 - Technical reproducibility of library preparation and sequencing. Reproducibility was assessed using 6 different samples of endophytic root microbiota: 3 samples from the 4th month after shoot germination (replicates 1, 2 and 3) and 3 from the 6th month after shoot germination. The same DNA samples were used for library preparation and sequencing twice independently (technical replicates). Each OTU's relative abundance was -1/log transformed and plotted against its technical replicate (x-axis *VS.* y-axis). The Pearson correlation (r) was calculated for each pair of technical replicates. **(a)** No abundance filter was applied. **(b)** An abundance filter was applied (only an OTU with a relative abundance of >0.0005).

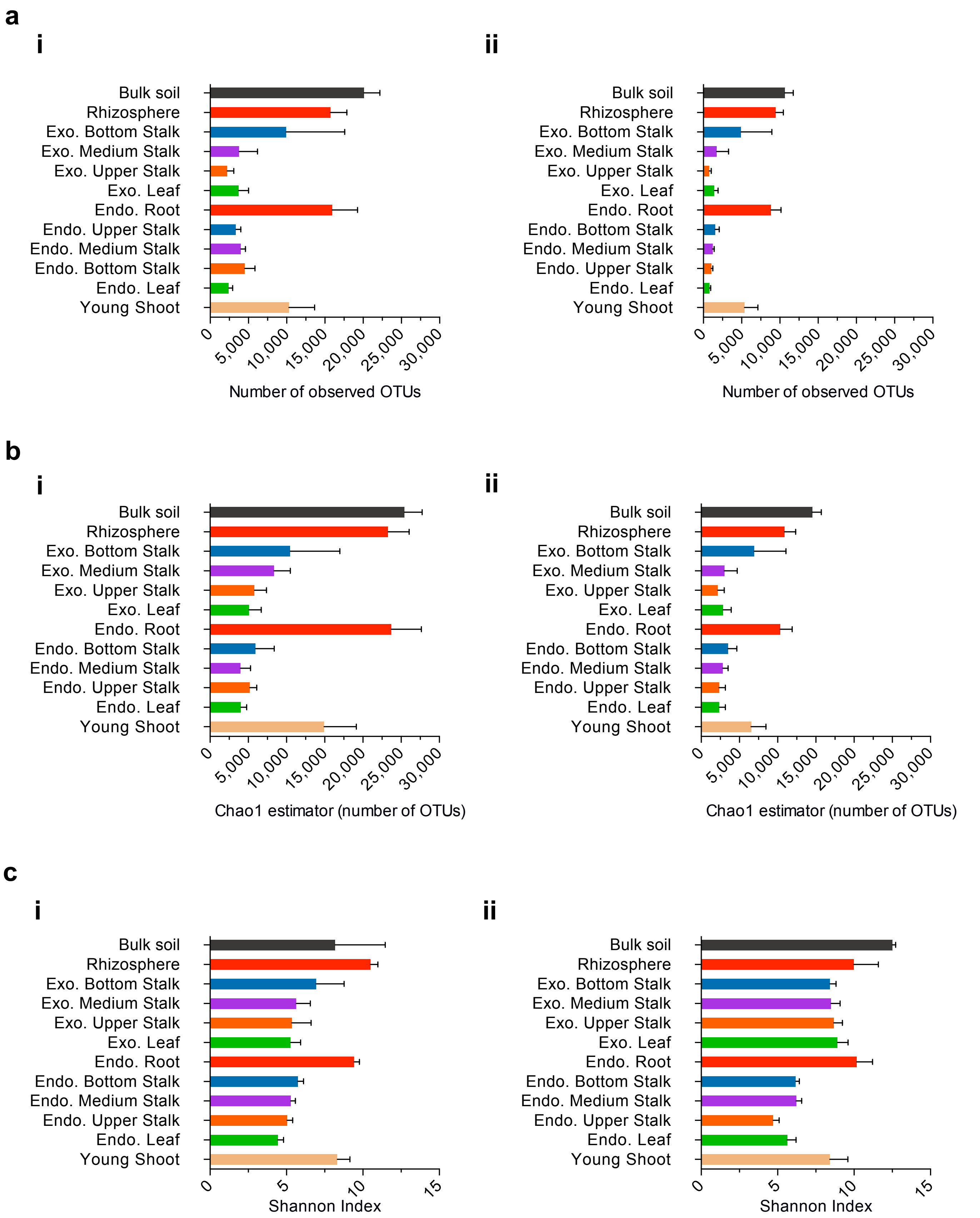

Supplementary Figure 7 - Diversity metrics. (a) Number of observed OTUs for (i) bacterial and (ii) fungal communities. OTUs were counted on a usable OTU table using MySQL query. **(b)** Chaos1 estimator for (i) bacterial and (ii) fungal communities. **(c)** Shannon index diversity for (i) bacterial and (ii) fungal communities. Samples were rarefied to 2 MM reads. (Samples with reads less than 2 MM rarefied to the maximum number of reads.) Exo.: Exophytic; Endo.: Endophytic.

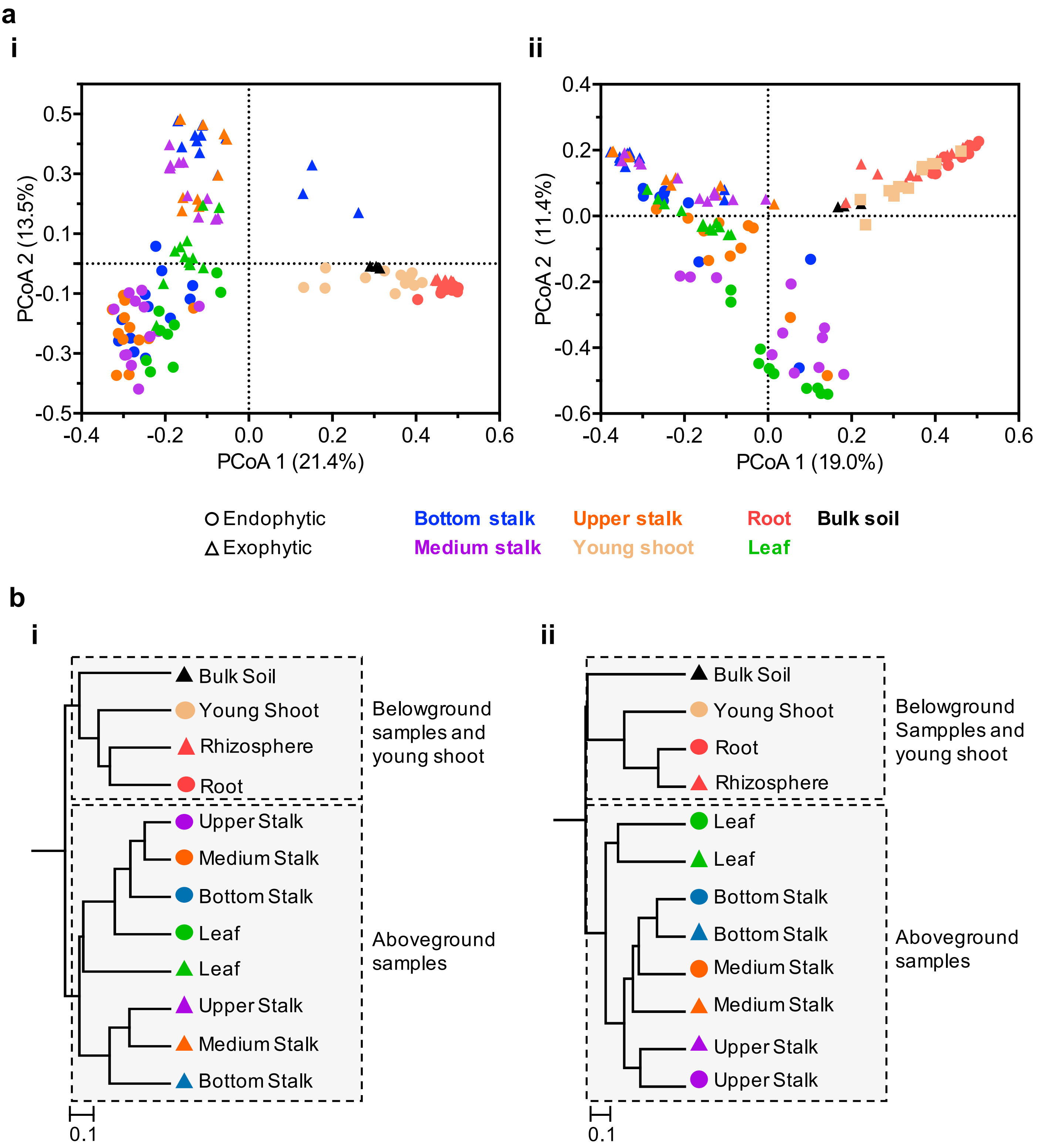

Supplementary Figure 8 - Aboveground and belowground samples showed differences in their compositions and structure in bacterial and fungal communities. (a) Principal coordinate analysis (PCoA) of pairwise, usable, Bray-Curtis distance matrix of the usable rarified OTU table for the **(i)** bacterial and **(ii)** fungal datasets. **(b)** Bray-Curtis dissimilarity of sample types for **(i)** prokaryotic and **(ii)** fungal datasets. The filtered OTU table was rarefied to 18,000 counts per sample and grouped hierarchically by sample type (UPGMA method) to highlight the relationships among sample types.

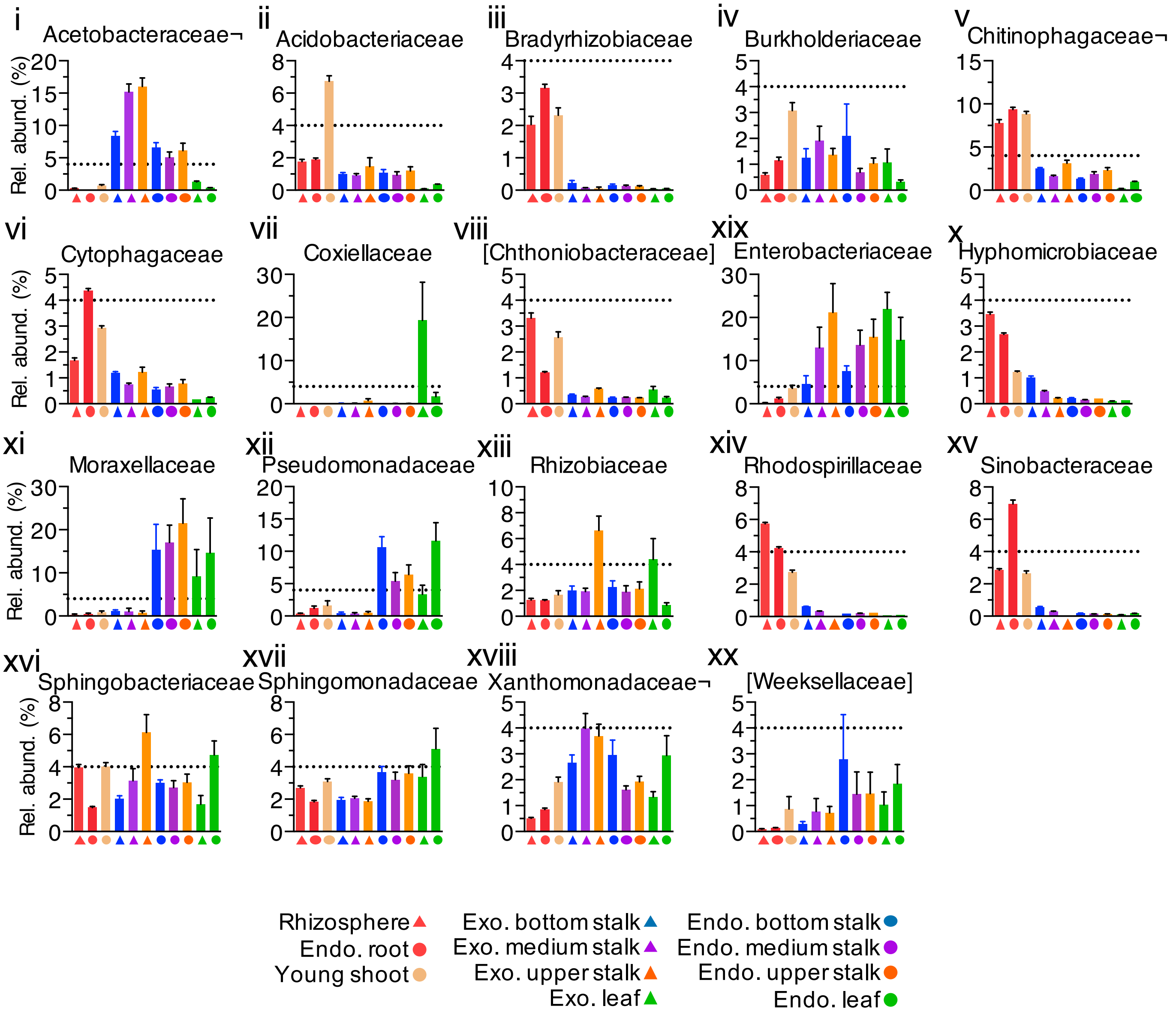

Supplementary Figure 9 - Relative abundance of relevant bacterial families in the core microbiome. Relative abundance was calculated as the sum of the relative abundance of core OTUs assigned to a given bacterial family. The horizontal line highlights the same relative abundance value in all graphs for comparative purposes. Exo.: Exophytic; Endo.: Endophytic.

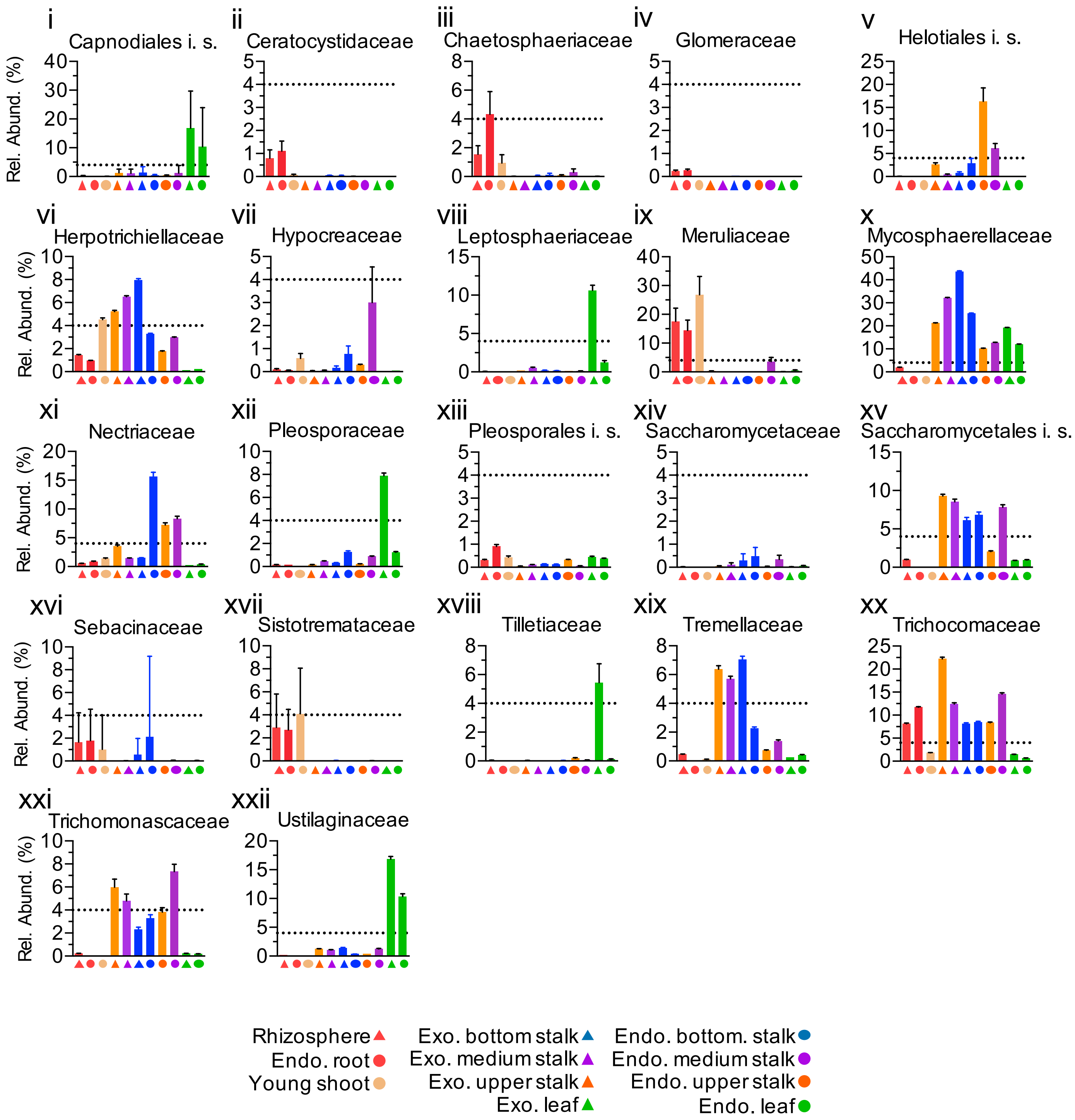

Supplementary Figure 10 - Relative abundance of relevant fungal families in the core microbiome. Relative abundance was calculated as the sum of the relative abundance of the core OTU assigned to a given fungal group. The horizontal line highlights the same relative abundance value in all graphs for comparative purposes. Exo.: Exophytic; Endo.: Endophytic.

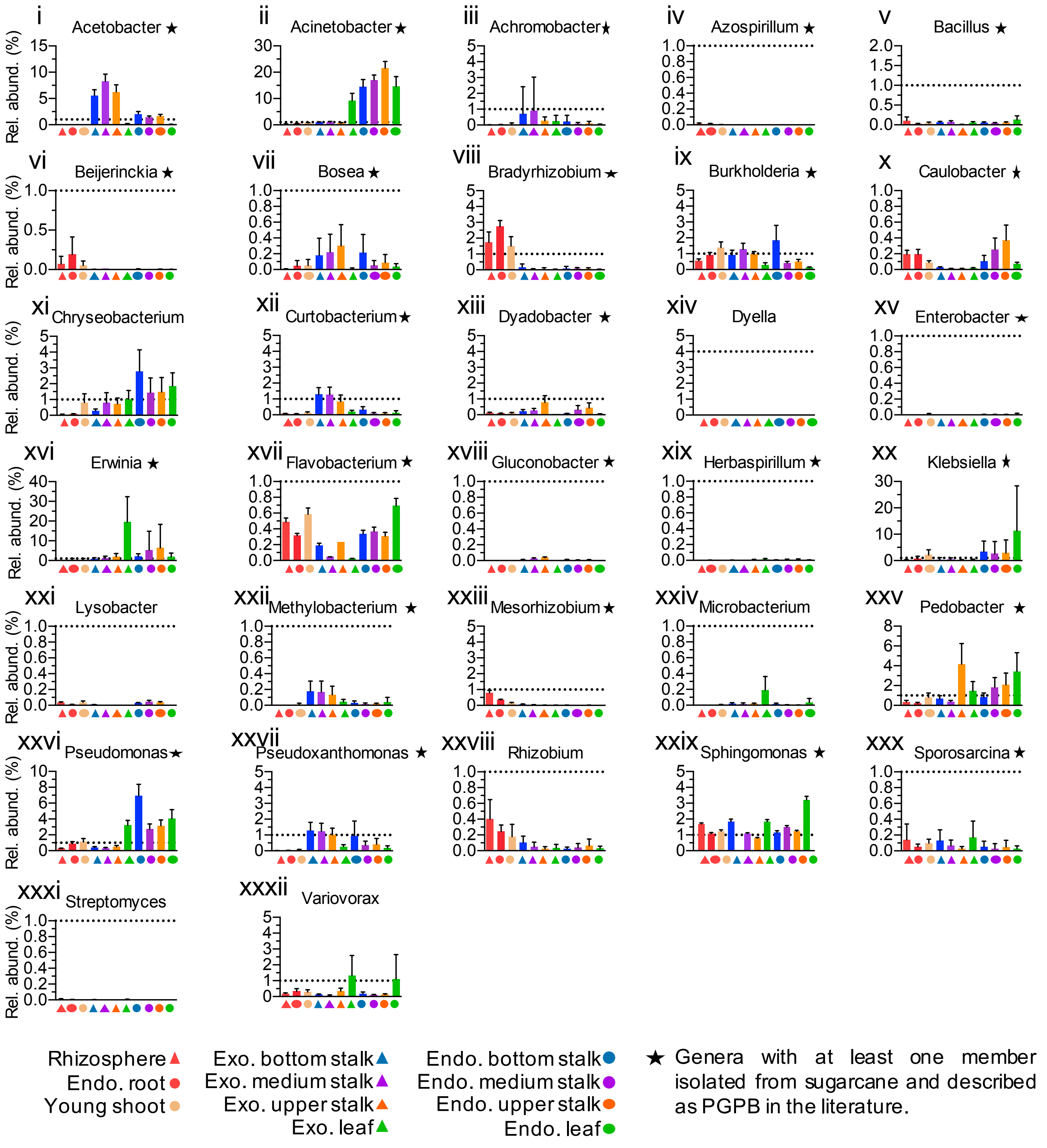

Supplementary Figure 11 - Relative abundance of bacteria genera for which at least one member is isolated and described as plant growth-promoting bacteria (PGPB) in the literature. Literature mining was performed for each bacterial genus looking for representatives with described plant growth-promoting traits. Genera with at least one member isolated from sugarcane and described as growth promoting in the literature are highlighted. The horizontal line highlights the equivalent value for relative abundance in all graphs for comparative purposes. Exo.: Exophytic; Endo.: Endophytic.

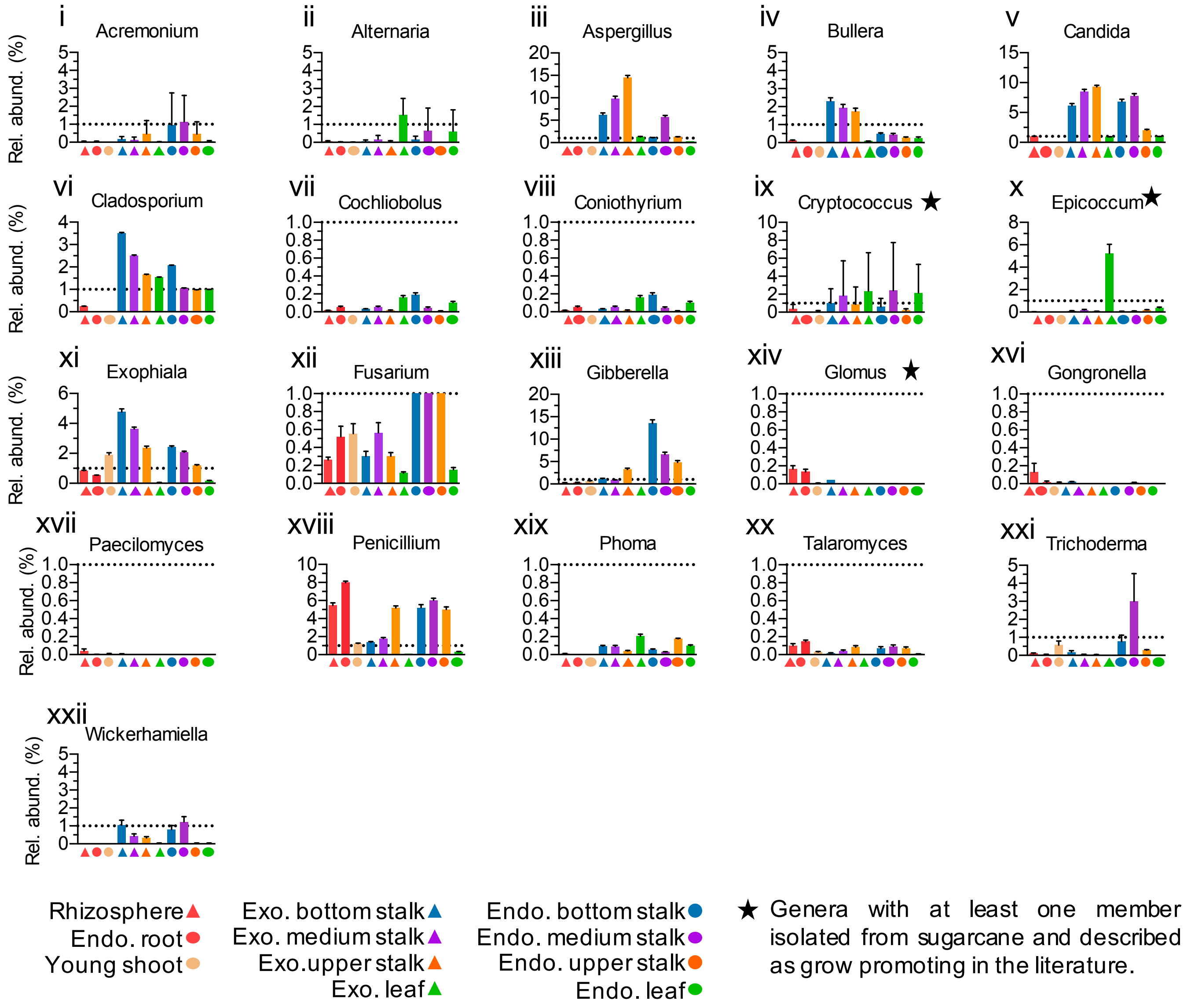

Supplementary Figure 12 - Relative abundance of fungi genera for which at least one member is isolated and described as plant growth promoting (PGP) in the literature. Literature mining was performed for each fungal genus looking for representatives with the described plant growth-promoting traits. Genera for which at least one member is isolated from sugarcane and described as growth promoting in the literature are highlighted. The horizontal line highlights the equivalent value for relative abundance in all graphs for comparative purposes. Exo.: Exophytic; Endo.: Endophytic.

Unlocking the bacterial and fungal communities assemblages of sugarcane microbiome

Rafael Soares Correa de Souza[1], Vagner Katsumi Okura[1], Jaderson Silveira Leite Armanhi[1], Beatriz Jorrín[2], Núria Lozano[2], Márcio José da Silva[1], Manuel González-Guerrero[2], Laura Migliorini de Araújo[1], Natália Ferreira Verza[1], Homayoun C. Bagheri[3], Juan Imperial[2,4] and Paulo Arruda[1,5,*]

[1]Centro de Biologia Molecular e Engenharia Genética, Universidade Estadual de Campinas (UNICAMP), 13083-875, Campinas, SP, Brazil

[2]Centro de Biotecnología y Genómica de Plantas (CBGP). Universidad Politécnica de Madrid (UPM), 28223 Pozuelo de Alarcón, Madrid, Spain

[3]New Energies Division, Repsol Technology Center, 28935 Mostoles-Madrid, Spain

[4] Consejo Superior de Investigaciones Científicas, Madrid, Spain

[5]Departamento de Genética e Evolução, Instituto de Biologia, Universidade Estadual de Campinas (UNICAMP), 13083-875, Campinas, SP, Brazil

Supplementary Tables

Supplementary Table 1 - Number of raw, unmerged, below QC, chloroplasts, mitochondria and primer-filtered reads per sample for 16S dataset. Demultiplexed raw reads were inputted into the read-processing module of the automated pipeline (Supplementary Fig. 3). Unmerged, low-quality (QC) sequences and sequences of sugarcane plastids were discarded. Sequences without recognizable primer ends were also discarded. The resulting reads (usable) were used for OTU clustering**.**

	**Sample**	**Number of reads**						
		**Raw paired-end**	**Unmerged paired-ends**	**Below QC**	**Chloroplast**	**Mitochondria**	**Primer filter**	**Usable**
Exophytic	Rhizosphere	6,406,547	438,092	616,886	64,294	320,954	158,429	4,807,892
Exophytic	Bottom stalk	18,766,849	2,479,006	1,973,686	113,347	1,044,556	316,817	12,839,437
Exophytic	Medium stalk	14,983,374	1,568,492	1,374,686	60,136	1,202,838	271,937	10,505,285
Exophytic	Upper stalk	11,555,639	1,110,733	1,199,925	63,565	1,134,329	670,190	7,376,897
Exophytic	Leaf	7,955,303	1,137,091	652,253	173,139	1,499,041	90,782	4,402,997
Exophytic	Bulk soil	39,461,273	5,922,837	3,265,103	202,897	1,724,984	1,918,605	26,426,847
Endophytic	Root	12,911,028	916,606	1,125,265	260,227	621,590	315,679	9,671,661
Endophytic	Bottom stalk	12,851,231	2,075,040	1,650,326	2,082,440	3,680,107	76,834	3,286,484
Endophytic	Medium stalk	12,251,593	2,110,722	1,553,816	1,968,319	4,475,064	43,202	2,100,470
Endophytic	Upper stalk	16,092,080	2,274,553	1,893,750	2,676,662	6,629,517	51,457	2,566,141
Endophytic	Leaf	8,564,385	1,193,540	731,012	1,145,570	4,535,408	28,518	930,337
Endophytic	Young shoot	11,707,552	1,312,132	975,475	396,275	2,881,375	195,031	5,947,264
**Total**		**173,506,854**	**22,538,844**	**17,012,183**	**9,206,871**	**29,749,763**	**4,137,481**	**90,861,712**

Supplementary Table 2 - Number of raw R1 reads, below QC, and representing sugarcane sequences on the ITS dataset. Demultiplexed raw reads were imputed in the read-processing module of the automated pipeline (Supplementary Fig. 3). Only the first read (R1) was used for read processing. Low-quality (QC) sequences and sequences of sugarcane genomes were discarded. The resulting reads (usable) were used for OTU clustering.

Sample		Number of reads			
		**Raw R1 reads**	**Below QC**	**Sugarcane**	**Usable**
Exophytic	Rhizosphere	1,537,301	252,069	7,990	1,277,242
	Bottom stalk	17,334,599	5,031,008	111,968	12,191,623
	Medium stalk	35,838,785	9,143,680	188,113	26,506,992
	Upper stalk	18,364,987	7,807,749	1,354,075	9,203,163
	Leaf	6,259,061	898,039	248,609	5,112,413
	Bulk soil	10,396,898	1,654,617	45,617	8,696,664
Endophytic	Root	3,382,608	567,808	30,886	2,783,914
	Bottom stalk	20,653,943	4,999,983	10,367,463	5,286,497
	Medium stalk	25,375,979	6,618,640	13,245,076	5,512,263
	Upper stalk	15,564,397	3,354,085	10,710,630	1,499,682
	Leaf	2,876,956	1,025,934	1,046,744	804,278
	Young shoot	9,807,600	1,225,097	696,722	7,885,781
**Total**		**167,393,114**	**42,578,709**	**38,053,893**	**86,760,512**

Supplementary Table 3 - Prokaryotic orders with significant differences in relative abundance in at least two sample types. The average relative abundance of sample types was used in Kruskal-Wallis tests on the order level to identify taxa whose average abundance differed significantly between sample types. A threshold of P<0,001 (FDR corrected) was used. Un.:Unknown; Exo.: Exophytic; Endo.: Endophytic

Order	P value	P value (FDR corrected)	Relative abundance											
			Bulk Soil	Rhizo.	Endo. Root	Young Shoot	Exo. Bottom Stalk	Endo. Bottom Stalk	Exo. Medium Stalk	Endo. Medium Stalk	Exo. Upper Stalk	Endo. Upper Stalk	Exo. Leaf	Endo. Leaf
Pseudomonadales	0.000	0.000	0.0778%	0.7833%	1.7778%	2.3778%	1.4417%	26.5255%	1.1970%	22.9028%	0.9455%	28.1505%	12.7375%	32.1735%
Enterobacteriales	0.000	0.000	0.0796%	0.2523%	1.1912%	3.5292%	4.8245%	7.5352%	13.6333%	13.7389%	22.7136%	15.6236%	23.7954%	18.7660%
Rhodospirillales	0.000	0.000	10.2296%	8.4796%	5.2593%	6.7319%	9.6792%	7.1569%	16.3722%	5.3977%	16.1722%	6.4472%	1.1699%	0.2253%
Rhizobiales	0.000	0.000	4.3019%	10.4931%	9.3856%	8.2269%	4.8042%	3.3944%	3.6288%	2.6412%	9.2843%	2.8718%	5.2620%	1.0105%
[Saprospirales]	0.000	0.000	4.4852%	9.7074%	11.3870%	11.5505%	4.0819%	1.6778%	4.8323%	3.4917%	5.2773%	3.8866%	0.4102%	0.3710%
Un.	0.000	0.000	0.1204%	0.1671%	12.2056%	2.4162%	19.2282%	0.9051%	13.5359%	0.7653%	8.0480%	0.3560%	1.7653%	1.2994%
Xanthomonadales	0.000	0.000	5.5444%	4.0014%	8.7676%	5.2019%	3.2625%	2.9667%	4.0975%	1.4477%	3.4601%	1.6708%	1.3394%	3.4685%
Sphingobacteriales	0.000	0.000	1.9556%	5.2778%	2.4023%	5.7389%	2.5843%	2.1278%	2.9187%	2.6685%	6.2778%	2.8819%	0.9245%	3.0204%
Legionellales	0.000	0.000	0.7796%	2.6921%	0.7241%	0.8977%	0.2838%	0.0449%	0.4520%	0.0477%	0.7146%	0.0384%	19.6139%	1.6642%
Acidobacteriales	0.000	0.000	4.4556%	2.5319%	2.6931%	10.2208%	1.4278%	0.7375%	0.8823%	0.6880%	1.3571%	0.8713%	0.0514%	0.0753%
Cytophagales	0.000	0.000	1.4037%	2.4639%	5.5977%	3.4639%	1.4384%	0.7426%	0.8187%	0.6477%	1.1076%	0.7079%	0.8477%	0.1043%
Un.	0.000	0.000	0.0185%	0.0111%	0.1792%	0.5569%	0.9630%	2.3130%	0.7480%	4.0574%	1.3066%	2.7588%	0.8144%	5.9142%
Un. Alphaproteobacteria	0.000	0.000	0.5630%	0.4880%	0.5097%	1.2208%	2.8227%	5.3148%	1.7293%	1.3514%	1.3919%	1.6190%	0.6120%	0.1716%
[Chthoniobacterales]	0.000	0.000	0.2370%	4.5185%	1.7264%	3.1231%	0.2620%	0.1593%	0.0212%	0.1301%	0.1783%	0.1394%	0.0074%	0.0049%
Tremblayales	0.000	0.000	0.0000%	0.0014%	0.0000%	0.1120%	0.9583%	0.2199%	3.8162%	1.0347%	6.4444%	1.1898%	0.8093%	0.0932%
Ellin329	0.000	0.000	5.0426%	1.7648%	1.5444%	2.0449%	0.3361%	0.0370%	0.0364%	0.0231%	0.0035%	0.0190%	0.0079%	0.0074%
Solibacterales	0.000	0.000	5.5722%	1.4593%	0.7037%	1.6694%	0.5296%	0.0472%	0.0359%	0.0162%	0.0000%	0.0060%	0.0491%	0.0056%
iii1-15	0.000	0.000	3.9981%	2.9847%	0.7181%	0.3032%	0.4588%	0.0176%	0.0313%	0.0069%	0.0000%	0.0065%	0.0162%	0.0031%
Un. Betaproteobacteria	0.000	0.000	8.1148%	1.3069%	0.1093%	0.1801%	0.1755%	0.0042%	0.0192%	0.0028%	0.0000%	0.0028%	0.0023%	0.0012%
MND1	0.000	0.000	7.1611%	1.7593%	0.1241%	0.0824%	0.2176%	0.0037%	0.0283%	0.0028%	0.0000%	0.0032%	0.0116%	0.0019%
Myxococcales	0.000	0.000	0.7315%	1.2426%	4.4176%	1.8431%	0.5144%	0.0588%	0.0813%	0.0532%	0.0207%	0.0875%	0.2972%	0.0123%
[Pedosphaerales]	0.000	0.000	2.9833%	1.4935%	1.0444%	2.4500%	0.5495%	0.0384%	0.0389%	0.0171%	0.0000%	0.0148%	0.0213%	0.0080%
Actinomycetales	0.000	0.000	1.2111%	0.7380%	1.1255%	0.5060%	1.8005%	0.4995%	1.2586%	0.2690%	0.6419%	0.1620%	0.4574%	0.1315%
Caulobacterales	0.000	0.000	0.1296%	1.2153%	1.1722%	0.7704%	0.4769%	0.2477%	0.4217%	0.4602%	0.5854%	0.3597%	0.1028%	0.3086%
Chlamydiales	0.000	0.000	1.4519%	2.1806%	0.4051%	0.4282%	0.0829%	0.0681%	0.1232%	0.0236%	0.1045%	0.0333%	0.0042%	0.0056%
Bacillales	0.000	0.000	0.5222%	0.4069%	0.1176%	0.3241%	0.6278%	0.6565%	0.3611%	0.3199%	0.0904%	0.3551%	1.4273%	0.3142%
Rubrobacterales	0.000	0.000	0.0000%	0.0005%	0.0148%	0.0042%	0.7199%	1.1222%	0.3773%	1.1528%	0.2460%	0.5060%	0.8593%	0.2593%
Rickettsiales	0.000	0.000	0.1389%	0.2153%	0.0653%	0.1310%	0.1588%	0.2347%	0.1030%	0.0333%	0.2848%	0.0231%	1.5421%	2.0704%
Opitutales	0.000	0.000	0.6796%	1.3806%	0.6403%	0.6028%	0.1560%	0.0088%	0.0101%	0.0079%	0.0020%	0.0060%	0.0005%	0.0025%
Lactobacillales	0.000	0.000	0.0000%	0.0000%	0.0023%	0.0926%	0.8769%	0.5741%	0.4722%	0.3162%	0.0884%	0.1843%	0.3519%	1.0235%
Gemmatales	0.000	0.000	0.1444%	1.4736%	0.1639%	0.4056%	0.0792%	0.0106%	0.0081%	0.0074%	0.0303%	0.0125%	0.0000%	0.0019%
Syntrophobacterales	0.000	0.000	1.1778%	0.8264%	0.3338%	0.2157%	0.2824%	0.0153%	0.0187%	0.0065%	0.0010%	0.0069%	0.0037%	0.0043%
Un. Acidobacteria-5	0.000	0.000	2.6741%	0.2940%	0.1051%	0.1069%	0.1921%	0.0069%	0.0106%	0.0005%	0.0000%	0.0005%	0.0028%	0.0012%
Un. SJA-4	0.000	0.000	0.1463%	1.1458%	0.2454%	0.1551%	0.0370%	0.0037%	0.0040%	0.0120%	0.0000%	0.0106%	0.0005%	0.0031%
Rhodobacterales	0.000	0.000	0.4093%	0.3528%	0.8162%	0.3991%	0.0648%	0.0144%	0.0056%	0.0218%	0.0000%	0.0231%	0.0306%	0.0105%
[Fimbriimonadales]	0.000	0.000	0.0241%	0.7356%	0.4190%	0.3213%	0.0815%	0.0125%	0.0177%	0.0056%	0.0197%	0.0106%	0.0028%	0.0049%
Pirellulales	0.000	0.000	0.2500%	0.6495%	0.2412%	0.3639%	0.1037%	0.0093%	0.0030%	0.0005%	0.0000%	0.0032%	0.0009%	0.0000%
Ellin5290	0.000	0.000	1.7574%	0.1181%	0.0245%	0.0162%	0.0690%	0.0032%	0.0030%	0.0042%	0.0000%	0.0028%	0.0000%	0.0000%
Spirochaetales	0.000	0.000	0.0241%	0.3995%	0.9130%	0.1912%	0.0333%	0.0111%	0.0025%	0.0056%	0.0000%	0.0000%	0.0005%	0.0025%
Methylacidiphilales	0.000	0.000	0.0000%	0.3120%	0.2356%	1.0986%	0.0079%	0.0037%	0.0000%	0.0023%	0.0000%	0.0032%	0.0000%	0.0000%
RB41	0.000	0.000	0.3296%	0.3074%	0.3977%	0.4042%	0.1907%	0.0046%	0.0035%	0.0028%	0.0000%	0.0046%	0.0111%	0.0031%
Un. Gemm-1	0.000	0.000	1.1500%	0.3046%	0.0546%	0.0255%	0.0671%	0.0005%	0.0045%	0.0005%	0.0000%	0.0032%	0.0000%	0.0000%
SBR1031	0.000	0.000	0.1056%	0.5653%	0.3981%	0.1921%	0.0444%	0.0005%	0.0010%	0.0009%	0.0000%	0.0014%	0.0000%	0.0006%
DH61	0.000	0.000	0.0093%	0.8250%	0.1032%	0.0435%	0.0463%	0.0009%	0.0015%	0.0014%	0.0000%	0.0000%	0.0000%	0.0000%
Ellin6067	0.000	0.000	0.7907%	0.3782%	0.0310%	0.0620%	0.0741%	0.0014%	0.0111%	0.0000%	0.0000%	0.0000%	0.0144%	0.0006%
Planctomycetales	0.000	0.000	0.0722%	0.5389%	0.1106%	0.2444%	0.1102%	0.0111%	0.0101%	0.0111%	0.0596%	0.0125%	0.0000%	0.0012%
Pasteurellales	0.000	0.000	0.0000%	0.0000%	0.0005%	0.0255%	0.1269%	0.0690%	0.1965%	0.2903%	0.4848%	0.3454%	0.1046%	0.0364%
Gaiellales	0.000	0.000	0.6648%	0.3005%	0.0477%	0.0477%	0.2241%	0.0037%	0.0242%	0.0032%	0.0000%	0.0009%	0.0218%	0.0006%
Thiotrichales	0.000	0.000	0.9111%	0.2148%	0.0801%	0.0894%	0.0537%	0.0097%	0.0076%	0.0000%	0.0000%	0.0000%	0.0042%	0.0006%
Clostridiales	0.000	0.000	0.0000%	0.0162%	0.0000%	0.7023%	0.0296%	0.0759%	0.0298%	0.2958%	0.0773%	0.2509%	0.0833%	0.0043%
Verrucomicrobiales	0.000	0.000	0.0056%	0.2634%	0.1014%	0.1190%	0.2338%	0.0495%	0.0601%	0.0440%	0.2662%	0.0491%	0.0000%	0.0000%
Un. Gemm-1	0.000	0.000	0.7722%	0.1759%	0.0884%	0.0324%	0.0269%	0.0000%	0.0000%	0.0019%	0.0000%	0.0000%	0.0000%	0.0012%
Ellin6513	0.000	0.000	0.6722%	0.1523%	0.1292%	0.0856%	0.0500%	0.0005%	0.0035%	0.0023%	0.0000%	0.0000%	0.0019%	0.0000%
Neisseriales	0.000	0.000	0.0000%	0.2931%	0.2759%	0.0792%	0.0125%	0.0375%	0.0025%	0.0481%	0.0025%	0.0750%	0.0204%	0.0290%
FAC88	0.000	0.000	0.5481%	0.1602%	0.1139%	0.1356%	0.0352%	0.0014%	0.0020%	0.0014%	0.0000%	0.0056%	0.0023%	0.0006%
Acidimicrobiales	0.000	0.000	0.2833%	0.2949%	0.0606%	0.0444%	0.1185%	0.0102%	0.0040%	0.0032%	0.0000%	0.0060%	0.0083%	0.0006%
Un. Alphaproteobacteria	0.000	0.000	0.0889%	0.2162%	0.3477%	0.1736%	0.0208%	0.0014%	0.0010%	0.0014%	0.0015%	0.0074%	0.0019%	0.0025%
Solirubrobacterales	0.000	0.000	0.3130%	0.1458%	0.0366%	0.1375%	0.2056%	0.0222%	0.0207%	0.0028%	0.0010%	0.0190%	0.0148%	0.0025%
Un. Gammaproteobacteri	0.000	0.000	0.2778%	0.2583%	0.0819%	0.1009%	0.0389%	0.0009%	0.0030%	0.0046%	0.0000%	0.0005%	0.0028%	0.0302%
Un. Betaproteobacteria	0.000	0.000	0.1000%	0.2491%	0.1468%	0.1630%	0.0338%	0.0125%	0.0030%	0.0273%	0.0005%	0.0338%	0.0019%	0.0056%
Anaeroplasmatales	0.000	0.000	0.0000%	0.0074%	0.9648%	0.0194%	0.0023%	0.0042%	0.0000%	0.0023%	0.0000%	0.0000%	0.0009%	0.0037%
BD7-3	0.000	0.000	0.3185%	0.1907%	0.1875%	0.0750%	0.0241%	0.0005%	0.0015%	0.0042%	0.0025%	0.0097%	0.0014%	0.0043%
Sediment-1	0.000	0.000	0.2204%	0.2856%	0.0926%	0.0273%	0.0801%	0.0019%	0.0045%	0.0009%	0.0000%	0.0000%	0.0009%	0.0000%
Nitrososphaerales	0.000	0.000	0.2019%	0.2801%	0.0741%	0.0296%	0.0806%	0.0051%	0.0051%	0.0037%	0.0000%	0.0023%	0.0009%	0.0000%
MLE1-12	0.000	0.000	0.1407%	0.2685%	0.0787%	0.1597%	0.0060%	0.0051%	0.0000%	0.0046%	0.0020%	0.0116%	0.0000%	0.0000%
Un.	0.000	0.000	0.0037%	0.2699%	0.0477%	0.0523%	0.0403%	0.2102%	0.0025%	0.0028%	0.0086%	0.0014%	0.0005%	0.0000%
Rhodocyclales	0.000	0.000	0.0056%	0.2176%	0.0977%	0.1880%	0.0380%	0.0125%	0.0227%	0.0241%	0.0136%	0.0231%	0.0069%	0.0006%
[Cerasicoccales]	0.000	0.000	0.5611%	0.1343%	0.0032%	0.0079%	0.0069%	0.0019%	0.0000%	0.0069%	0.0025%	0.0028%	0.0000%	0.0000%
Alteromonadales	0.000	0.000	0.0167%	0.2718%	0.1148%	0.0995%	0.0454%	0.0005%	0.0015%	0.0000%	0.0000%	0.0028%	0.0028%	0.0012%
Un. Gammaproteobacteri	0.000	0.000	0.2500%	0.1060%	0.2898%	0.0356%	0.0204%	0.0042%	0.0020%	0.0000%	0.0000%	0.0005%	0.0000%	0.0043%
Phycisphaerales	0.000	0.000	0.2741%	0.1046%	0.1097%	0.1903%	0.0213%	0.0023%	0.0000%	0.0028%	0.0000%	0.0060%	0.0005%	0.0000%
258ds10	0.000	0.000	0.0130%	0.2741%	0.1630%	0.0440%	0.0356%	0.0014%	0.0020%	0.0000%	0.0000%	0.0000%	0.0000%	0.0000%
Sva0725	0.000	0.000	0.0463%	0.2532%	0.0986%	0.0227%	0.1139%	0.0000%	0.0131%	0.0005%	0.0000%	0.0000%	0.0014%	0.0000%
Un. Chlamydiia	0.000	0.000	0.0000%	0.3241%	0.0287%	0.0759%	0.0176%	0.0000%	0.0000%	0.0005%	0.0000%	0.0005%	0.0000%	0.0006%
Nitrospirales	0.000	0.000	0.2333%	0.1681%	0.0560%	0.0241%	0.0949%	0.0014%	0.0106%	0.0000%	0.0000%	0.0023%	0.0005%	0.0012%
Cenarchaeales	0.000	0.000	0.7148%	0.0130%	0.0009%	0.0000%	0.0060%	0.0005%	0.0000%	0.0009%	0.0000%	0.0042%	0.0000%	0.0000%
Bdellovibrionales	0.000	0.000	0.1815%	0.0667%	0.0889%	0.0958%	0.0370%	0.0009%	0.0045%	0.0718%	0.0596%	0.0699%	0.0056%	0.0006%
Un.	0.000	0.000	0.1796%	0.1949%	0.0546%	0.0903%	0.0093%	0.0005%	0.0005%	0.0005%	0.0000%	0.0000%	0.0000%	0.0000%
Un. S085	0.000	0.000	0.3444%	0.1662%	0.0088%	0.0120%	0.0231%	0.0000%	0.0005%	0.0005%	0.0000%	0.0005%	0.0014%	0.0000%
Un.	0.000	0.000	0.2074%	0.0000%	0.0000%	0.0000%	0.0069%	0.1366%	0.0126%	0.1301%	0.0035%	0.0958%	0.0019%	0.1235%
[Entotheonellales]	0.000	0.000	0.3648%	0.1319%	0.0157%	0.0037%	0.0157%	0.0014%	0.0000%	0.0000%	0.0000%	0.0000%	0.0000%	0.0000%
Nov-24	0.000	0.000	0.0648%	0.1329%	0.1157%	0.2079%	0.0051%	0.0005%	0.0000%	0.0000%	0.0000%	0.0000%	0.0005%	0.0000%
B07_WMSP1	0.000	0.000	0.2167%	0.1694%	0.0019%	0.0046%	0.0222%	0.0014%	0.0000%	0.0009%	0.0000%	0.0000%	0.0005%	0.0000%
Un. SBRH58	0.000	0.000	0.0407%	0.2181%	0.0208%	0.0528%	0.0194%	0.0037%	0.0015%	0.0046%	0.0000%	0.0000%	0.0000%	0.0000%
Un. Mb-NB09	0.000	0.000	0.1019%	0.1755%	0.1144%	0.0000%	0.0028%	0.0000%	0.0000%	0.0000%	0.0000%	0.0019%	0.0000%	0.0000%
Un. TM7-1	0.000	0.000	0.3296%	0.0898%	0.0241%	0.0056%	0.0023%	0.0000%	0.0000%	0.0042%	0.0126%	0.0028%	0.0037%	0.0000%
IS-44	0.000	0.000	0.1185%	0.1106%	0.0398%	0.1208%	0.0542%	0.0000%	0.0061%	0.0000%	0.0000%	0.0000%	0.0000%	0.0000%
AKYG885	0.000	0.000	0.0574%	0.2171%	0.0213%	0.0088%	0.0329%	0.0000%	0.0010%	0.0005%	0.0000%	0.0000%	0.0000%	0.0000%
KD8-87	0.000	0.000	0.3722%	0.0755%	0.0167%	0.0051%	0.0093%	0.0000%	0.0000%	0.0000%	0.0000%	0.0000%	0.0000%	0.0000%
SC-I-84	0.000	0.000	0.0648%	0.1009%	0.0324%	0.1491%	0.0495%	0.0046%	0.0030%	0.0023%	0.0000%	0.0000%	0.0014%	0.0000%
SM1D11	0.000	0.000	0.0407%	0.1046%	0.0384%	0.0718%	0.0718%	0.0009%	0.0000%	0.0032%	0.0631%	0.0042%	0.0009%	0.0000%
CCU21	0.000	0.000	0.0093%	0.1375%	0.1662%	0.0213%	0.0106%	0.0023%	0.0000%	0.0000%	0.0000%	0.0000%	0.0009%	0.0000%
Un.	0.000	0.000	0.3704%	0.0361%	0.0157%	0.0083%	0.0148%	0.0000%	0.0005%	0.0000%	0.0000%	0.0000%	0.0000%	0.0000%
Un. Chlamydiia	0.000	0.000	0.0037%	0.2046%	0.0375%	0.0185%	0.0028%	0.0000%	0.0010%	0.0019%	0.0000%	0.0051%	0.0000%	0.0000%

MIZ46	0.000	0.000	0.2093%	0.0440%	0.0755%	0.0736%	0.0208%	0.0005%	0.0015%	0.0037%	0.0000%	0.0009%	0.0000%	0.0012%
Un. Gemmatimonadetes	0.000	0.000	0.2500%	0.0500%	0.0162%	0.0218%	0.0676%	0.0028%	0.0030%	0.0028%	0.0000%	0.0000%	0.0079%	0.0000%
Un. Deltaproteobacteria	0.000	0.000	0.1185%	0.0963%	0.0602%	0.0537%	0.0060%	0.0000%	0.0000%	0.0019%	0.0000%	0.0009%	0.0000%	0.0000%
PK29	0.000	0.000	0.0963%	0.0537%	0.1583%	0.0181%	0.0236%	0.0028%	0.0005%	0.0009%	0.0000%	0.0019%	0.0009%	0.0000%
Un.	0.000	0.000	0.3315%	0.0222%	0.0014%	0.0019%	0.0028%	0.0000%	0.0000%	0.0000%	0.0000%	0.0000%	0.0000%	0.0000%
WD2101	0.000	0.000	0.1296%	0.0282%	0.0338%	0.1000%	0.0343%	0.0074%	0.0015%	0.0019%	0.0071%	0.0019%	0.0056%	0.0000%
FW68	0.000	0.000	0.0019%	0.1287%	0.0431%	0.0472%	0.0000%	0.0000%	0.0000%	0.0000%	0.0000%	0.0000%	0.0000%	0.0000%
Un. Ellin6529	0.000	0.000	0.0741%	0.0884%	0.0269%	0.0111%	0.0486%	0.0000%	0.0025%	0.0037%	0.0000%	0.0019%	0.0000%	0.0000%
Procabacteriales	0.000	0.000	0.0093%	0.0875%	0.0060%	0.0477%	0.0042%	0.0338%	0.0010%	0.0185%	0.0000%	0.0264%	0.0042%	0.0068%
Armatimonadales	0.000	0.000	0.0000%	0.0926%	0.0310%	0.0440%	0.0278%	0.0069%	0.0242%	0.0005%	0.0066%	0.0000%	0.0005%	0.0000%
Aeromonadales	0.000	0.000	0.0000%	0.0000%	0.0005%	0.0000%	0.0000%	0.1185%	0.0000%	0.0815%	0.0000%	0.1176%	0.0000%	0.0056%
Un.	0.000	0.000	0.0148%	0.1051%	0.0352%	0.0514%	0.0042%	0.0000%	0.0000%	0.0000%	0.0000%	0.0009%	0.0000%	0.0019%
Un.	0.000	0.000	0.1278%	0.0861%	0.0111%	0.0014%	0.0005%	0.0000%	0.0000%	0.0000%	0.0000%	0.0000%	0.0000%	0.0000%
CL500-15	0.000	0.000	0.1204%	0.0667%	0.0269%	0.0139%	0.0139%	0.0000%	0.0000%	0.0000%	0.0000%	0.0005%	0.0000%	0.0000%
Un.	0.000	0.000	0.1259%	0.0278%	0.0454%	0.0264%	0.0431%	0.0000%	0.0051%	0.0000%	0.0000%	0.0000%	0.0000%	0.0000%
Un.	0.000	0.000	0.0241%	0.0167%	0.0444%	0.0505%	0.1259%	0.0111%	0.0005%	0.0000%	0.0000%	0.0000%	0.0079%	0.0000%
IIb	0.000	0.000	0.2056%	0.0366%	0.0046%	0.0060%	0.0056%	0.0005%	0.0000%	0.0000%	0.0000%	0.0000%	0.0019%	0.0000%
Un. SJA-28	0.000	0.000	0.0333%	0.1111%	0.0264%	0.0023%	0.0079%	0.0000%	0.0000%	0.0000%	0.0000%	0.0000%	0.0000%	0.0000%
GIF10	0.000	0.000	0.1111%	0.0444%	0.0597%	0.0083%	0.0032%	0.0000%	0.0000%	0.0000%	0.0000%	0.0000%	0.0000%	0.0000%
Kiloniellales	0.000	0.000	0.0000%	0.0565%	0.0671%	0.0731%	0.0009%	0.0000%	0.0000%	0.0019%	0.0000%	0.0120%	0.0000%	0.0000%
Un. 0319-6E2	0.000	0.000	0.0167%	0.1181%	0.0069%	0.0037%	0.0009%	0.0000%	0.0005%	0.0000%	0.0000%	0.0014%	0.0005%	0.0000%
Un. ABY1	0.000	0.000	0.1426%	0.0454%	0.0042%	0.0046%	0.0000%	0.0000%	0.0000%	0.0000%	0.0000%	0.0000%	0.0000%	0.0000%
PHOS-HD29	0.000	0.000	0.0037%	0.0866%	0.0241%	0.0292%	0.0037%	0.0000%	0.0000%	0.0032%	0.0000%	0.0009%	0.0000%	0.0006%
DS-18	0.000	0.000	0.0259%	0.0847%	0.0167%	0.0019%	0.0139%	0.0000%	0.0035%	0.0000%	0.0000%	0.0000%	0.0009%	0.0006%
Desulfuromonadales	0.000	0.000	0.0000%	0.0880%	0.0023%	0.0458%	0.0000%	0.0000%	0.0000%	0.0000%	0.0000%	0.0000%	0.0051%	0.0000%
WCHD3-30	0.000	0.000	0.1944%	0.0106%	0.0000%	0.0000%	0.0042%	0.0000%	0.0000%	0.0000%	0.0000%	0.0000%	0.0000%	0.0000%
Un. EC1113	0.000	0.000	0.0907%	0.0542%	0.0009%	0.0046%	0.0116%	0.0000%	0.0000%	0.0000%	0.0000%	0.0000%	0.0000%	0.0000%
Un. TM1	0.000	0.000	0.1333%	0.0301%	0.0120%	0.0069%	0.0005%	0.0000%	0.0000%	0.0000%	0.0000%	0.0000%	0.0014%	0.0000%
[Roseiflexales]	0.000	0.000	0.0074%	0.0569%	0.0644%	0.0139%	0.0074%	0.0000%	0.0000%	0.0000%	0.0000%	0.0000%	0.0000%	0.0000%
CFB-26	0.000	0.000	0.0426%	0.0588%	0.0403%	0.0009%	0.0009%	0.0000%	0.0000%	0.0000%	0.0000%	0.0000%	0.0000%	0.0000%
Un.	0.000	0.000	0.0296%	0.0352%	0.0028%	0.0889%	0.0069%	0.0005%	0.0005%	0.0009%	0.0000%	0.0005%	0.0000%	0.0006%
p04_C01	0.000	0.000	0.0148%	0.0667%	0.0171%	0.0139%	0.0093%	0.0000%	0.0000%	0.0000%	0.0000%	0.0000%	0.0000%	0.0000%
Un.	0.000	0.000	0.0963%	0.0306%	0.0111%	0.0014%	0.0037%	0.0000%	0.0000%	0.0000%	0.0000%	0.0000%	0.0000%	0.0000%
PK329	0.000	0.000	0.1130%	0.0204%	0.0139%	0.0000%	0.0042%	0.0000%	0.0000%	0.0000%	0.0000%	0.0000%	0.0005%	0.0000%
NB1-j	0.000	0.000	0.0278%	0.0250%	0.0787%	0.0019%	0.0083%	0.0009%	0.0000%	0.0009%	0.0000%	0.0000%	0.0023%	0.0000%
Un.	0.000	0.000	0.0259%	0.0417%	0.0384%	0.0111%	0.0106%	0.0000%	0.0010%	0.0000%	0.0000%	0.0000%	0.0000%	0.0000%
Un. VHS-B5-50	0.000	0.000	0.0444%	0.0583%	0.0060%	0.0005%	0.0028%	0.0000%	0.0000%	0.0000%	0.0000%	0.0000%	0.0000%	0.0000%
Holophagales	0.000	0.000	0.0037%	0.0648%	0.0102%	0.0093%	0.0060%	0.0009%	0.0020%	0.0069%	0.0000%	0.0009%	0.0000%	0.0000%
HTCC2188	0.000	0.000	0.0204%	0.0352%	0.0231%	0.0389%	0.0130%	0.0000%	0.0015%	0.0000%	0.0000%	0.0000%	0.0000%	0.0000%
Spirobacillales	0.000	0.000	0.0037%	0.0162%	0.0722%	0.0181%	0.0269%	0.0014%	0.0000%	0.0000%	0.0005%	0.0000%	0.0023%	0.0093%
Un. OPB56	0.000	0.000	0.0056%	0.0495%	0.0130%	0.0278%	0.0167%	0.0000%	0.0015%	0.0000%	0.0000%	0.0000%	0.0005%	0.0000%
Un. 3BR-5F	0.000	0.000	0.0037%	0.0713%	0.0120%	0.0023%	0.0000%	0.0000%	0.0000%	0.0000%	0.0000%	0.0000%	0.0000%	0.0000%
agg27	0.000	0.000	0.0296%	0.0324%	0.0310%	0.0194%	0.0093%	0.0000%	0.0000%	0.0000%	0.0000%	0.0000%	0.0000%	0.0000%
[Leptospirales]	0.000	0.000	0.0000%	0.0236%	0.0815%	0.0130%	0.0023%	0.0000%	0.0000%	0.0014%	0.0000%	0.0009%	0.0000%	0.0006%
Un. SM2F11	0.000	0.000	0.1185%	0.0097%	0.0019%	0.0060%	0.0000%	0.0000%	0.0000%	0.0000%	0.0000%	0.0000%	0.0000%	0.0000%
Un. SC3	0.000	0.000	0.0667%	0.0343%	0.0069%	0.0005%	0.0009%	0.0000%	0.0000%	0.0000%	0.0000%	0.0000%	0.0000%	0.0000%
Un. VC2_1_Bac22	0.000	0.000	0.0000%	0.0120%	0.0403%	0.0681%	0.0065%	0.0014%	0.0000%	0.0005%	0.0000%	0.0009%	0.0005%	0.0000%
Un.	0.000	0.000	0.0593%	0.0301%	0.0185%	0.0000%	0.0000%	0.0000%	0.0000%	0.0000%	0.0000%	0.0000%	0.0000%	0.0000%
Un. ML635J-21	0.000	0.000	0.0148%	0.0491%	0.0005%	0.0125%	0.0065%	0.0000%	0.0020%	0.0000%	0.0000%	0.0000%	0.0014%	0.0012%
MVS-107	0.000	0.000	0.0426%	0.0370%	0.0079%	0.0023%	0.0060%	0.0000%	0.0000%	0.0000%	0.0000%	0.0000%	0.0000%	0.0000%
Elusimicrobiales	0.000	0.000	0.0944%	0.0162%	0.0009%	0.0023%	0.0009%	0.0000%	0.0000%	0.0000%	0.0000%	0.0000%	0.0000%	0.0000%
CV90	0.000	0.000	0.0000%	0.0481%	0.0139%	0.0162%	0.0023%	0.0000%	0.0000%	0.0000%	0.0000%	0.0019%	0.0000%	0.0000%
Un. Gemm-2	0.000	0.000	0.0630%	0.0264%	0.0000%	0.0005%	0.0014%	0.0000%	0.0000%	0.0000%	0.0000%	0.0000%	0.0000%	0.0000%
Chromatiales	0.000	0.000	0.0093%	0.0227%	0.0532%	0.0032%	0.0032%	0.0000%	0.0000%	0.0019%	0.0000%	0.0000%	0.0000%	0.0000%
Un. Deltaproteobacteria	0.000	0.000	0.0333%	0.0343%	0.0069%	0.0037%	0.0028%	0.0005%	0.0000%	0.0000%	0.0000%	0.0000%	0.0000%	0.0000%
Un. PAUC37f	0.000	0.000	0.0241%	0.0269%	0.0134%	0.0097%	0.0125%	0.0000%	0.0005%	0.0000%	0.0000%	0.0000%	0.0000%	0.0000%
Un.	0.000	0.000	0.0574%	0.0259%	0.0000%	0.0000%	0.0000%	0.0000%	0.0000%	0.0014%	0.0000%	0.0000%	0.0000%	0.0000%
Un.	0.000	0.000	0.0333%	0.0282%	0.0088%	0.0014%	0.0065%	0.0000%	0.0000%	0.0000%	0.0000%	0.0000%	0.0000%	0.0006%
TK10	0.000	0.000	0.0481%	0.0255%	0.0000%	0.0032%	0.0028%	0.0000%	0.0000%	0.0000%	0.0000%	0.0000%	0.0000%	0.0000%
ArcA07	0.000	0.000	0.1019%	0.0000%	0.0000%	0.0000%	0.0000%	0.0000%	0.0000%	0.0000%	0.0000%	0.0000%	0.0000%	0.0000%
B97	0.000	0.000	0.0000%	0.0356%	0.0255%	0.0042%	0.0000%	0.0000%	0.0000%	0.0000%	0.0000%	0.0000%	0.0000%	0.0000%
Gemellales	0.000	0.000	0.0000%	0.0000%	0.0000%	0.0000%	0.0074%	0.0250%	0.0000%	0.0370%	0.0005%	0.0083%	0.0218%	0.0000%
YLA114	0.000	0.000	0.0778%	0.0074%	0.0014%	0.0009%	0.0023%	0.0000%	0.0000%	0.0000%	0.0000%	0.0000%	0.0000%	0.0000%
Un.	0.000	0.000	0.0389%	0.0250%	0.0019%	0.0009%	0.0014%	0.0000%	0.0000%	0.0009%	0.0000%	0.0000%	0.0005%	0.0000%
Un.	0.000	0.000	0.0222%	0.0190%	0.0111%	0.0069%	0.0065%	0.0019%	0.0010%	0.0000%	0.0000%	0.0000%	0.0000%	0.0000%
N1423WL	0.000	0.000	0.0352%	0.0120%	0.0051%	0.0139%	0.0079%	0.0000%	0.0000%	0.0000%	0.0000%	0.0005%	0.0000%	0.0006%
Un. Gemm-3	0.000	0.000	0.0389%	0.0088%	0.0005%	0.0032%	0.0176%	0.0000%	0.0010%	0.0000%	0.0000%	0.0000%	0.0060%	0.0000%
Un.	0.000	0.000	0.0796%	0.0000%	0.0000%	0.0014%	0.0009%	0.0000%	0.0000%	0.0009%	0.0000%	0.0000%	0.0000%	0.0000%
d113	0.000	0.000	0.0241%	0.0255%	0.0019%	0.0000%	0.0005%	0.0000%	0.0000%	0.0000%	0.0000%	0.0009%	0.0000%	0.0000%
Fusobacteriales	0.000	0.000	0.0000%	0.0000%	0.0000%	0.0000%	0.0032%	0.0125%	0.0000%	0.0176%	0.0000%	0.0167%	0.0278%	0.0000%
Un. C0119	0.000	0.000	0.0000%	0.0171%	0.0032%	0.0347%	0.0042%	0.0000%	0.0000%	0.0000%	0.0000%	0.0000%	0.0005%	0.0000%
LD1-PA13	0.000	0.000	0.0500%	0.0093%	0.0023%	0.0042%	0.0009%	0.0005%	0.0000%	0.0000%	0.0000%	0.0000%	0.0000%	0.0000%
Un. [Chloracidobacteria]	0.000	0.000	0.0148%	0.0194%	0.0065%	0.0051%	0.0065%	0.0000%	0.0005%	0.0019%	0.0000%	0.0000%	0.0000%	0.0000%
Mariprofundales	0.000	0.000	0.0259%	0.0106%	0.0130%	0.0028%	0.0083%	0.0000%	0.0010%	0.0000%	0.0000%	0.0000%	0.0000%	0.0000%
H39	0.000	0.000	0.0093%	0.0213%	0.0102%	0.0042%	0.0032%	0.0000%	0.0000%	0.0000%	0.0000%	0.0005%	0.0000%	0.0000%
Un. P2-11E	0.000	0.000	0.0537%	0.0074%	0.0000%	0.0000%	0.0000%	0.0000%	0.0000%	0.0005%	0.0000%	0.0000%	0.0000%	0.0000%
Un. BME43	0.000	0.000	0.0000%	0.0139%	0.0259%	0.0106%	0.0042%	0.0000%	0.0000%	0.0000%	0.0000%	0.0000%	0.0005%	0.0000%
Un. koll11	0.000	0.000	0.0426%	0.0079%	0.0074%	0.0019%	0.0000%	0.0000%	0.0000%	0.0000%	0.0000%	0.0000%	0.0000%	0.0000%
envOPS12	0.000	0.000	0.0204%	0.0185%	0.0042%	0.0019%	0.0037%	0.0000%	0.0000%	0.0000%	0.0000%	0.0000%	0.0000%	0.0000%
Un. MSB-5A5	0.000	0.000	0.0111%	0.0273%	0.0005%	0.0000%	0.0009%	0.0000%	0.0000%	0.0000%	0.0000%	0.0000%	0.0000%	0.0000%
Ktedonobacterales	0.000	0.000	0.0019%	0.0042%	0.0306%	0.0153%	0.0056%	0.0009%	0.0005%	0.0019%	0.0000%	0.0000%	0.0005%	0.0000%
0319-7L14	0.000	0.000	0.0185%	0.0167%	0.0005%	0.0005%	0.0102%	0.0005%	0.0000%	0.0009%	0.0000%	0.0000%	0.0000%	0.0000%
Un.	0.000	0.000	0.0019%	0.0019%	0.0000%	0.0014%	0.0019%	0.0083%	0.0030%	0.0088%	0.0000%	0.0065%	0.0000%	0.0222%
A89	0.000	0.000	0.0056%	0.0222%	0.0051%	0.0005%	0.0009%	0.0000%	0.0000%	0.0000%	0.0000%	0.0000%	0.0000%	0.0000%
Un. OP11-4	0.000	0.000	0.0463%	0.0046%	0.0000%	0.0000%	0.0000%	0.0000%	0.0000%	0.0000%	0.0000%	0.0000%	0.0000%	0.0000%
d153	0.000	0.000	0.0037%	0.0236%	0.0005%	0.0005%	0.0023%	0.0000%	0.0000%	0.0000%	0.0000%	0.0000%	0.0000%	0.0000%
DS-100	0.000	0.000	0.0000%	0.0069%	0.0273%	0.0074%	0.0028%	0.0000%	0.0000%	0.0000%	0.0000%	0.0000%	0.0000%	0.0000%
Un. Opitutae	0.000	0.000	0.0130%	0.0069%	0.0213%	0.0000%	0.0009%	0.0000%	0.0000%	0.0000%	0.0000%	0.0000%	0.0000%	0.0000%
Un.	0.000	0.000	0.0000%	0.0213%	0.0000%	0.0046%	0.0014%	0.0000%	0.0000%	0.0000%	0.0000%	0.0000%	0.0000%	0.0000%
Un.	0.000	0.000	0.0037%	0.0190%	0.0000%	0.0009%	0.0056%	0.0000%	0.0000%	0.0000%	0.0000%	0.0000%	0.0000%	0.0000%
I025	0.000	0.000	0.0111%	0.0153%	0.0046%	0.0009%	0.0000%	0.0000%	0.0000%	0.0000%	0.0000%	0.0000%	0.0000%	0.0000%
JH-WHS99	0.000	0.000	0.0093%	0.0181%	0.0000%	0.0000%	0.0000%	0.0000%	0.0000%	0.0000%	0.0000%	0.0000%	0.0000%	0.0000%
HOC36	0.001	0.001	0.0000%	0.0125%	0.0019%	0.0102%	0.0079%	0.0005%	0.0000%	0.0000%	0.0000%	0.0000%	0.0000%	0.0000%
mle1-48	0.000	0.000	0.0093%	0.0144%	0.0000%	0.0014%	0.0032%	0.0000%	0.0000%	0.0000%	0.0000%	0.0000%	0.0005%	0.0000%
Un.	0.000	0.000	0.0000%	0.0134%	0.0042%	0.0065%	0.0023%	0.0014%	0.0000%	0.0000%	0.0000%	0.0000%	0.0000%	0.0000%
C114	0.000	0.000	0.0000%	0.0176%	0.0000%	0.0000%	0.0056%	0.0000%	0.0000%	0.0000%	0.0000%	0.0000%	0.0000%	0.0000%
Un. TK10	0.000	0.000	0.0130%	0.0116%	0.0000%	0.0000%	0.0037%	0.0000%	0.0000%	0.0000%	0.0000%	0.0000%	0.0000%	0.0000%
Un. SHA-109	0.000	0.000	0.0000%	0.0167%	0.0000%	0.0000%	0.0060%	0.0000%	0.0000%	0.0000%	0.0000%	0.0000%	0.0000%	0.0000%
32-20	0.000	0.000	0.0111%	0.0056%	0.0088%	0.0037%	0.0046%	0.0000%	0.0000%	0.0000%	0.0000%	0.0000%	0.0000%	0.0000%
Un. Ktedonobacteria	0.000	0.000	0.0204%	0.0069%	0.0000%	0.0014%	0.0032%	0.0000%	0.0000%	0.0000%	0.0000%	0.0000%	0.0000%	0.0000%
JG30-KF-AS9	0.000	0.000	0.0204%	0.0009%	0.0000%	0.0088%	0.0065%	0.0000%	0.0000%	0.0000%	0.0000%	0.0000%	0.0000%	0.0000%
Ellin7246	0.000	0.000	0.0074%	0.0139%	0.0014%	0.0000%	0.0000%	0.0000%	0.0000%	0.0000%	0.0000%	0.0000%	0.0009%	0.0000%
SJA-22	0.000	0.000	0.0000%	0.0005%	0.0120%	0.0245%	0.0000%	0.0000%	0.0000%	0.0000%	0.0000%	0.0000%	0.0000%	0.0000%
A31	0.000	0.000	0.0037%	0.0130%	0.0032%	0.0000%	0.0000%	0.0000%	0.0000%	0.0032%	0.0000%	0.0000%	0.0000%	0.0000%
CPla-3	0.000	0.000	0.0037%	0.0032%	0.0046%	0.0194%	0.0014%	0.0000%	0.0000%	0.0000%	0.0000%	0.0000%	0.0000%	0.0000%
Un. TK17	0.000	0.000	0.0241%	0.0056%	0.0000%	0.0000%	0.0000%	0.0000%	0.0000%	0.0000%	0.0000%	0.0000%	0.0000%	0.0000%

Un. Endomicrobia	0.000	0.000	0.0093%	0.0097%	0.0060%	0.0005%	0.0000%	0.0000%	0.0000%	0.0000%	0.0000%	0.0000%	0.0000%	0.0000%
Micrococcales	0.000	0.000	0.0093%	0.0093%	0.0000%	0.0000%	0.0056%	0.0000%	0.0000%	0.0000%	0.0000%	0.0000%	0.0000%	0.0000%
MVP-88	0.000	0.000	0.0259%	0.0023%	0.0014%	0.0000%	0.0000%	0.0000%	0.0000%	0.0000%	0.0000%	0.0000%	0.0000%	0.0000%
GCA004	0.000	0.000	0.0000%	0.0153%	0.0005%	0.0000%	0.0000%	0.0000%	0.0000%	0.0000%	0.0000%	0.0000%	0.0000%	0.0000%
Un. GN07	0.000	0.000	0.0000%	0.0148%	0.0009%	0.0005%	0.0000%	0.0000%	0.0000%	0.0000%	0.0000%	0.0000%	0.0000%	0.0000%
FAC87	0.000	0.000	0.0093%	0.0014%	0.0005%	0.0134%	0.0037%	0.0000%	0.0000%	0.0009%	0.0000%	0.0000%	0.0000%	0.0000%
Pla1	0.000	0.000	0.0148%	0.0056%	0.0000%	0.0005%	0.0019%	0.0000%	0.0000%	0.0019%	0.0000%	0.0000%	0.0000%	0.0000%
S1198	0.000	0.000	0.0000%	0.0134%	0.0000%	0.0000%	0.0009%	0.0000%	0.0000%	0.0000%	0.0000%	0.0000%	0.0000%	0.0006%
Un. Gemmatimonadetes	0.001	0.001	0.0167%	0.0023%	0.0000%	0.0000%	0.0014%	0.0000%	0.0015%	0.0000%	0.0000%	0.0000%	0.0014%	0.0000%
Un.	0.000	0.000	0.0093%	0.0074%	0.0000%	0.0000%	0.0000%	0.0000%	0.0000%	0.0000%	0.0000%	0.0000%	0.0000%	0.0000%
Un. S035	0.000	0.000	0.0111%	0.0042%	0.0000%	0.0000%	0.0046%	0.0000%	0.0000%	0.0000%	0.0000%	0.0000%	0.0000%	0.0000%
Un.	0.000	0.000	0.0000%	0.0116%	0.0000%	0.0000%	0.0000%	0.0000%	0.0000%	0.0000%	0.0000%	0.0000%	0.0000%	0.0000%
Chthonomonadales	0.000	0.000	0.0019%	0.0009%	0.0000%	0.0153%	0.0028%	0.0005%	0.0000%	0.0005%	0.0000%	0.0000%	0.0000%	0.0000%
Un.	0.000	0.000	0.0222%	0.0000%	0.0000%	0.0000%	0.0000%	0.0000%	0.0000%	0.0000%	0.0000%	0.0000%	0.0000%	0.0000%
Un. IIB17	0.000	0.000	0.0000%	0.0069%	0.0074%	0.0000%	0.0000%	0.0000%	0.0000%	0.0000%	0.0000%	0.0000%	0.0000%	0.0000%
Un. Thermomicrobia	0.000	0.000	0.0167%	0.0000%	0.0000%	0.0000%	0.0019%	0.0000%	0.0000%	0.0000%	0.0000%	0.0000%	0.0000%	0.0000%
Un. PBS-25	0.000	0.000	0.0000%	0.0069%	0.0000%	0.0009%	0.0000%	0.0000%	0.0000%	0.0009%	0.0000%	0.0000%	0.0000%	0.0000%
Mycoplasmatales	0.000	0.000	0.0000%	0.0000%	0.0000%	0.0000%	0.0005%	0.0009%	0.0000%	0.0074%	0.0000%	0.0037%	0.0000%	0.0025%
Un.	0.000	0.000	0.0148%	0.0000%	0.0000%	0.0000%	0.0000%	0.0000%	0.0000%	0.0000%	0.0000%	0.0000%	0.0000%	0.0000%
Un.	0.000	0.000	0.0093%	0.0019%	0.0000%	0.0000%	0.0014%	0.0000%	0.0000%	0.0000%	0.0000%	0.0000%	0.0000%	0.0000%
Caldilineales	0.000	0.000	0.0000%	0.0046%	0.0000%	0.0000%	0.0032%	0.0000%	0.0000%	0.0000%	0.0000%	0.0000%	0.0000%	0.0000%
Un. BD7-11	0.000	0.000	0.0000%	0.0051%	0.0000%	0.0019%	0.0005%	0.0000%	0.0000%	0.0000%	0.0000%	0.0000%	0.0000%	0.0000%
Nitrosomonadales	0.000	0.000	0.0074%	0.0000%	0.0000%	0.0014%	0.0000%	0.0000%	0.0000%	0.0000%	0.0000%	0.0005%	0.0019%	0.0000%
SJA-36	0.001	0.001	0.0000%	0.0037%	0.0032%	0.0000%	0.0000%	0.0000%	0.0000%	0.0000%	0.0000%	0.0000%	0.0000%	0.0000%
Un. SAR202	0.000	0.000	0.0037%	0.0000%	0.0000%	0.0000%	0.0000%	0.0000%	0.0000%	0.0000%	0.0000%	0.0000%	0.0000%	0.0000%
CCM11a	0.000	0.000	0.0037%	0.0000%	0.0000%	0.0000%	0.0000%	0.0000%	0.0000%	0.0000%	0.0000%	0.0000%	0.0000%	0.0000%
E2	0.000	0.000	0.0019%	0.0000%	0.0000%	0.0000%	0.0000%	0.0000%	0.0000%	0.0000%	0.0000%	0.0000%	0.0000%	0.0000%

Supplementary Table 4 - Fungal orders with significant differences in relative abundance in at least two sample types. The average relative abundance of sample types was used in Kruskal-Wallis tests on the order level to identify taxa whose average abundance differed significantly between sample types. A threshold of P<0,001 (FDR corrected) was used. Un.:Unknown; Exo.: Exophytic; Endo.: Endophytic

Order	P value	P value (FDR corrected)	Sample Type											
			Bulk Soil	Rhizosphere	Endophytic Root	Young Shoot	Exophytic Bottom Stalk	Endophytic Bottom Stalk	Exophytic Medium Stalk	Endophytic Medium Stalk	Exophytic Upper Stalk	Endophytic Upper Stalk	Exophytic Leaf	Endophytic Leaf
Capnodiales	0.000	0.000	7.1139%	2.4167%	0.1232%	0.2813%	48.6898%	36.2167%	20.9146%	23.1620%	28.5866%	12.8742%	9.8318%	13.1617%
Unkown	0.000	0.000	36.9236%	12.8045%	8.3030%	11.1187%	1.0380%	4.3801%	2.6136%	4.9051%	7.9134%	12.7091%	33.8894%	47.3222%
Eurotiales	0.000	0.000	7.0000%	8.1828%	11.7480%	1.9460%	8.2625%	12.5750%	23.1045%	1.4231%	8.4403%	14.7758%	8.0783%	0.4389%
Saccharomycetales	0.000	0.000	0.2708%	0.9712%	0.0040%	0.2500%	10.6287%	16.8102%	19.5939%	1.2435%	14.2269%	17.8914%	6.6323%	1.1011%
Polyporales	0.000	0.000	0.0778%	23.1490%	16.4939%	32.8298%	0.0148%	0.0005%	0.0101%	0.1222%	0.0259%	2.5813%	0.2288%	0.4011%
Unkown	0.000	0.000	4.4681%	4.0970%	3.3899%	2.0818%	5.0870%	7.8824%	9.8914%	1.0028%	3.5995%	4.8081%	9.2005%	10.5078%
Hypocreales	0.000	0.000	3.0917%	1.4934%	2.0672%	2.4742%	1.8634%	1.7074%	4.5545%	0.2583%	18.4843%	14.2419%	7.7136%	0.2783%
Unkown Agaricomycetes	0.000	0.000	3.1625%	19.0298%	17.8818%	14.4419%	0.2208%	0.0519%	0.0222%	0.1407%	0.4602%	0.2005%	0.3025%	1.1233%
Pleosporales	0.000	0.000	1.0083%	0.7328%	1.4172%	0.4116%	0.7167%	1.3750%	0.4465%	24.9972%	0.9319%	0.6298%	0.1899%	4.3078%
Unkown	0.000	0.000	4.1819%	4.8389%	11.6202%	3.5439%	0.1620%	0.0690%	0.0303%	0.1287%	1.3602%	1.2859%	3.5470%	4.8883%
Chaetothyriales	0.000	0.000	0.4306%	1.4914%	1.0530%	5.3414%	7.9773%	6.5528%	5.1126%	0.1870%	3.2065%	2.7187%	1.3646%	0.0861%
Ustilaginales	0.000	0.000	0.0111%	0.1172%	0.0000%	0.0121%	1.3824%	0.9838%	1.1854%	17.7958%	0.2662%	0.9409%	0.0414%	12.1111%
Helotiales	0.000	0.000	0.0569%	0.0106%	0.0010%	0.0237%	0.8329%	0.4495%	2.7187%	0.0806%	2.4236%	6.7429%	17.2399%	0.0600%
Tremellales	0.000	0.000	0.3528%	0.4480%	0.1985%	0.4414%	7.2662%	5.8171%	6.6530%	0.7236%	2.3338%	1.4157%	0.5096%	0.3561%
Unkown Sordariomycetes	0.000	0.000	0.7611%	3.8707%	4.1808%	7.2030%	0.1898%	0.0764%	0.0889%	0.0296%	0.3856%	0.4783%	0.1136%	0.0178%
Agaricales	0.000	0.000	6.3333%	3.8949%	4.3611%	1.7995%	0.0269%	0.0014%	0.0126%	0.2856%	0.0000%	0.0091%	0.0904%	0.0461%
Filobasidiales	0.000	0.000	0.2444%	0.3965%	0.0045%	0.0697%	1.0634%	1.9912%	0.8293%	2.6074%	0.6537%	2.4126%	0.0939%	1.8344%
Trichosphaeriales	0.000	0.000	0.1028%	0.0247%	0.0035%	0.0061%	0.1593%	0.1528%	0.1242%	9.5176%	0.6403%	0.1520%	0.0924%	0.8056%
Sebacinales	0.000	0.000	0.0167%	2.2737%	3.3157%	1.9333%	0.5856%	0.0023%	0.0051%	0.0005%	3.3782%	0.0934%	0.0187%	0.0200%
Spizellomycetales	0.000	0.000	7.9722%	0.0030%	0.0061%	0.0010%	0.0023%	0.0000%	0.0000%	0.0028%	0.0000%	0.0000%	0.0000%	0.0000%
Incertae_sedis	0.000	0.000	0.0736%	0.3646%	0.2394%	5.9717%	0.1065%	0.0111%	0.0051%	0.0324%	0.0542%	0.8354%	0.0010%	0.0167%
Chaetosphaeriales	0.000	0.000	0.0681%	1.4904%	4.2222%	1.1889%	0.0074%	0.0000%	0.0045%	0.0000%	0.0653%	0.0803%	0.1854%	0.0039%
Sordariales	0.000	0.000	5.5028%	0.4859%	0.6273%	0.2283%	0.0130%	0.0000%	0.0000%	0.0356%	0.0144%	0.0066%	0.0091%	0.0050%
unidentified	0.000	0.000	0.6139%	1.0692%	2.2843%	1.2763%	0.3375%	0.3954%	0.4076%	0.2227%	0.1838%	0.0510%	0.0354%	0.0472%
unidentified	0.000	0.000	0.4264%	1.2258%	2.3692%	0.9500%	0.0162%	0.0282%	0.0247%	0.6787%	0.0435%	0.0253%	0.2237%	0.0361%
Tilletiales	0.000	0.000	0.0000%	0.0172%	0.0000%	0.0035%	0.0292%	0.0171%	0.0187%	5.7926%	0.0088%	0.0136%	0.0247%	0.0778%
Incertae_sedis	0.000	0.000	0.0000%	0.0919%	0.0000%	0.0303%	1.4255%	1.0509%	0.8848%	1.4403%	0.1889%	0.1015%	0.0399%	0.0978%
Microascales	0.000	0.000	1.6153%	1.0520%	1.6000%	0.1020%	0.0065%	0.0000%	0.0000%	0.0051%	0.0042%	0.0040%	0.0000%	0.0011%
Dothideales	0.000	0.000	0.0111%	0.1323%	0.0000%	0.0000%	1.1153%	0.3750%	0.0545%	0.8699%	0.6019%	0.0187%	0.0308%	0.2861%
Cantharellales	0.000	0.000	0.0000%	0.3944%	0.4343%	1.5258%	0.0019%	0.0366%	0.0076%	0.0190%	0.0102%	0.9172%	0.0111%	0.0028%
Unkown Dothideomycetes	0.000	0.000	0.1903%	0.1020%	0.1823%	0.4455%	0.0829%	0.1324%	0.3384%	0.5792%	0.0241%	0.0081%	0.0328%	0.1311%
Glomerales	0.000	0.000	0.0931%	0.5823%	0.9975%	0.0485%	0.0083%	0.0000%	0.0005%	0.0005%	0.0005%	0.0005%	0.0076%	0.0028%
Unkown Eurotiomycetes	0.000	0.000	1.2236%	0.0949%	0.0495%	0.0005%	0.0134%	0.0181%	0.0227%	0.0014%	0.0000%	0.0000%	0.0152%	0.0022%
Onygenales	0.000	0.000	1.2486%	0.0374%	0.0000%	0.0000%	0.0000%	0.0000%	0.0000%	0.0000%	0.0000%	0.0000%	0.0000%	0.0000%
Unkown Leotiomycetes	0.000	0.000	1.2208%	0.0071%	0.0000%	0.0000%	0.0000%	0.0000%	0.0000%	0.0102%	0.0000%	0.0000%	0.0000%	0.0000%
unidentified	0.000	0.000	0.3819%	0.0338%	0.0717%	0.4561%	0.0023%	0.0023%	0.0005%	0.0435%	0.0000%	0.0015%	0.0025%	0.0061%
Microstromatales	0.000	0.000	0.0000%	0.0030%	0.0000%	0.0005%	0.1954%	0.3944%	0.0439%	0.2681%	0.0324%	0.0000%	0.0000%	0.0283%
Rhizophlyctidales	0.000	0.000	0.8597%	0.0000%	0.0000%	0.0000%	0.0000%	0.0000%	0.0000%	0.0028%	0.0000%	0.0000%	0.0000%	0.0000%
unidentified	0.000	0.000	0.0083%	0.7798%	0.0197%	0.0247%	0.0000%	0.0000%	0.0000%	0.0000%	0.0000%	0.0000%	0.0000%	0.0000%
Mucorales	0.000	0.000	0.2431%	0.4025%	0.0652%	0.0121%	0.0046%	0.0000%	0.0000%	0.0384%	0.0000%	0.0030%	0.0000%	0.0000%
Xylariales	0.000	0.000	0.0000%	0.0035%	0.0000%	0.0308%	0.0083%	0.0120%	0.0040%	0.1981%	0.3806%	0.0045%	0.0000%	0.0494%
unidentified	0.000	0.000	0.1861%	0.1641%	0.2697%	0.0636%	0.0005%	0.0000%	0.0000%	0.0000%	0.0000%	0.0061%	0.0000%	0.0000%
Unkown	0.000	0.000	0.0569%	0.3788%	0.0444%	0.0131%	0.0028%	0.0005%	0.0318%	0.0315%	0.0032%	0.0025%	0.0136%	0.0017%
Corticiales	0.000	0.000	0.5542%	0.0000%	0.0000%	0.0000%	0.0000%	0.0000%	0.0000%	0.0014%	0.0005%	0.0000%	0.0000%	0.0011%
unidentified	0.000	0.000	0.0861%	0.0364%	0.0015%	0.0470%	0.0005%	0.0102%	0.0000%	0.1051%	0.0333%	0.2273%	0.0000%	0.0044%
Orbiliales	0.000	0.000	0.0167%	0.1005%	0.0495%	0.2581%	0.0231%	0.0000%	0.0000%	0.0000%	0.0954%	0.0020%	0.0000%	0.0000%
Russulales	0.000	0.000	0.0000%	0.0000%	0.0000%	0.0000%	0.0009%	0.0009%	0.0020%	0.2912%	0.0005%	0.0702%	0.0000%	0.0600%
unidentified	0.000	0.000	0.0000%	0.0167%	0.0000%	0.0005%	0.1116%	0.0907%	0.1025%	0.0481%	0.0199%	0.0222%	0.0000%	0.0061%
Botryosphaeriales	0.000	0.000	0.3264%	0.0000%	0.0000%	0.0000%	0.0000%	0.0037%	0.0005%	0.0468%	0.0199%	0.0000%	0.0000%	0.0000%
Phyllachorales	0.000	0.000	0.0000%	0.0000%	0.0025%	0.0010%	0.0000%	0.0745%	0.0000%	0.0366%	0.2454%	0.0000%	0.0000%	0.0056%
Auriculariales	0.000	0.000	0.0167%	0.0975%	0.1212%	0.1071%	0.0000%	0.0000%	0.0040%	0.0060%	0.0000%	0.0000%	0.0096%	0.0000%
unidentified	0.000	0.000	0.0000%	0.1141%	0.0753%	0.1424%	0.0000%	0.0000%	0.0000%	0.0000%	0.0000%	0.0000%	0.0000%	0.0000%
unidentified	0.000	0.000	0.0278%	0.0005%	0.0000%	0.0000%	0.0056%	0.0398%	0.0000%	0.1491%	0.0296%	0.0025%	0.0025%	0.0183%
unidentified	0.000	0.000	0.0000%	0.0086%	0.0000%	0.0000%	0.1324%	0.0426%	0.0121%	0.0000%	0.0264%	0.0288%	0.0000%	0.0000%
Mortierellales	0.000	0.000	0.0875%	0.0515%	0.0025%	0.0278%	0.0125%	0.0074%	0.0000%	0.0000%	0.0167%	0.0000%	0.0000%	0.0000%
Incertae_sedis	0.000	0.000	0.1625%	0.0162%	0.0253%	0.0000%	0.0000%	0.0000%	0.0000%	0.0000%	0.0000%	0.0005%	0.0000%	0.0000%
Malasseziales	0.000	0.000	0.0347%	0.0071%	0.0000%	0.0000%	0.0000%	0.0000%	0.0015%	0.0301%	0.0042%	0.0157%	0.0157%	0.0894%
Unkown Tremellomycetes	0.000	0.000	0.1361%	0.0146%	0.0025%	0.0071%	0.0023%	0.0000%	0.0000%	0.0046%	0.0000%	0.0000%	0.0000%	0.0000%
Rhizophydiales	0.000	0.000	0.1181%	0.0419%	0.0005%	0.0025%	0.0000%	0.0000%	0.0000%	0.0000%	0.0000%	0.0000%	0.0000%	0.0000%
Cystobasidiales	0.000	0.000	0.0069%	0.0015%	0.0000%	0.0000%	0.0528%	0.0458%	0.0146%	0.0069%	0.0051%	0.0197%	0.0000%	0.0000%
Ophiostomatales	0.000	0.000	0.0583%	0.0197%	0.0025%	0.0348%	0.0023%	0.0000%	0.0000%	0.0000%	0.0000%	0.0000%	0.0000%	0.0000%
Unkown	0.000	0.000	0.0917%	0.0091%	0.0000%	0.0000%	0.0000%	0.0000%	0.0000%	0.0000%	0.0000%	0.0000%	0.0000%	0.0000%
unidentified	0.000	0.000	0.0000%	0.0828%	0.0000%	0.0086%	0.0000%	0.0000%	0.0000%	0.0000%	0.0000%	0.0000%	0.0000%	0.0000%
Incertae_sedis	0.000	0.000	0.0417%	0.0202%	0.0061%	0.0015%	0.0005%	0.0000%	0.0000%	0.0088%	0.0000%	0.0010%	0.0000%	0.0100%
Unkown Chytridiomycetes	0.000	0.000	0.0375%	0.0283%	0.0000%	0.0086%	0.0000%	0.0000%	0.0000%	0.0000%	0.0000%	0.0000%	0.0000%	0.0000%
Erythrobasidiales	0.000	0.000	0.0000%	0.0025%	0.0010%	0.0000%	0.0005%	0.0042%	0.0025%	0.0421%	0.0000%	0.0000%	0.0000%	0.0083%
Diversisporales	0.000	0.000	0.0000%	0.0237%	0.0273%	0.0025%	0.0000%	0.0000%	0.0000%	0.0000%	0.0000%	0.0000%	0.0000%	0.0000%
Blastocladiales	0.000	0.000	0.0028%	0.0000%	0.0000%	0.0000%	0.0000%	0.0000%	0.0025%	0.0481%	0.0000%	0.0000%	0.0000%	0.0000%
Unkown	0.000	0.000	0.0000%	0.0146%	0.0111%	0.0263%	0.0000%	0.0000%	0.0000%	0.0000%	0.0000%	0.0000%	0.0000%	0.0000%
Hymenochaetales	0.000	0.000	0.0000%	0.0202%	0.0000%	0.0000%	0.0000%	0.0000%	0.0015%	0.0222%	0.0032%	0.0010%	0.0000%	0.0000%
Kickxellales	0.000	0.000	0.0222%	0.0237%	0.0000%	0.0000%	0.0000%	0.0000%	0.0000%	0.0000%	0.0009%	0.0000%	0.0000%	0.0000%
Incertae_sedis	0.000	0.000	0.0000%	0.0000%	0.0000%	0.0000%	0.0000%	0.0005%	0.0000%	0.0282%	0.0000%	0.0000%	0.0000%	0.0028%
Archaeorhizomycetales	0.000	0.000	0.0278%	0.0000%	0.0000%	0.0000%	0.0000%	0.0000%	0.0000%	0.0000%	0.0000%	0.0000%	0.0000%	0.0000%
Wallemiales	0.000	0.001	0.0028%	0.0025%	0.0000%	0.0000%	0.0000%	0.0000%	0.0020%	0.0102%	0.0019%	0.0000%	0.0000%	0.0000%

Supplementary Table 5 - Identified bacterial genera and its growth-promoting traits. Bacterial genera with representatives described as plant-growth promoters were searched in the online tool Web of Science (http://www.webofknowledge.com) using a query comprised of the genus name amended by "AND ((PGPR) OR (PGPB) OR ("plant growth-promoting") OR ("plant growth-promotion") OR (plant*growth*) OR (plant*associat*))". A query with the genus name amended by "AND ((sugar*cane) OR (Saccharum))" was used to verify if a genus representative was isolated from sugarcane. The source tissue of isolation in sugarcane was informed in cases where a member was isolated from sugarcane and described as beneficial to plant growth. Only publications describing growth-promotion traits through *in vivo* or *in vitro* assays were considered. During the survey, publications were sorted by number of citation and chosen based on their relevance to this work. Whenever possible, at least three publications per genus were cited. Additionally, the authors included publications they considered relevant to the discussion. rz: root zone; st: stalk. lf: leaf; ACC: ACC deaminase; AM: ammonia production; ANT: anti-microbial activity; CK: cytokinin production; EPS: exopolysaccharide production; GA: gibberellin production; HCN: HCN production; HMS: heavy metal solubilization; SID: siderophore production; IAA: indoleacetic acid production; N2: nitrogen fixation; PS: phosphate solubilization. Ref.: reference.

Genus	Growth-promoting trait	Isolated from sugarcane? (organ)	Ref.
Acetobacter	IAA, N2	rt, st, lf	[1–4]
Achromobacter	ACC, AM, EPS, HCN, N2, SID	rt, st	[5–10]
Acidisoma	-	-	-
Acidocella	-	-	-
Acidovorax	AM, IAA,HCN, PS, SID	-	[11,12]
Acinetobacter	ACC, ANT, IAA, N2, PS, SID	st	[13–16]
Actinoallomurus	-	-	-
Actinomadura	IAA	-	[17]
Actinomyces	-	-	-
Actinoplanes	-	-	-
Actinopolymorpha	-	-	-
Afifella	-	-	-
Agrobacterium	ACC, IAA, N2	st	[18–20]
Alicyclobacillus	-	-	-
Ammoniphilus	-	-	-
Amycolatopsis	-	-	-
Aquicella	-	-	-
Arthrospira	-	-	-
Asteroleplasma	-	-	-
Asticcacaulis	-	-	-
Azospirillum	N2	rt	[21,22]

Azovibrio	-	-	-
Bacillus	ACC, AM, ANT, GA, HCN, IAA, PS, SID	rt,st	[16,23–41]
Balneimonas	-	-	-
Bdellovibrio	-	-	-
Beijerinckia	GA, IAA, N2	rt	[42–44]
Blastochloris	-	-	-
Blastomonas	-	-	-
Bosea	IAA, N2	-	[45,46]
Bradyrhizobium	AM, EPS, HMS, HCN, IAA, PS, SID	rz	[47–55]
Burkholderia	ACC, HMS, IAA, N2, PS, SID	rz,st,lf	[38,39,43,56–60]
Catellatospora	-	-	-
Caulobacter	N2	rz	[61,62]
Cellvibrio	-	-	-
Chitinophaga	-	-	-
Chondromyces	-	-	-
Chromobacterium	ANT	-	[63]
Chryseobacterium	PS	-	[64,65]
Chthoniobacter	-	-	-
Cloacibacterium	-	-	-
Cohnella	-	-	-
Collimonas	-	-	-
Corynebacterium	-	-	-
Crocinitomix	-	-	-
Cryocola	-	-	-
Cupriavidus	-	-	-
Curtobacterium	IAA	st	[45,66]
Curvibacter	-	-	-
Cytophaga	-	-	-
DA101	-	-	-
Dactylosporangium	-	-	-
Dechloromonas	-	-	-
Devosia	-	-	-
Dokdonella	-	-	-
Dyadobacter	-	-	-
Dyella	ACC, IAA, PS	-	[67,68]
Edaphobacter	-	-	-
Emticicia	-	-	-
Enhydrobacter	-	-	-
Enterobacter	ACC, AM, EPS, HCN, IAA, N2, PS, SID	rz,st,lf	[38,43,45,61,69–72]
Erwinia	IAA, ACC, PS	-	[16]
Fimbriimonas	-	-	-

Flavisolibacter	-	-	-
Flavobacterium	IAA, N2, SID	lf	[73–75]
Fluviicola	-	-	-
Gemmata	-	-	-
Geobacter	-	-	-
Geodermatophilus	-	-	-
Geothrix	-	-	-
Gluconobacter	N2, IAA	rt, st, lf	[1–3,76–80]
Glycomyces	-	-	-
Gordonia	-	-	-
Haemophilus	-	-	-
Herbaspirillum	N2	st, lf	[81–84]
heteroC45_4W	-	-	-
Hylemonella	-	-	-
Hyphomicrobium	-	-	-
Iamia	-	-	-
Inquilinus	-	-	-
Janthinobacterium	IAA	-	[85]
Kaistia	-	-	-
Kaistobacter	-	-	-
Kibdelosporangium	SID	-	[86]
Klebsiella	IAA, N2, PS	rt, st	[61,70,87,88]
Kribbella	-	-	-
Labrys	-	-	-
Legionella	-	-	-
Leptothrix	-	-	-
Leuconostoc	-	-	-
Luteibacter	-	-	-
Luteimonas	-	-	-
Luteolibacter	-	-	-
Lysobacter	ANT, PS	-	[89,90]
Mesorhizobium	IAA, PS , N2	rt	[23,63,91–93]
Methylibium	-	-	-
Methylobacterium	N2, PS	-	[94,95]
Microbacterium	ACC, AM, EPS, HCN, PS, SID	st	[38]
Mycobacterium	IAA, SID	-	[34,96,97]
Mycoplana	-	-	-
Mycoplasma	-	-	-
Neisseria	-	-	-
Nevskia	-	-	-
Niastella	-	-	-
Nitrospira	-	-	-
Nocardia	-	-	-

Nocardioides	-	-	-
Novosphingobium	-	-	-
Ochrobactrum	-	-	-
Opitutus	-	-	-
OR-59	-	-	-
Paenibacillus	ACC, CK, IAA, SID	rt, st, lf	24,25,70,81,98–100
Pandoraea	-	-	-
Paracoccus	-	-	-
Parvibaculum	-	-	-
Pedobacter	IAA		73
Pedomicrobium	-	-	-
Pedosphaera	-	-	-
Phaeospirillum	-	-	-
Phenylobacterium	-	-	-
Pigmentiphaga	-	-	-
Pilimelia	-	-	-
Pimelobacter	-	-	-
Pirellula	-	-	-
Planctomyces	-	-	-
Plesiocystis	-	-	-
Polaromonas	-	-	-
Pontibacter	-	-	-
Propionibacterium	-	-	-
Prosthecobacter	-	-	-
Pseudomonas	ACC, AM, ANT, CK, EPS, HCN, HMS, IAA, N2, PS, SID	rt, st, lf	24,25,29,30,32,34,38,39,81,92,97,101–106
Pseudonocardia	-	-	-
Pseudoxanthomonas	AM, EPS, HCN, SID	-	9
Ralstonia	-	-	-
Rathayibacter	-	-	-
Rhizobium	AM, CK, EPS, HCN, IAA, PS, SID	rz,st,	25,34,50,53,61,85,92,97,107–112
Rhodoferax	-	-	-
Rhodoplanes	-	-	-
Rickettsia	-	-	-
Rickettsiella	-	-	-
Rubrivivax	-	-	-
Rubrobacter	-	-	-
Saccharopolyspora	-	-	-
Salinibacterium	-	-	-
Salinispora	-	-	-
Sediminibacterium	-	-	-

Serratia	IAA, ANT, GA, HCN, PS	-	[113,114]
Shinella	-	-	-
Solirubrobacter	-	-	-
Sphingobacterium	IAA, HCN	-	[115]
Sphingobium	-	-	-
Sphingomonas	IAA, N2	rt, st, lf	[8,34,38]
Sphingopyxis	-	-	-
Spirochaeta	-	-	-
Spirosoma	-	-	-
Sporocytophaga	-	-	-
Sporosarcina	PS, SID	-	[116]
Staphylococcus	-	-	-
Stenotrophomonas	-	-	-
Steroidobacter	-	-	-
Streptococcus	-	-	-
Streptomyces	ACC, SID, IAA	rz	[38,117]
Swaminathania	-	-	-
Telmatospirillum	-	-	-
Terriglobus	-	-	-
Thermomonas	-	-	-
Turneriella	-	-	-
Uliginosibacterium	-	-	-
Variovorax	IAA, SID	-	[74]
Wolbachia	-	-	-
Yonghaparkia	-	-	-

Reference for Supplementary Table 5

1. James, E. K., Reis, V. M., Olivares, F. L., Baldani, J. I. & Döbereiner, J. Infection of sugar cane by the nitrogen-fixing bacterium Acetobacter diazotrophicus. *J. Exp. Bot.* **45,** 757–766 (1994).

2. GILLIS, M. *et al.* Acetobacter diazotrophicus sp. nov., a Nitrogen-Fixing Acetic Acid Bacterium Associated with Sugarcane. *Int. J. Syst. Bacteriol.* **39,** 361–364 (1989).

3. Fuentes-Ramirez, L. E., Jimenez-Salgado, T., Abarca-Ocampo, I. R. & Caballero-Mellado, J. Acetobacter diazotrophicus, an indoleacetic acid producing bacterium isolated from sugarcane cultivars of Mexico. *Plant Soil* **154,** 145–150 (1993).

4. Boddey, R. M. *et al.* Biological nitrogen fixation associated with sugar cane and rice: Contributions and prospects for improvement. *Plant Soil* **174,** 195–209 (1995).

5. Mayak, S., Tirosh, T. & Glick, B. R. Plant growth-promoting bacteria confer resistance in tomato plants to salt stress. *Plant Physiol. Biochem.* **42,** 565–572 (2004).

6. Mayak, S., Tirosh, T. & Glick, B. R. Plant growth-promoting bacteria that confer resistance to water stress in tomatoes and peppers. *Plant Sci.* **166,** 525–530 (2004).

7. Ma, Y., Rajkumar, M. & Freitas, H. Inoculation of plant growth promoting bacterium Achromobacter xylosoxidans strain Ax10 for the improvement of copper phytoextraction by Brassica juncea. *J. Environ. Manage.* **90,** 831–837 (2009).

8. Castanheira, N. *et al.* Annual ryegrass-associated bacteria with potential for plant growth promotion. *Microbiol. Res.* **169,** 768–779 (2014).

9. Zhou, G. *et al.* The metabolism of neonicotinoid insecticide thiamethoxam by soil enrichment cultures, and the bacterial diversity and plant growth-promoting properties of the cultured isolates. *J. Environ. Sci. Heal. Part B* **49,** 381–390 (2014).

10. Weingarden, A. *et al.* Dynamic changes in short- and long-term bacterial composition following fecal microbiota transplantation for recurrent Clostridium difficile infection. *Microbiome* **3,** 1–8 (2015).

11. Peiffer, J. a *et al.* Diversity and heritability of the maize rhizosphere microbiome under field conditions. *Proc. Natl. Acad. Sci. U. S. A.* **110,** 6548–53 (2013).

12. Li, D. *et al.* Phenotypic variation in Acidovorax radicis N35 influences plant growth promotion. *FEMS Microbiol. Ecol.* **79,** 751–762 (2012).

13. Van Overbeek, L. & Van Elsas, J. D. Effects of plant genotype and growth stage on the structure of bacterial communities associated with potato (Solanum tuberosum L.). *FEMS Microbiol. Ecol.* **64,** 283–296 (2008).

14. Indiragandhi, P., Anandham, R., Madhaiyan, M. & Sa, T. M. Characterization of Plant Growth–Promoting Traits of Bacteria Isolated from Larval Guts of Diamondback Moth Plutella xylostella (Lepidoptera: Plutellidae). *Curr. Microbiol.* **56,** 327–333 (2008).

15. Farokh, R. Z. *et al.* Characterization of plant-growth-promoting traits of Acinetobacter species isolated from rhizosphere of Pennisetum glaucum. *J. Microbiol. Biotechnol.* **21,** 556–566 (2011).

16. Velázquez, E. *et al.* Genetic diversity of endophytic bacteria which could be find in the apoplastic sap of the medullary parenchym of the stem of healthy sugarcane plants. *J. Basic Microbiol.* **48,** 118–124 (2008).

17. Madhurama, G., Sonam, D., Urmil, P. G. & Ravindra, N. K. Diversity and biopotential of endophytic actinomycetes from three medicinal plants in India. *African J. Microbiol. Res.* **8,** 184–191 (2014).

18. Xing, Y.-X., Yang, L.-T., Huang, S.-L. & Li, Y.-R. Identification of a new nitrogen fixing endo-bacterium strain isolated from sugarcane stalk. *Sugar Tech* **8,** 49–53 (2006).

19. Blaha, D., Prigent-Combaret, C., Mirza, M. S. & MoÃ«nne-Loccoz, Y. Phylogeny of the 1-aminocyclopropane-1-carboxylic acid deaminase-encoding gene acdS in phytobeneficial and pathogenic Proteobacteria and relation with strain biogeography. *FEMS Microbiol. Ecol.* **56,** 455–470 (2006).

20. Hao, X. *et al.* Genome Sequence and Mutational Analysis of Plant-Growth-Promoting Bacterium Agrobacterium tumefaciens CCNWGS0286 Isolated from a Zinc-Lead Mine Tailing. *Appl. Environ. Microbiol.* **78,** 5384–5394 (2012).

21. Magalhaes, F. M., Baldani, J. I., Souto, S. M. & Kuykendall, J. R. A new acid tolerant Azospirillum species. *Anais. Acad. bras. Cienc* **55,** 417–430 (1983).

22. James, E. K. Nitrogen fixation in endophytic and associative symbiosis. *F. Crop. Res.* **65,** 197–209 (2000).

23. Ahmad, F., Ahmad, I. & Khan, M. S. Screening of free-living rhizospheric bacteria for their multiple plant growth promoting activities. *Microbiol. Res.* **163,** 173–181 (2008).

24. Beneduzi, A., Peres, D., Vargas, L. K., Bodanese-Zanettini, M. H. & Passaglia, L. M. P. Evaluation of genetic diversity and plant growth promoting activities of nitrogen-fixing bacilli isolated from rice fields in South Brazil. *Appl. Soil Ecol.* **39,** 311–320 (2008).

25. Çakmakçi, R., Erat, M., Erdoğan, Ü. & Dönmez, M. F. The influence of plant growth-promoting rhizobacteria on growth and enzyme activities in wheat and spinach plants. *J. Plant Nutr. Soil Sci.* **170,** 288–295 (2007).

26. Canbolat, M. Y., Bilen, S., Çakmakçı, R., Şahin, F. & Aydın, A. Effect of plant growth-promoting bacteria and soil compaction on barley seedling growth, nutrient uptake, soil properties and rhizosphere microflora. *Biol. Fertil. Soils* **42,** 350–357 (2006).

27. Cazorla, F. M. *et al.* Isolation and characterization of antagonistic Bacillus subtilis strains from the avocado rhizoplane displaying biocontrol activity. *J. Appl. Microbiol.* **103,** 1950–1959 (2007).

28. Gutiérrez-Mañero, F. J. *et al.* The plant-growth-promoting rhizobacteria Bacillus pumilus and Bacillus licheniformis produce high amounts of physiologically active gibberellins. *Physiol. Plant.* **111,** 206–211 (2001).

29. Joseph, B., Patra, R. R. & Lawrence, R. Characterization of plant growth promoting rhizobacteria associated with chickpea (Cicer arietinum L .). *Int. J. Plant Prod.* **1,** 141–151 (2007).

30. Rajkumar, M., Nagendran, R., Lee, K. J., Lee, W. H. & Kim, S. Z. Influence of plant growth promoting bacteria and Cr6+ on the growth of Indian mustard. *Chemosphere* **62,** 741–748 (2006).

31. Sheng, X. F. & Xia, J. J. Improvement of rape (Brassica napus) plant growth and cadmium uptake by cadmium-resistant bacteria. *Chemosphere* **64,** 1036–1042 (2006).

32. Tank, N. & Saraf, M. Phosphate solubilization, exopolysaccharide production and indole acetic acid secretion by rhizobacteria isolated from Trigonella foenum-graecum. *Indian J. Microbiol.* **43,** 37–40 (2003).

33. Thakuria, D. *et al.* Characterization and screening of bacteria from rhizosphere of rice grown in acidic soils of Assam. *Curr. Sci.* **86,** 978–985 (2004).

34. Tsavkelova, E. a., Cherdyntseva, T. a. & Netrusov, a. I. Auxin production by bacteria associated with orchid roots. *Microbiology* **74,** 46–53 (2005).

35. Wani, P. A. & Khan, M. S. Bacillus species enhance growth parameters of chickpea (Cicer arietinum L.) in chromium stressed soils. *Food Chem. Toxicol.* **48,** 3262–3267 (2010).

36. Yasmin, S., Bakar, M. A. R., Malik, K. a. & Hafeez, F. Y. Isolation, characterization and beneficial effects of rice associated plant growth promoting bacteria from Zanzibar soils. *J. Basic Microbiol.* **44,** 241–252 (2004).

37. Zaidi, S., Usmani, S., Singh, B. R. & Musarrat, J. Significance of Bacillus subtilis strain SJ-101 as a bioinoculant for concurrent plant growth promotion and nickel accumulation in Brassica juncea. *Chemosphere* **64,** 991–997 (2006).

38. Beneduzi, A. *et al.* Diversity and plant growth promoting evaluation abilities of bacteria isolated from sugarcane cultivated in the South of Brazil. *Appl. Soil Ecol.* **63,** 94–104 (2013).

39. Antwerpen, T. V. a N. *et al.* Assessment of Sugarcane Endophytic Bacteria and Rhizospheric Burkholderia Species As Antifungal Agents. *Proc. South African Sugar Technol. Assoc.* **76,** 301–304 (2002).

40. Gangwar, M. & Kaur, G. Isolation and characterization of endophytic bacteria from endorhizosphere of sugarcane and ryegrass. *Internet J. Microbiol.* **7,** 139–144 (2008).

41. Hassan, M. N., Afghan, S. & Hafeez, F. Y. Suppression of red rot caused by Colletotrichum falcatum on sugarcane plants using plant growth-promoting rhizobacteria. *BioControl* **55,** 531–542 (2010).

42. Döbereiner, J., Day, J. M. & Dart, P. J. Nitrogenase activity in the rhizosphere of sugarcane and some other tropical grasses. *Plant Soil* **196,** 191–196 (1972).

43. De Oliveira, Z. M., Floh, E. I. S., Ferrara, F. I. S. & Barbosa, H. R. Diazotrophyc rhizobacteria isolated from sugarcane can release amino acids in a synthetic culture medium. *Biol. Fertil. Soils* **47,** 957–962 (2011).

44. Alagawadi, A. R., Ammanna, S., Doddagoudar, C. K., Marihal, A. & Krishnaraj, P. U. Native Isolates of Beijerinckia from Western Ghats Producing High Amount of Indole Acetic Acid. *J. Pure Appl. Microbiol.* **8,** 4221–4224 (2014).

45. Magnani, G. S. *et al.* Diversity of endophytic bacteria in Brazilian sugarcane. *Genet. Mol. Res.* **9,** 250–258 (2010).

46. Lee, S., Lee, S., Seul, K., Park, S. & Ghim, S. Plant growth-promoting capabilities of diazotrophs from wild gramineous crops. *Korean J. Microbiol. Biotechnol.* **34,** 78 (2006).

47. Abd-Alla, M. H. Solubilization of rock phosphates by Rhizobium and Bradyrhizobium. *Folia Microbiol. (Praha).* **39,** 53–56 (1994).

48. Antoun, H., Beauchamp, C. J., Goussard, N., Chabot, R. & Lalande, R. Potential of *Rhizobium* and *Bradyrhizobium* species as plant growth promoting rhizobacteria on non-legumes: effect on radishes (*Raphanus sativus* L.). *Plant Soil* **204,** 57–67 (1998).

49. Deshwal, V. K., Pandey, P., Kang, S. C. & Maheshwari, D. K. Rhizobia as a biological control agent against soil borne plant pathogenic fungi. *Indian J. Exp. Biol.* **41,** 1160–1164 (2003).

50. Duhan, J. S., Dudeja, S. S. & Khurana, a. L. Siderophore production in relation to N2 fixation and iron uptake in pigeon pea-Rhizobium symbiosis. *Folia Microbiol. (Praha).* **43,** 421–426 (1998).

51. Dary, M., Chamber-Pérez, M. a., Palomares, a. J. & Pajuelo, E. 'In situ' phytostabilisation of heavy metal polluted soils using Lupinus luteus inoculated with metal resistant plant-growth promoting rhizobacteria. *J. Hazard. Mater.* **177,** 323–330 (2010).

52. Khan, M. S., Zaidi, A. & Aamil, M. Biocontrol of fungal pathogens by the use of plant growth promoting rhizobacteria and nitrogen fixing microorganisms. *Indian J Bot Soc* **81,** 255–263 (2002).

53. Shaharoona, B., Arshad, M. & Zahir, Z. a. Effect of plant growth promoting rhizobacteria containing ACC-deaminase on maize (Zea mays L.) growth under axenic conditions and on nodulation in mung bean (Vigna radiata L.). *Lett. Appl. Microbiol.* **42,** 155–159 (2006).

54. Wani, P. A., Khan, M. S. & Zaidi, A. Effect of metal tolerant plant growth promoting Bradyrhizobium sp. (vigna) on growth, symbiosis, seed yield and metal uptake by greengram plants. *Chemosphere* **70,** 36–45 (2007).

55. Wittenberg, J. B., Wittenberg, B. a., Day, D. a., Udvardi, M. K. & Appleby, C. a. Siderophore-bound iron in the peribacteriod space of soybean root nodules. *Plant Soil* **178,** 161–169 (1996).

56. Caballero-Mellado, J., Martínez-Aguilar, L., Paredes-Valdez, G. & Estrada-de los Santos, P. Burkholderia unamae sp. nov., an N2-fixing rhizospheric and endophytic species. *Int. J. Syst. Evol. Microbiol.* **54,** 1165–1172 (2004).

57. Luvizotto, D. M. *et al.* Genetic diversity and plant-growth related features of Burkholderia spp. from sugarcane roots. *World J. Microbiol. Biotechnol.* **26,** 1829–1836 (2010).

58. Perin, L. *et al.* Burkholderia silvatlantica sp. nov., a diazotrophic bacterium associated with sugar cane and maize. *Int. J. Syst. Evol. Microbiol.* **56,** 1931–1937 (2006).

59. Yu, X., Liu, X., Zhu, T. H., Liu, G. H. & Mao, C. Co-inoculation with phosphate-solubilzing and nitrogen-fixing bacteria on solubilization of rock phosphate and their effect on growth promotion and nutrient uptake by walnut. *Eur. J. Soil Biol.* **50,** 112–117 (2012).

60. Stoyanova, M., Pavlina, I., Moncheva, P. & Bogatzevska, N. Biodiversity and incidence of Burkholderia species. *Biotechnol. Biotechnol. Equip.* **21,** 306–310 (2007).

61. Mehnaz, S., Baig, D. N. & Lazarovits, G. Genetic and phenotypic diversity of plant growth promoting rhizobacteria isolated from sugarcane plants growing in Pakistan. *J. Microbiol. Biotechnol.* **20,** 1614–1623 (2010).

62. Pepe, O., Ventorino, V. & Blaiotta, G. Dynamic of functional microbial groups during mesophilic composting of agro-industrial wastes and free-living (N2)-fixing bacteria application. *Waste Manag.* **33,** 1616–1625 (2013).

63. Idris, H. A., Labuschagne, N. & Korsten, L. Screening rhizobacteria for biological control of Fusarium root and crown rot of sorghum in Ethiopia. *Biol. Control* **40,** 97–106 (2007).

64. Singh, A. V., Chandra, R. & Goel, R. Phosphate solubilization by Chryseobacterium sp. and their combined effect with N and P fertilizers on plant growth promotion. *Arch. Agron. Soil Sci.* **59,** 641–651 (2012).

65. Chen, Y. P. *et al.* Phosphate solubilizing bacteria from subtropical soil and their tricalcium phosphate solubilizing abilities. *Appl. Soil Ecol.* **34,** 33–41 (2006).

66. De Pereira, G. V. M., Magalhães, K. T., Lorenzetii, E. R., Souza, T. P. & Schwan, R. F. A Multiphasic Approach for the Identification of Endophytic Bacterial in Strawberry Fruit and their Potential for Plant Growth Promotion. *Microb. Ecol.* **63,** 405–417 (2012).

67. Palaniappan, P., Chauhan, P. S., Saravanan, V. S., Anandham, R. & Sa, T. Isolation and characterization of plant growth promoting endophytic bacterial isolates from root nodule of Lespedeza sp. *Biol. Fertil. Soils* **46,** 807–816 (2010).

68. Anandham, R., Indira Gandhi, P., Madhaiyan, M. & Sa, T. Potential plant growth promoting traits and bioacidulation of rock phosphate by thiosulfate oxidizing bacteria isolated from crop plants. *J. Basic Microbiol.* **48,** 439–447 (2008).

69. Graciolli, L. A., de Freitas, J. R. & Ruschel, A. P. Bactérias fixadoras de nitrogênio nas raízes, caules e folhas de cana-de-açúcar (Saccharum sp.). *Rev. Microbiol.* **14,** 191–196 (1983).

70. Mirza, M. S. *et al.* Isolation, partial characterization, and the effect of plant-promoting bacteria (PGPB) on micro-propagated sugarcane in vitro. *Plant Soil* **237,** 47–54 (2001).

71. Rennie, R. J., Ruschel, A. P., Vose, P. B. & Freitas, J. R. Isolation and identification of N2-fixing bacteria associated with sugar cane (Saccharum sp.). *Can. J. Microbiol.* **28,** 462–467 (1982).

72. Li, R. & Rae, I. C. Mac. Isolation and identification of N2-fixing enterobacteria associated with sugarcane in tropical Australia. *J. Gen. Appl. Microbiol.* **38,** 523–531 (1992).

73. Yuan, C.-L., Mou, C.-X., Wu, W.-L. & Guo, Y.-B. Effect of different fertilization treatments on indole-3-acetic acid producing bacteria in soil. *J. Soils Sediments* **11,** 322–329 (2011).

74. Belimov, a. a. *et al.* Cadmium-tolerant plant growth-promoting bacteria associated with the roots of Indian mustard (Brassica juncea L. Czern.). *Soil Biol. Biochem.* **37,** 241–250 (2005).

75. Fischer, G., Jena, V., Pati, B. R. & Chandra, A. K. Diazotrophic Bacterial Population and other Associated Organisms on the Phyllosphere of some Crop Plants Diazotrophe Bakterienpopulation und andere assoziative Organismen aus der. *Zentralbl. Mikrobiol.* **148,** 392–402 (1993).

76. Li, R. & MacRae, I. C. Specific identification and enumeration of Acetobacter diazotrophicus in sugarcane. *Soil Biol. Biochem.* **24,** 413–419 (1992).

77. Sevilla, M., Burris, R. H., Gunapala, N. & Kennedy, C. Comparison of benefit to sugarcane plant growth and 15N2 incorporation following inoculation of sterile plants with Acetobacter diazotrophicus wild-type and Nif- mutants strains. *Mol. Plant. Microbe. Interact.* **14,** 358–366 (2001).

78. Bastián, F. *et al.* Production of indole-3-acetic acid and gibberellins A1 and A3 by Acetobacter diazotrophicus and Herbaspirillum seropedicae in chemically-defined culture media. *Plant Growth Regul.* **24,** 7–11 (1998).

79. Cavalcante, V. a. & Dobereiner, J. A new acid-tolerant nitrogen-fixing bacterium associated with sugarcane. *Plant Soil* **108,** 23–31 (1988).

80. Cavalcante, V. a. & Döbereiner, J. A new acid-tolerant nitrogen-fixing bacterium associated with sugarcane. *Plant Soil* **108,** 23–31 (1988).

81. Pimentel, J. P. *et al.* Dinitrogen fixation and infection of grass leaves by Pseudomonas rubrisubalbicans and Herbaspirillum seropedicae. *Plant Soil* **137,** 61–65 (1991).

82. Asis, C. A. J. *et al.* Endophytic Bacterial Population in Philippine Sugarcane Cultivars and Isolation of Nitrogen-fixing Strains. *Microbes Environ.* **15,** 209–216 (2000).

83. Olivares, F. L., Baldani, V. L. D., Reis, V. M., Baldani, J. I. & D�bereiner, J. Occurrence of the endophytic diazotrophs Herbaspirillum spp. in roots, stems, and leaves, predominantly of Gramineae. *Biol. Fertil. Soils* **21,** 197–200 (1996).

84. Baldani, J. I. *et al.* Emended description of Herbaspirillum; inclusion of [Pseudomonas] rubrisubalbicans, a milk plant pathogen, as Herbaspirillum rubrisubalbicans comb. nov.; and classification of a group of clinical isolates (EF group 1) as Herbaspirillum species 3. *Int. J. Syst. Bacteriol.* **46,** 802–810 (1996).

85. Kuffner, M., Puschenreiter, M., Wieshammer, G., Gorfer, M. & Sessitsch, A. Rhizosphere bacteria affect growth and metal uptake of heavy metal accumulating willows. *Plant Soil* **304,** 35–44 (2008).

86. Qin, S. *et al.* Biodiversity and plant growth promoting traits of culturable endophytic actinobacteria associated with Jatropha curcas L. growing in Panxi dry-hot valley soil. *Appl. Soil Ecol.* **93,** 47–55 (2015).

87. Govindarajan, M., Kwon, S. W. & Weon, H. Y. Isolation, molecular characterization and growth-promoting activities of endophytic sugarcane diazotroph Klebsiella sp. GR9. *World J. Microbiol. Biotechnol.* **23,** 997–1006 (2007).

88. Burd, G. I., Dixon, D. G. & Glick, B. R. Plant growth-promoting bacteria that decrease heavy metal toxicity in plants. *Can. J. Microbiol.* **46,** 237–245 (2000).

89. Hayat, R., Sheirdil, R. A., Iftikhar-ul-Hassan, M. & Ahmed, I. Characterization and identification of compost bacteria based on 16S rRNA gene sequencing. *Ann. Microbiol.* **63,** 905–912 (2012).

90. Rameshkumar, N., Ayyadurai, N., Kayalvizhi, N. & Gunasekaran, P. Genotypic and phenotypic diversity of PGPR fluorescent pseudomonads isolated from the rhizosphere of sugarcane (Saccharum officinarum L.). *J. Microbiol. Biotechnol.* **22,** 13–24 (2012).

91. Kaneko, T. *et al.* Complete genome structure of the nitrogen-fixing symbiotic bacterium Mesorhizobium loti. *DNA Res.* **7,** 331–338 (2000).

92. Wani, P. a., Khan, M. S. & Zaidi, A. Synergistic effects of the inoculation with nitrogen-fixing and phosphate-solubilizing rhizobacteria on the performance of field-grown chickpea. *J. Plant Nutr. Soil Sci.* **170,** 283–287 (2007).

93. Pisa, G. *et al.* Diversity of 16S rRNA genes from bacteria of sugarcane rhizosphere soil. *Brazilian J. Med. Biol. Res.* **44,** 1215–1221 (2011).

94. Ventorino, V. *et al.* Methylobacterium populi VP2: Plant Growth-Promoting Bacterium Isolated from a Highly Polluted Environment for Polycyclic Aromatic Hydrocarbon (PAH) Biodegradation. *Sci. World J.* **2014,** (2014).

95. Madhaiyan, M. *et al.* Effect of co-inoculation of methylotrophic Methylobacterium oryzae with Azospirillum brasilense and Burkholderia pyrrocinia on the growth and nutrient uptake of tomato, red pepper and rice. *Plant Soil* **328,** 71–82 (2010).

96. Egamberdiyeva, D. The effect of plant growth promoting bacteria on growth and nutrient uptake of maize in two different soils. *Appl. Soil Ecol.* **36,** 184–189 (2007).

97. Dell'Amico, E., Cavalca, L. & Andreoni, V. Improvement of Brassica napus growth under cadmium stress by cadmium-resistant rhizobacteria. *Soil Biol. Biochem.* **40,** 74–84 (2008).

98. Phi, Q.-T. *et al.* Assessment of root-associated paenibacillus polymyxa groups on growth promotion and induced systemic resistance in pepper. *J. Microbiol. Biotechnol.* **20,** 1605–1613 (2010).

99. Timmusk, S., Nicander, B., Granhall, U. & Tillberg, E. Cytokinin production by Paenibacillus polymyxa. *Soil Biol. Biochem.* **31,** 1847–1852 (1999).

100. Bal, H. B., Das, S., Dangar, T. K. & Adhya, T. K. ACC deaminase and IAA producing growth promoting bacteria from the rhizosphere soil of tropical rice plants. *J. Basic Microbiol.* **53,** 972–984 (2013).

101. Pandey, A., Trivedi, P., Kumar, B. & Palni, L. M. S. Characterization of a phosphate solubilizing and antagonistic strain of Pseudomonas putida (B0) isolated from a sub-alpine location in the Indian Central Himalaya. *Curr. Microbiol.* **53,** 102–107 (2006).

102. Ahemad, M. & Khan, M. S. Pseudomonas aeruginosa strain PS1 enhances growth parameters of greengram [Vignaradiata (L.) Wilczek] in insecticide-stressed soils. *J. Pest Sci. (2004).* **84,** 123–131 (2011).

103. Ahemad, M. & Khan, M. S. Effect of fungicides on plant growth promoting activities of phosphate solubilizing Pseudomonas putida isolated from mustard (Brassica compestris) rhizosphere. *Chemosphere* **86,** 945–950 (2012).

104. Mehnaz, S. *et al.* Isolation, characterization, and effect of fluorescent pseudomonads on micropropagated sugarcane. *Can. J. Microbiol.* **55,** 1007–1011 (2009).

105. Tripathi, M., Munot, H. P., Shouche, Y., Meyer, J. M. & Goel, R. Isolation and functional characterization of siderophore-producing lead- and cadmium-resistant Pseudomonas putida KNP9. *Curr. Microbiol.* **50,** 233–237 (2005).

106. Mehnaz, S., Baig, D. N., Jamil, F., Weselowski, B. & Lazarovits, G. Characterization of a phenazine and hexanoyl homoserine lactone producing Pseudomonas aurantiaca strain PB-St2, isolated from sugarcane stem. *J. Microbiol. Biotechnol.* **19,** 1688–1694 (2009).

107. Ahemad, M. & Khan, M. S. Effect of tebuconazole-tolerant and plant growth promoting Rhizobium isolate MRP1 on pea-Rhizobium symbiosis. *Sci. Hortic. (Amsterdam).* **129,** 266–272 (2011).

108. Noel, T. C., Sheng, C., Yost, C. K., Pharis, R. P. & Hynes, M. F. Rhizobium leguminosarum as a plant growth-promoting rhizobacterium: direct growth promotion of canola and lettuce. *Can. J. Microbiol.* **42,** 279–283 (1996).

109. Lugtenberg, B. & Kamilova, F. Plant-growth-promoting rhizobacteria. *Annu. Rev. Microbiol.* **63,** 541–556 (2009).

110. Mendes, R. *et al.* Deciphering the rhizosphere microbiome for disease-suppressive bacteria. *Science* **332,** 1097–1100 (2011).

111. Biswas, J. C., Ladha, J. K., Dazzo, F. B., Yanni, Y. G. & Rolfe, B. G. Rhizobial inoculation influences seedling vigor and yield of rice. *Agron. J.* **92,** 880–886 (2000).

112. Taulé, C. *et al.* The contribution of nitrogen fixation to sugarcane (Saccharum officinarum L.), and the identification and characterization of part of the associated diazotrophic bacterial community. *Plant Soil* **356,** 35–49 (2012).

113. Kang, S.-M. *et al.* Gibberellin-producing Serratia nematodiphila PEJ1011 ameliorates low temperature stress in Capsicum annuum L. *Eur. J. Soil Biol.* **68,** 85–93 (2015).

114. Dinesh, R. *et al.* Isolation, characterization, and evaluation of multi-trait plant growth promoting rhizobacteria for their growth promoting and disease suppressing effects on ginger. *Microbiol. Res.* **173,** 34–43 (2015).

115. Marques, A. P. G. C., Pires, C., Moreira, H., Rangel, A. O. S. S. & Castro, P. M. L. Assessment of the plant growth promotion abilities of six bacterial isolates using Zea mays as indicator plant. *Soil Biol. Biochem.* **42,** 1229–1235 (2010).

116. sona Janarthine, S. R., Eganathan, P. Balasubramanian, T. & Vijayalakshmi, S. Endophytic bacteria isolated from the pneumatophores of Avicennia marina. *African J. Microbiol. Res.* **5,** 4455–4466 (2011).

117. Dimkpa, C., Svatos, A., Merten, D., Büchel, G. & Kothe, E. Hydroxamate siderophores produced by Streptomyces acidiscabies E13 bind nickel and promote growth in cowpea (Vigna unguiculata L.) under nickel stress. *Can. J. Microbiol.* **54,** 163–172 (2008).

Supplementary Table 6 - Identified fungal genera and its growth-promoting traits. Fungal genera with representatives described as plant growth promoters were searched in the online tool Web of Science (http://www.webofknowledge.com) using a query comprised of the genus name amended by “AND ((PGPR) OR (PGPB) OR ("plant growth-promoting") OR ("plant growth-promotion") OR (plant*growth*) OR (plant*associat*))”. A query with the genus name amended by “AND ((sugar*cane) OR (Saccharum))” was used to search whether a genus representative was isolated from sugarcane. The source tissue of isolation in sugarcane was informed in cases where a member was isolated from sugarcane and described as beneficial to plant growth. Only publications describing growth-promoting traits through *in vivo* or *in vitro* assays were considered. During the survey, publications were sorted by number of citation and chosen based on their relevance to this work. Whenever possible, at least three publications per genus were cited. Additionally, the authors included publications they considered relevant to the discussion. rz: root zone; st: stalk; lf: leaf; ANT: anti-microbial activity; AST: abiotic/biotic stress tolerance; EDP: enhanced defense against pathogens; GA: gibberellin production; IAA: indoleacetic acid production; N2: nitrogen fixation; PG: plant growth (publications that evaluated growth-promoting effects but did not describe the responsible trait); SID: siderophore production. PS: phosphate solubilization. Ref.: reference.

Genus	Growth promoting trait	Isolated from sugarcane? (organ)	Ref.
Acremonium	ANT, PG	-	[1,2]
Alternaria	EDP	-	[3]
Aporospora	-	-	-
Aspergillus	IAA, GA, PG, PS	-	[4–7]
Aureobasidium	-	-	-
Bipolaris	-	-	-
Bullera	-	-	-
Candida	IAA, ACC, PS	-	[8,9]
Capnobotryella	-	-	-
Capnodium	-	-	-
Cladophialophora	-	-	-
Cladosporium+AD90:AD117	-	-	-
Cladosporium	GA	-	[10]
Clitopilus	-	-	-
Cochliobolus	PG	-	[11]
Codinaeopsis	-	-	-

Colletotrichum	AST	-	12
Coniothyrium	-	-	-
Cryptococcus	PG, IAA, N2	lf	9,13,14
Curvularia	-	-	-
Cytospora	-	-	-
Dactylella	-	-	-
Debaryomyces	-	-	-
Devriesia	-	-	-
Dictyosporium	-	-	-
Didymella	-	-	-
Dissoconium	-	-	-
Epicoccum	ANT, PG	lf	15,16
Erythrobasidium	-	-	-
Exophiala	AST	-	17
Fusarium	PG, GA	-	18,19
Gibberella	GA	-	19,20
Glomus	PG, PS	rt	21,22
Gongronella	-	-	-
Hanseniaspora	-	-	-
Jaminaea	-	-	-
Khuskia	-	-	-
Lasiodiplodia	-	-	-
Leptosphaeria	-	-	-
Leptoxyphium	-	-	-
Magnaporthe	-	-	-
Malassezia	-	-	-
Mariannaea	-	-	-
Meira	-	-	-
Meyerozyma	-	-	-
Microdiplodia	-	-	-
Mycenella	-	-	-
Mycosphaerella	-	-	-
Myrmecridium	-	-	-
Neurospora	-	-	-
Nigrospora	-	-	-
Occultifur	-	-	-
Paecilomyces	PG, AST	-	23
Paraconiothyrium	-	-	-

Paraphaeosphaeria	-	-	-
Passalora	-	-	-
Penicillium	AST, PS, GA, PG	-	[19,24–27]
Penidiella	-	-	-
Periconia	-	-	-
Pestalotiopsis	-	-	-
Phaeosphaeria	-	-	-
Phaeosphaeriopsis	-	-	-
Phialophora	-	-	-
Phoma	ANT,GA, PG		[28–31]
Piriformospora	-	-	-
Pseudocercospora	-	-	-
Ramichloridium	-	-	-
Resinicium	-	-	-
Rhinocladiella	-	-	-
Rhizophagus	AST,PG, PS		[32–34]
Scytalidium	-	-	-
Serendipita	-	-	-
Sporisorium	-	-	-
Strelitziana	-	-	-
Talaromyces	PG	-	[35]
Teratosphaeria	-	-	-
Thielaviopsis	-	-	-
Tilletia	-	-	-
Toxicocladosporium	-	-	-
Trechispora	-	-	-
Tremella	-	-	-
Trichoderma	ANT, PG, PS,	-	[36–40]
Ustilago	-	-	-
Wickerhamiella	-	-	-
Xylaria	-	-	-
Zygoascus	-	-	-
Zygosaccharomyces	-	-	-

Supplementary reference for supplementary table 6

1. Chen, X. M. *et al.* Diversity and Antimicrobial and Plant-Growth-Promoting Activities of Endophytic Fungi in Dendrobium loddigesii Rolfe. *J. Plant Growth Regul.* **29,** 328–337 (2010).

2. Silva, R. L. de O., Luz, J. S., Silveira, E. B. da & Cavalcante, U. M. T. Fungos endofíticos em Annona spp.: isolamento, caracterização enzimática e promoção do crescimento em mudas de pinha (Annona squamosa L.). *Acta Bot. Brasilica* **20,** 649–655 (2006).

3. Van der Ent, S., Van Wees, S. C. M. & Pieterse, C. M. J. Jasmonate signaling in plant interactions with resistance-inducing beneficial microbes. *Phytochemistry* **70,** 1581–1588 (2009).

4. Salas-Marina, M. A. *et al.* The plant growth-promoting fungus Aspergillus ustus promotes growth and induces resistance against different lifestyle pathogens in Arabidopsis thaliana. *J. Microbiol. Biotechnol.* **21,** 686–696 (2011).

5. Hamdali, H., Hafidi, M., Virolle, M. J. & Ouhdouch, Y. Rock phosphate-solubilizing Actinomycetes: screening for plant growth-promoting activities. *World J. Microbiol. Biotechnol.* **24,** 2565–2575 (2008).

6. Khan, A. L. *et al.* Gibberellins producing endophytic Aspergillus fumigatus sp. LH02 influenced endogenous phytohormonal levels, isoflavonoids production and plant growth in salinity stress. *Process Biochem.* **46,** 440–447 (2011).

7. Chuang, C.-C., Kuo, Y.-L., Chao, C.-C. & Chao, W.-L. Solubilization of inorganic phosphates and plant growth promotion by Aspergillus niger. *Biol. Fertil. Soils* **43,** 575–584 (2007).

8. Amprayn, K. *et al.* Plant growth promoting characteristics of soil yeast (Candida tropicalis HY) and its effectiveness for promoting rice growth. *Appl. Soil Ecol.* **61,** 295–299 (2012).

9. Gollner, M. J., Püschel, D., Rydlová, J. & Vosátka, M. Effect of inoculation with soil yeasts on mycorrhizal symbiosis of maize. *Pedobiologia (Jena).* **50,** 341–345 (2006).

10. Hamayun, M. *et al.* Cladosporium sphaerospermum as a new plant growth-promoting endophyte from the roots of Glycine max (L.) Merr. *World J. Microbiol. Biotechnol.* **25,** 627–632 (2009).

11. Nakajima, H., Hamasaki, T., Maeta, S., Kimura, Y. & Takeuchi, Y. A plant growth regulator produced by the fungus, Cochliobolus spicifer. *Phytochemistry* **29,** 1739–1743 (1990).

12. Rodriguez, R. J. *et al.* Stress tolerance in plants via habitat-adapted symbiosis. *ISME J.* **2,** 404–416 (2008).

13. Cloete, K. J., Valentine, A. J., Stander, M. A., Blomerus, L. M. & Botha, A. Evidence of Symbiosis Between the Soil Yeast Cryptococcus laurentii and a Sclerophyllous Medicinal Shrub, Agathosma betulina (Berg.) Pillans. *Microb. Ecol.* **57,** 624–632 (2009).

14. Nutaratat, P., Srisuk, N., Arunrattiyakorn, P. & Limtong, S. Plant growth-promoting traits of epiphytic and endophytic yeasts isolated from rice and sugar cane leaves in Thailand. *Fungal Biol.* **118,** 683–694 (2014).

15. Fávaro, L. C. de L., Sebastianes, F. L. de S. & Araújo, W. L. Epicoccum nigrum P16, a Sugarcane Endophyte, Produces Antifungal Compounds and Induces Root Growth. *PLoS One* **7,** e36826 (2012).

16. Ogórek, R. & Plaskowska, E. Epicoccum nigrum for biocontrol agents in vitro of plant fungal pathogens. *Commun. Agric. Appl. Biol. Sci.* **76,** 691–7 (2011).

17. Li, T. *et al.* Improved tolerance of maize (Zea mays L.) to heavy metals by colonization of a dark septate endophyte (DSE) Exophiala pisciphila. *Sci. Total Environ.* **409,** 1069–1074 (2011).

18. Elsharkawy, M. M., Shimizu, M., Takahashi, H. & Hyakumachi, M. The plant growth-promoting fungus Fusarium equiseti and the arbuscular mycorrhizal fungus Glomus mosseae induce systemic resistance against Cucumber mosaic virus in cucumber plants. *Plant Soil* **361,** 397–409 (2012).

19. Khan, S. *et al.* Plant growth promotion and Penicillium citrinum. *BMC Microbiol.* **8,** 231 (2008).

20. Brian, P. W., Elson, G. W., Hemming, H. G. & Radley, M. The plant-growth-promoting properties of gibberellic acid, a metabolic product of the fungus gibberella fujikuroi. *J. Sci. Food Agric.* **5,** 602–612 (1954).

21. Azevedo, L. C. B. de, Stürmer, S. L. & Lambais, M. R. Early changes in arbuscular mycorrhiza development in sugarcane under two harvest management systems. *Brazilian J. Microbiol.* **45,** 995–1005 (2014).

22. Duponnois, R., Colombet, A., Hien, V. & Thioulouse, J. The mycorrhizal fungus Glomus intraradices and rock phosphate amendment influence plant growth and microbial activity in the rhizosphere of Acacia holosericea. *Soil Biol. Biochem.* **37,** 1460–1468 (2005).

23. Khan, A. L. *et al.* Mutualistic association of Paecilomyces formosus LHL10 offers thermotolerance to Cucumis sativus. *Antonie Van Leeuwenhoek* **101,** 267–279 (2012).

24. Whitelaw, M. A., Harden, T. J. & Helyar, K. R. Phosphate solubilisation in solution culture by the soil fungus Penicillium radicum. *Soil Biol. Biochem.* **31,** 655–665 (1999).

25. Whitelaw, M. A., Harden, T. J. & Bender, G. L. Plant growth promotion of wheat inoculated with Penicillium radicum sp. nov. *Aust. J. Soil Res.* **35,** 291 (1997).

26. Hossain, M. M., Sultana, F., Kubota, M., Koyama, H. & Hyakumachi, M. The Plant Growth-Promoting Fungus Penicillium simplicissimum GP17-2 Induces Resistance in Arabidopsis thaliana by Activation of Multiple Defense Signals. *Plant Cell Physiol.* **48,** 1724–1736 (2007).

27. Chandanie, W. A., Kubota, M. & Hyakumachi, M. Interactions between the arbuscular mycorrhizal fungus Glomus mosseae and plant growth-promoting fungi and their significance for enhancing plant growth and suppressing damping-off of cucumber (Cucumis sativus L.). *Appl. Soil Ecol.* **41,** 336–341 (2009).

28. Meera, M. S., Shivanna, M. B., Kageyama, K. & Hyakumachi, M. Responses of cucumber cultivars to induction of systemic resistance against anthracnose by plant growth promoting fungi. *Eur. J. Plant Pathol.* **101,** 421–430 (1995).

29. Koike, N., Hyakumachi, M., Kageyama, K., Tsuyumu, S. & Doke, N. Induction of systemic resistance in cucumber against several diseases by plant growth-promoting fungi: Lignification and superoxide generation. *Eur. J. Plant Pathol.* **107,** 523–533 (2001).

30. Hamayun, M. *et al.* Phoma herbarum as a New Gibberellin-Producing and Plant Growth-Promoting Fungus. *J. Microbiol. Biotechnol.* **19,** 1244–1249 (2009).

31. Hamayun, M. *et al.* Growth promotion of cucumber by pure cultures of gibberellin-producing Phoma sp. GAH7. *World J. Microbiol. Biotechnol.* **26,** 889–894 (2010).

32. Padmavathi, T., Dikshit, R. & Seshagiri, S. Effect of Rhizophagus spp. and plant growth-promoting Acinetobacter junii on Solanum lycopersicum and Capsicum annuum. *Brazilian J. Bot.* **38,** 273–280 (2015).

33. Li, T. *et al.* Relative importance of an arbuscular mycorrhizal fungus (Rhizophagus intraradices) and root hairs in plant drought tolerance. *Mycorrhiza* **24,** 595–602 (2014).

34. Colombo, R. P. *et al.* Differential effects of two strains of Rhizophagus intraradices on dry biomass and essential oil yield and composition in Calamintha nepeta. *Rev. Argent. Microbiol.* **45,** 114–118 (2013).

35. Yamagiwa, Y. *et al.* Talaromyces wortmannii FS2 emits β-caryphyllene, which promotes plant growth and induces resistance. *J. Gen. Plant Pathol.* **77,** 336–341 (2011).

36. Li, R.-X. *et al.* Solubilisation of Phosphate and Micronutrients by Trichoderma harzianum and Its Relationship with the Promotion of Tomato Plant Growth. *PLoS One* **10,** e0130081 (2015).

37. Duffy, B. Combination of Trichoderma koningii with Fluorescent Pseudomonads for Control of Take-all on Wheat. *Phytopathology* **86,** 188 (1996).

38. Ousley, M. A., Lynch, J. M. & Whipps, J. M. Effect of Trichoderma on plant growth: A balance between inhibition and growth promotion. *Microb. Ecol.* **26,** (1993).

39. Contreras-Cornejo, H. A., Macias-Rodriguez, L., Cortes-Penagos, C. & Lopez-Bucio, J. Trichoderma virens, a Plant Beneficial Fungus, Enhances Biomass Production and Promotes Lateral Root Growth through an Auxin-Dependent Mechanism in Arabidopsis. *PLANT Physiol.* **149,** 1579–1592 (2009).

40. Hermosa, R., Viterbo, A., Chet, I. & Monte, E. Plant-beneficial effects of Trichoderma and of its genes. *Microbiology* **158,** 17–25 (2012).

Unlocking the bacterial and fungal communities assemblages of sugarcane microbiome

Rafael Soares Correa de Souza[1], Vagner Katsumi Okura[1], Jaderson Silveira Leite Armanhi[1], Beatriz Jorrín[2], Núria Lozano[2], Márcio José da Silva[1], Manuel González-Guerrero[2], Laura Migliorini de Araújo[1], Natália Ferreira Verza[1], Homayoun C. Bagheri[3], Juan Imperial[2,4] and Paulo Arruda[1,5,*]

[1]Centro de Biologia Molecular e Engenharia Genética, Universidade Estadual de Campinas (UNICAMP), 13083-875, Campinas, SP, Brazil

[2]Centro de Biotecnología y Genómica de Plantas (CBGP). Universidad Politécnica de Madrid (UPM), 28223 Pozuelo de Alarcón, Madrid, Spain

[3]New Energies Division, Repsol Technology Center, 28935 Mostoles-Madrid, Spain

[4] Consejo Superior de Investigaciones Científicas, Madrid, Spain

[5]Departamento de Genética e Evolução, Instituto de Biologia, Universidade Estadual de Campinas (UNICAMP), 13083-875, Campinas, SP, Brazil

Supplementary Data

Supplementary Data

PCR 1 - PRIMERS FOR V4 REGION OF 16S GENE

Oligo	Orientation	Appends			Complete Sequence (5'->3')
		Append to 5' (Nextera transposase sequence)	rame Shift	primer (rRNA)	
515f_FS1	Forward	TCGTCGGCAGCGTCAGATGTGTATAAGAGACAG	NNNNNN	GTGCCAGCMGCCGCGGTAA	TCGTCGGCAGCGTCAGATGTGTATAAGAGACAGNNNNNNGTGCCAGCMGCCGCGGTAA
515f_FS2	Forward	TCGTCGGCAGCGTCAGATGTGTATAAGAGACAG	NNNNN	GTGCCAGCMGCCGCGGTAA	TCGTCGGCAGCGTCAGATGTGTATAAGAGACAGNNNNNGTGCCAGCMGCCGCGGTAA
515f_FS3	Forward	TCGTCGGCAGCGTCAGATGTGTATAAGAGACAG	NNNN	GTGCCAGCMGCCGCGGTAA	TCGTCGGCAGCGTCAGATGTGTATAAGAGACAGNNNNGTGCCAGCMGCCGCGGTAA
806r_FS	Reverse	GTCTCGTGGGCTCGGAGATGTGTATAAGAGACAG	NNNNNN	GGACTACHVGGGTWTCTAAT	GTCTCGTGGGCTCGGAGATGTGTATAAGAGACAGNNNNNNGGACTACHVGGGTWTCTAAT

PCR 1 - PRIMERS FOR ITS GENE

Oligo	Orientation	Appends			Complete Sequence (5'->3')
		Append to 5' (Nextera transposase sequence)	rame Shift	primer (rRNA)	
ITS9f_FS1	Forward	TCGTCGGCAGCGTCAGATGTGTATAAGAGACAG	NNNNNN	GAACGCAGCRAAIIGYGA	TCGTCGGCAGCGTCAGATGTGTATAAGAGACAGNNNNNNGAACGCAGCRAAIIGYGA
ITS9f_FS2	Forward	TCGTCGGCAGCGTCAGATGTGTATAAGAGACAG	NNNNN	GAACGCAGCRAAIIGYGA	TCGTCGGCAGCGTCAGATGTGTATAAGAGACAGNNNNNGAACGCAGCRAAIIGYGA
ITS9f_FS3	Forward	TCGTCGGCAGCGTCAGATGTGTATAAGAGACAG	NNNN	GAACGCAGCRAAIIGYGA	TCGTCGGCAGCGTCAGATGTGTATAAGAGACAGNNNNGAACGCAGCRAAIIGYGA
ITS4r_FS	Reverse	GTCTCGTGGGCTCGGAGATGTGTATAAGAGACAG	NNNNNN	TCCTCCGCTTATTGATATGC	GTCTCGTGGGCTCGGAGATGTGTATAAGAGACAGNNNNNNTCCTCCGCTTATTGATATGC

i5 PRIMERS

Oligo	Sequence (5'->3')
S501	AATGATACGGCGACCACCGAGATCTACACTAGATCGCTCGTCGGCAGCGTC
S502	AATGATACGGCGACCACCGAGATCTACACCTCTCTATTCGTCGGCAGCGTC
S503	AATGATACGGCGACCACCGAGATCTACACTATCCTCTTCGTCGGCAGCGTC
S504	AATGATACGGCGACCACCGAGATCTACACAGAGTAGATCGTCGGCAGCGTC
S505	AATGATACGGCGACCACCGAGATCTACACGTAAGGAGTCGTCGGCAGCGTC
S506	AATGATACGGCGACCACCGAGATCTACACACTGCATATCGTCGGCAGCGTC
S507	AATGATACGGCGACCACCGAGATCTACACAAGGAGTATCGTCGGCAGCGTC
S508	AATGATACGGCGACCACCGAGATCTACACCTAAGCCTTCGTCGGCAGCGTC

i7 PRIMERS

Oligo	Sequence (5'->3')
N701	CAAGCAGAAGACGGCATACGAGATTCGCCTTAGTCTCGTGGGCTCGG
N702	CAAGCAGAAGACGGCATACGAGATCTAGTACGGTCTCGTGGGCTCGG
N703	CAAGCAGAAGACGGCATACGAGATTTCTGCCTGTCTCGTGGGCTCGG
N704	CAAGCAGAAGACGGCATACGAGATGCTCAGGAGTCTCGTGGGCTCGG
N705	CAAGCAGAAGACGGCATACGAGATAGGAGTCCGTCTCGTGGGCTCGG
N706	CAAGCAGAAGACGGCATACGAGATCATGCCTAGTCTCGTGGGCTCGG
N707	CAAGCAGAAGACGGCATACGAGATGTAGAGAGGTCTCGTGGGCTCGG
N708	CAAGCAGAAGACGGCATACGAGATCCTCTCTGGTCTCGTGGGCTCGG
N709	CAAGCAGAAGACGGCATACGAGATAGCGTAGCGTCTCGTGGGCTCGG
N710	CAAGCAGAAGACGGCATACGAGATCAGCCTCGGTCTCGTGGGCTCGG
N711	CAAGCAGAAGACGGCATACGAGATTGCCTCTTGTCTCGTGGGCTCGG
N712	CAAGCAGAAGACGGCATACGAGATTCCTCTACGTCTCGTGGGCTCGG
